# Global, regional, and national burden of other musculoskeletal disorders, 1990–2020, and projections to 2050: a systematic analysis of the Global Burden of Disease Study 2021

**DOI:** 10.1016/S2665-9913(23)00232-1

**Published:** 2023-10-23

**Authors:** GBD 2021 Other Musculoskeletal Disorders Collaborators, Tiffany K Gill, Manasi Murthy Mittinty, Lyn M March, Jaimie D Steinmetz, Garland T Culbreth, Marita Cross, Jacek A Kopec, Anthony D Woolf, Lydia M Haile, Hailey Hagins, Kanyin Liane Ong, Deborah R Kopansky-Giles, Karsten E Dreinhoefer, Neil Betteridge, Mohammadreza Abbasian, Mitra Abbasifard, krishna Abedi, Miracle Ayomikun Adesina, Janardhana P Aithala, Mostafa Akbarzadeh-Khiavi, Yazan Al Thaher, Tariq A Alalwan, Hosam Alzahrani, Sohrab Amiri, Benny Antony, Jalal Arabloo, Aleksandr Y Aravkin, Ashokan Arumugam, Krishna K Aryal, Seyyed Shamsadin Athari, Alok Atreya, Soroush Baghdadi, Mainak Bardhan, Lope H Barrero, Lindsay M Bearne, Alehegn Bekele Bekele, Isabela M Bensenor, Pankaj Bhardwaj, Rajbir Bhatti, Ali Bijani, Theresa Bordianu, Souad Bouaoud, Andrew M Briggs, Huzaifa Ahmad Cheema, Steffan Wittrup McPhee Christensen, Isaac Sunday Chukwu, Benjamin Clarsen, Xiaochen Dai, Katie de Luca, Belay Desye, Meghnath Dhimal, Thanh Chi Do, Adeniyi Francis Fagbamigbe, Siamak Farokh Forghani, Nuno Ferreira, Balasankar Ganesan, Mesfin Gebrehiwot, Ahmad Ghashghaee, Simon Matthew Graham, Netanja I Harlianto, Jan Hartvigsen, Ahmed I Hasaballah, Mohammad Hasanian, Mohammed Bheser Hassen, Simon I Hay, Mohammad Heidari, Alexander Kevin Hsiao, Irena M Ilic, Mohammad Jokar, Himanshu Khajuria, Md Jobair Khan, Praval Khanal, Sorour Khateri, Ali Kiadaliri, Min Seo Kim, Adnan Kisa, Ali-Asghar Kolahi, Kewal Krishan, Vijay Krishnamoorthy, Iván Landires, Bagher Larijani, Thao Thi Thu Le, Yo Han Lee, Stephen S Lim, Justin Lo, Seyedeh Panid Madani, Jeadran N Malagón-Rojas, Iram Malik, Hamid Reza Marateb, Ashish J Mathew, Tuomo J Meretoja, Mohamed Kamal Mesregah, Tomislav Mestrovic, Alireza Mirahmadi, Awoke Misganaw, Sadra Mohaghegh, Ali H Mokdad, Kaveh Momenzadeh, Sara Momtazmanesh, Lorenzo Monasta, Mohammad Ali Moni, Yousef Moradi, Ebrahim Mostafavi, Jibran Sualeh Muhammad, Christopher J L Murray, Sathish Muthu, Shumaila Nargus, Hasan Nassereldine, Subas Neupane, Robina Khan Niazi, In-Hwan Oh, Hassan Okati-Aliabad, Abderrahim Oulhaj, Kevin Pacheco-Barrios, Seoyeon Park, Jay Patel, Shrikant Pawar, Paolo Pedersini, Mario F P Peres, Ionela-Roxana Petcu, Fanny Emily Petermann-Rocha, Mohsen Poursadeqiyan, Ibrahim Qattea, Maryam Faiz Qureshi, Quinn Rafferty, Shahram Rahimi-Dehgolan, Mosiur Rahman, Shakthi Kumaran Ramasamy, Vahid Rashedi, Elrashdy Moustafa Mohamed Redwan, Daniel Cury Ribeiro, Leonardo Roever, Azam Safary, Dominic Sagoe, Fatemeh Saheb Sharif-Askari, Amirhossein Sahebkar, Sana Salehi, Amir Shafaat, Saeed Shahabi, Saurab Sharma, Bereket Beyene Shashamo, Rahman Shiri, Ambrish Singh, Helen Slater, Amanda E Smith, Dev Ram Sunuwar, Mohammad Tabish, Samar Tharwat, Irfan Ullah, Sahel Valadan Tahbaz, Tommi Juhani Vasankari, Jorge Hugo Villafañe, Stein Emil Vollset, Taweewat Wiangkham, Naohiro Yonemoto, Yuyi You, Iman Zare, Peng Zheng, Theo Vos, Peter M Brooks

## Abstract

**Background:**

Musculoskeletal disorders include more than 150 different conditions affecting joints, muscles, bones, ligaments, tendons, and the spine. To capture all health loss from death and disability due to musculoskeletal disorders, the Global Burden of Diseases, Injuries, and Risk Factors Study (GBD) includes a residual musculoskeletal category for conditions other than osteoarthritis, rheumatoid arthritis, gout, low back pain, and neck pain. This category is called other musculoskeletal disorders and includes, for example, systemic lupus erythematosus and spondylopathies. We provide updated estimates of the prevalence, mortality, and disability attributable to other musculoskeletal disorders and forecasted prevalence to 2050.

**Methods:**

Prevalence of other musculoskeletal disorders was estimated in 204 countries and territories from 1990 to 2020 using data from 68 sources across 23 countries from which subtraction of cases of rheumatoid arthritis, osteoarthritis, low back pain, neck pain, and gout from the total number of cases of musculoskeletal disorders was possible. Data were analysed with Bayesian meta-regression models to estimate prevalence by year, age, sex, and location. Years lived with disability (YLDs) were estimated from prevalence and disability weights. Mortality attributed to other musculoskeletal disorders was estimated using vital registration data. Prevalence was forecast to 2050 by regressing prevalence estimates from 1990 to 2020 with Socio-demographic Index as a predictor, then multiplying by population forecasts.

**Findings:**

Globally, 494 million (95% uncertainty interval 431–564) people had other musculoskeletal disorders in 2020, an increase of 123·4% (116·9–129·3) in total cases from 221 million (192–253) in 1990. Cases of other musculoskeletal disorders are projected to increase by 115% (107–124) from 2020 to 2050, to an estimated 1060 million (95% UI 964–1170) prevalent cases in 2050; most regions were projected to have at least a 50% increase in cases between 2020 and 2050. The global age-standardised prevalence of other musculoskeletal disorders was 47·4% (44·9–49·4) higher in females than in males and increased with age to a peak at 65–69 years in male and female sexes. In 2020, other musculoskeletal disorders was the sixth ranked cause of YLDs globally (42·7 million [29·4–60·0]) and was associated with 83 100 deaths (73 600–91 600).

**Interpretation:**

Other musculoskeletal disorders were responsible for a large number of global YLDs in 2020. Until individual conditions and risk factors are more explicitly quantified, policy responses to this burden remain a challenge. Temporal trends and geographical differences in estimates of non-fatal disease burden should not be overinterpreted as they are based on sparse, low-quality data.

**Funding:**

Bill & Melinda Gates Foundation.

## Introduction

In 2020, musculoskeletal disorders were the second-highest ranked cause of non-fatal disability^1^ and affected more than 1·63 billion people worldwide. The Global Burden of Diseases, Injuries, and Risk Factors Study (GBD) includes five specific musculoskeletal conditions: rheumatoid arthritis, osteoarthritis, low back pain, neck pain, and gout. A sixth residual category of other musculoskeletal disorders is composed of a wide range of other acute and chronic conditions that affect the locomotor and connective tissue system, including bones, joints, ligaments, tendons, and muscles. This heterogeneous category includes spondyloarthropathies; inflammatory arthritis other than rheumatoid arthritis, such as psoriatic arthritis; vasculitis; autoimmune conditions such as systemic lupus erythematosus; chronic musculoskeletal pain syndromes such as fibromyalgia; other osteopathies, chondropathies, disorders of bone density and structure and disorders of synovium, tendons, and connective tissue; and other undefined disorders of the musculoskeletal system and connective tissue that are not explicitly modelled in the GBD.

**Research in context**

**Evidence before this study**

Other musculoskeletal disorders is a heterogeneous category comprising a wide range of disorders of muscles, bones, joints, connective tissues, and ligaments that are not included in the five musculoskeletal diseases (rheumatoid arthritis, osteoarthritis, low back pain, neck pain, and gout) defined by the Global Burden of Diseases, Injuries, and Risk Factors Study (GBD) and are not captured as long-term sequelae of injuries. The global effect of these musculoskeletal conditions—as measured by prevalence, years of life lived with disability, and disability-adjusted life-years—has been shown in previous GBD studies to be large. These disorders have a large non-fatal burden, but estimation is hampered by low-quality data from heterogeneous sources. For these estimates, we identified sources following the method used for GBD 2017, updating the search for this iteration. We selected from studies that identified musculoskeletal conditions—largely the Community Oriented Program for the Control of Rheumatic Diseases (COPCORD) surveys—which enabled us to subtract the prevalence of the five explicitly quantified musculoskeletal disorders (defined above) from the total prevalence of musculoskeletal disorders. These data were supplemented with insurance claims data in the USA and from the Poland National Health Fund for which International Classification of Disease (ICD) codes were available.

**Added value of this study**

We highlight the substantial burden of other musculoskeletal disorders that would otherwise go unrecognised, while also cautioning against overinterpreting findings for this heterogeneous residual cause in the GBD given the sparse, low-quality data available. Other musculoskeletal disorders are evidently a large source of disability in the world, and do cause deaths, although most of the burden is non-fatal. Forecasts also indicate that the number of people worldwide living with these conditions, and the effect of these conditions on the global population, will increase. Compared with previous estimates for other musculoskeletal disorders using GBD 2017 data, this update includes 2 more years of prevalence data obtained from insurance claims in the USA (2015–2016), newly added prevalence data from insurance claims in Poland during 2015–2017, more years of vital registration death data, updated methods to better estimate patterns in non-fatal disease burden by age and sex and to redistribute poorly coded death data, and prevalence forecasts to the year 2050.

**Implications of all the available evidence**

Other musculoskeletal disorders are common reasons for seeking health care. Notably, the age-standardised prevalence of these disorders was highest in high-income regions (eg, high income North America [11 100 cases per 100 000 people, 95% uncertainty interval 10 500–11 800]); however, this finding might reflect the geographical distribution of the available data rather than true variation. The true extent of the burden and its geographical patterns will require additional analytical efforts to separate out specific diseases from this residual category and treat these diseases as specific causes with their own data sources and methods. Introducing a new cause into the GBD is resource-intensive as it requires collecting data via systematic reviews, making data adjustments for multiple case definitions, constructing analytical models, and calculating severity distributions with appropriate disability weights. Given the large burden of other musculoskeletal disorders and the disparate nature of the diseases included, an expanded programme of work is advisable to address the issue.

The GBD systematically quantifies health loss for more than 350 diseases by age, sex, year, and geographical location and enables the comparison of burden for a wide range of conditions. To estimate health loss in populations as comprehensively as possible, GBD uses 41 residual causes, which are often a collection of heterogeneous, smaller conditions within a larger cause group. Estimates for these residual categories are more uncertain than those for specific diseases.

In this study, we report the burden of other musculoskeletal disorders within the GBD framework, provide regional and national disability estimates of other musculoskeletal disorders from 1990 to 2020 and forecasts up to 2050, and discuss the challenges and limitations in reporting and estimating the disease burden of this residual category of musculoskeletal disorders.

## Methods

### Overview

This manuscript was produced as part of the GBD Collaborator Network and in accordance with the GBD Protocol. Mortality was estimated using CODEm, GBD's analytical tool for cause of death estimation, and incidence and prevalence attributable to other musculoskeletal disorders were estimated using a Bayesian meta-regression tool, DisMod-MR 2.1, by year, age, and sex for 204 countries and territories. The GBD Study adheres to the Guidelines for Accurate and Transparent Health Estimates Reporting statement.^2^

### Case definition

Other musculoskeletal disorders is a heterogeneous category of conditions that remain after the five musculoskeletal disorders defined in the GBD (rheumatoid arthritis, osteoarthritis, low back pain, neck pain, and gout) and the long-term sequelae of injuries have been separated out. Prevalence data for these conditions come from population-based health surveys and from insurance claims records for the following International Classification of Disease (ICD) codes (appendix p 8): systemic lupus erythematosus/ lupus erythematosus (ICD-9 710.0 or ICD-10 L93), infectious arthropathies (ICD-9 711 or ICD-10 M00–M02), inflammatory polyarthropathies (ICD-9 712–713 or 446, ICD-10 M03, M06-M09 or M11–M13), other joint disorders (ICD-9 716–719 or ICD-10 M20–M25), systemic connective tissue disorders (ICD-9 710.1–710.9 or ICD-10 M30–M36), deforming dorsopathies (ICD-9 737 or 416.1, or ICD-10 M40–M43 or I27.1), spondylopathies (ICD-9 720 or ICD-10 M45–M46), disorders of muscles (ICD-9 725 or ICD-10 M61–M63), disorders of the synovium and tendons (ICD-9 726–728 or ICD-10 M65–M68), other soft tissue disorders (ICD-9 729, ICD-10 M70–M73 or M75–M79), disorders of bone density and structure (ICD-9 733.0–733.2 or ICD-10 M80–M85), osteomyelitis (ICD-9 730.1–730.3 or 730.7–730.9 or ICD-10 M86), other osteopathies (ICD-9 733.3–733.9 or ICD-10 M87–M90), chondropathies (ICD-9 732 or ICD-10 M91–M94), and other disorders of the musculoskeletal system and connective tissue (ICD-9 734–736 or 738-739, ICD-10 M95 or M99). Both population-based health surveys and insurance claims data were treated as reference definition data.

### Input data

A systematic review, done for GBD 2017^3^ and updated for this iteration, was conducted to identify population-based health surveys that measured the total prevalence of any musculoskeletal disorder or symptom and distinguished the residual category after accounting for osteoarthritis, rheumatoid arthritis, gout, low back pain, or neck pain. In addition, ICD codes were used to extract the prevalence of other musculoskeletal disorders from insurance claims data in the USA (for 2000 and 2010–16) and from the Poland National Health Fund (for 2015–17).

We reviewed data from the Russia Statistical Yearbook from 2010, selecting conditions that are part of the other musculoskeletal disorders category; however, these data were excluded for being implausibly low (more than ten times lower than the regional fit). Data from Taiwan (province of China) insurance claims for 2016 were also considered, but were excluded for being implausibly high (four times higher than the model regional fit).

We included 68 data sources for other musculoskeletal disorders from 23 countries. The data sources used to estimate the non-fatal and fatal data disease burden of other musculoskeletal disorders are shown in the appendix (pp 9–12, 42).

### Data processing and disease modelling

If prevalence data were reported only for male and female sexes combined or in age groups spanning more than 20 years, these data were split by sex and into more granular age groups. First, if studies reported prevalence for broad age groups by sex and also for specific age groups but for male and female sexes combined, age-specific estimates were split by sex using the reported within-study sex ratio. Second, prevalence data for male and female sexes combined that could not be split using a within-study ratio were adjusted using a sex ratio derived from a meta-analysis of existing sex-specific data in the other musculoskeletal disorders dataset using a metaregression tool, MRTool 0.0.1.^4^ The female-to-male ratio resulting from this analysis was 1·37 (95% uncertainty interval [UI] 1·37–1·38). Finally, if studies reported estimates across age groups spanning 25 years or more, these estimates were split into 5-year age groups using the prevalence age pattern estimated in GBD 2017.^1^

Input data were entered into a Bayesian meta-regression tool, DisMod-MR 2.1,^5^, ^6^ to generate estimates of prevalence by age, sex, and geographical location for 1990–2020. Previous settings in the model included the assumption of no incidence or prevalence of other musculoskeletal disorders before the age of 10 years. In the absence of any meaningful data on incidence or remission for such a heterogeneous category of disorders, we assumed a remission of 0·5–1·0, constituting an average duration of 1–2 years. Socio-demographic Index (SDI)^6^—a composite index of total fertility rate in women younger than 25 years, mean education for people aged 15 years and older, and lag-distributed income per capita—was included in the model as a predictive covariate for prevalence (appendix p 12). Fatal input data were excluded from the DisMod model considering that the pattern of mortality comes from autoimmune diseases and infections of the bones and joints, which constitute only a small fraction of the non-fatal manifestations captured in this residual category.

To reflect the range of severity of other musculoskeletal disorders, we selected six categories of disease severity and health state levels for rheumatoid arthritis and osteoarthritis. This severity distribution was derived from analysis of the Medical Expenditure Panel Surveys (MEPS) in the USA between 2001 and 2014.^7^ MEPS respondents reported on the reasons for their health-care contact over a 2-year period, which were then coded according to ICD-9. Over the 2-year follow-up, respondents filled out a Short Form 12 questionnaire (SF-12)^8^ twice. We predicted the relationship between SF-12 scores and GBD disability weights on the basis of a purposive survey among staff of the Institute for Health Metrics and Evaluation, who completed the SF-12 for 60 health states and corresponding lay descriptions in GBD ranging from mild to very severe. Thus, for each MEPS respondent, we calculated the disability weight value they had indirectly reported on SF-12 and computed the contribution from other musculoskeletal disorders by correcting for any comorbid conditions contributing to that individual's experience of health loss.^9^ Respondents were then categorised into seven health-state bins—the six musculoskeletal health states with disability weights plus one asymptomatic state—on the basis of the amount of disability weight assigned to other musculoskeletal disorders, with the midpoint between disability weights taken to be the threshold between severity levels. The proportions of respondents in each bin were then multiplied by the prevalence of other musculoskeletal disorders from the DisMod-MR 2.1 model.

Descriptions of health states and their disability weights can be found in the appendix (pp 1–2, 13–14). To estimate years lived with disability (YLDs) for other musculoskeletal disorders, we multiplied the prevalence of each severity level by the corresponding disability weight for each year, age, sex, and location. Uncertainty was incorporated by running 100 realisations (draws) of the converged DisMod 2.1 model and of the proportions in each health state of the MEPS analysis and their corresponding disability weights. Uncertainty intervals were then calculated using the 2·5th and 97·5th percentiles of the draws.

As the GBD study is structured such that causes of health burden are considered mutually exclusive, other musculoskeletal disorders are considered to be separate from fractures and dislocations, for which estimates are produced separately. Because estimates are produced for both fractures and dislocations as well as other musculoskeletal disorders that can include fractures and dislocations, subtracting the portion of other musculoskeletal disorders attributed to fractures and dislocations is necessary to prevent counting them twice.

### Cause of death modelling

Vital registration data were used to estimate deaths and cause-specific mortality rates for other musculoskeletal disorders. The most common ICD codes for this diverse category in vital registration data were those for systemic lupus erythematosus (M321 and M329), unspecified osteoporosis (M809 and M819), systemic sclerosis (M348 and M349), and pyogenic arthritis, unspecified (M009). Data went through a series of adjustments, including misdiagnosis correction for commonly miscoded diseases (dementia, Parkinson's disease, and atrial fibrillation), HIV correction, redistribution to reassign impossible or less specific codes (eg, senility), and noise reduction to deal with small death counts.^5^ Data were then added to Cause of Death Ensemble models (CODEm) to estimate deaths by year, age, sex, and location between 1990 and 2020. Years of life lost (YLLs) were calculated as the number of deaths multiplied by the remaining standard life expectancy at the age of death.^6^ Disability-adjusted life-years (DALYs), a summary measure of total non-fatal and fatal disease burden, were then computed by summing YLDs and YLLs for each year, age, sex, and location.

### Forecasting prevalence

Forecasted global and regional cases of other musculoskeletal disorders to 2050 were computed by forecasting prevalence rates and population estimates. For the combined other musculoskeletal disorders category, age-specific, location-specific, and sex-specific GBD 2019 prevalence from 1990 to 2020 were logit-transformed and used in the following regression model:

*E*[logit(*Y*_l,a,s,y_)]=β_1_SDIα_l,a,s_.

The term on the left-hand side of the equation is the forecasted logit(prevalence), where *Y* is prevalence; *l, a, s*, and *y* represent location, age, sex, and year; β_1_ is the fixed coefficient on SDI over time; α_l,a,s_ is the location-age-sex-specific random intercept, and *E* is the expected value. To obtain forecasted cases, forecasted rates were multiplied by forecasted population counts.^10^ Forecasted prevalence rates were intercept-shifted to GBD prevalence by subtracting forecasted estimation year 2020 prevalence rates from GBD estimation year 2020 prevalence rates, and using this difference to shift all forecasted values through to 2050. This shifting was necessary because the original forecast model was produced using GBD 2019 estimates.

A Das Gupta decomposition analysis was done to identify the relative contributions of population growth, population ageing, and changes in prevalence unrelated to demographics to the change in case number between 2020 and 2050.^11^

Internal validation was conducted using forecasts for osteoarthritis, which used the same forecasting model. The tests used estimates from 1990 to 2010 as training data to project prevalence from 2010 to 2019 by age, sex, location, and year, and then compared the projected prevalence with GBD results for this period. We evaluated the accuracy of the projections by calculating the summary root-mean-squared error and bias, where bias was calculated as the median value of all predicted minus observed values by age, sex, location, and year. In all four tests the model root-mean-squared error was less than 0·01 and bias was less than 0·0001 (appendix p 14).

### Role of the funding source

The funder of the study had no role in study design, data collection, data analysis, data interpretation, or writing of the report.

## Results

**Figure 1:**
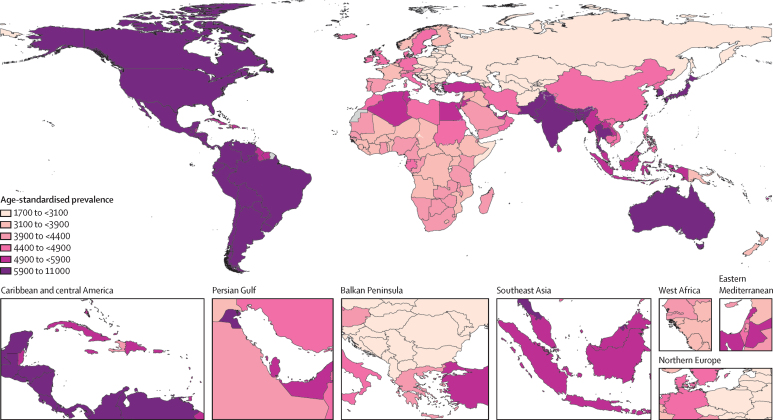
Age-standardised prevalence of other musculoskeletal disorders for male and female sexes combined, 2020

In 2020, there were an estimated 494 million (95% UI 431–564) people with other musculoskeletal disorders globally, a substantial increase (123·4% [116·9–129·3]) since 1990 (221 million [192–253]; appendix pp 15–24). Globally, the prevalence (all ages) of other musculoskeletal disorders was estimated at 6320 (5510–7220) per 100 000 people in 2020, and 4140 (3590–4740) in 1990. The age-standardised prevalences, per 100 000 people, were 5910 (5180–6750) in 2020 (figure 1; appendix pp 15–24) and 4720 (4090–5450) in 1990. To avoid double counting, we subtracted prevalent cases of long-term disability from fractures and dislocations out of the estimates: 69·0 million (65·0–73·0) cases were subtracted for females and 84·4 million (80·1–88·7) for males. These cases accounted for 19·8% (17·4–22·3) of cases of other musculoskeletal disorders in females and 33·1% (29·3–37·0) cases in males before subtraction.

**Figure 2:**
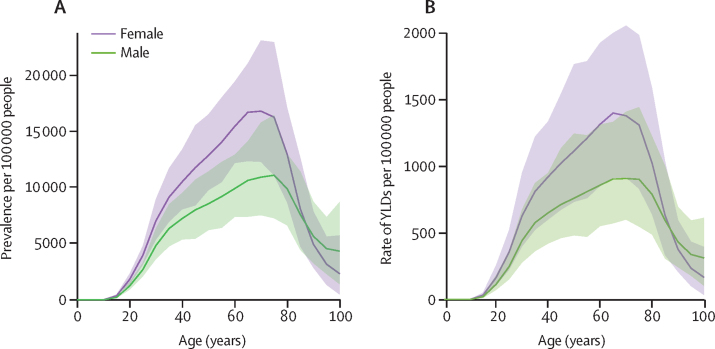
Global prevalence of and rate of YLDs attributed to other musculoskeletal disorders by age and sex in 2020 Prevalence (A) and rate of YLDs (B) per 100 000 people. The shaded regions denote 95% uncertainty interval. YLDs=years lived with disability.

In 2020, other musculoskeletal disorders was the sixth ranked cause of global YLDs, the 117th ranked cause of YLLs, and the 19th ranked cause of DALYs. Global YLDs in 2020 amounted to 42·7 million (95% UI 29·4–60·0; table 1, appendix pp 25–40), with south Asia having the highest number of YLDs (12 800 000 [8 880 000–18 100 000]). The prevalence and YLDs peaked in the 60–69-year age group (figure 2). The global age-standardised prevalence of other musculoskeletal disorders was 47·4% (44·9–49·4) higher in females than in males and increased with age to a peak at 65–69 years in male and female sexes (figure 2A) and the age-standardised rate of YLDs was higher in females (604 per 100 000 people [417–841]) than in males (418 [287–591]; figure 2B).

**Table 1:** Number and age-standardised rate of YLDs for other musculoskeletal disorders in 2020, and percentage change from 1990, by GBD region and super-region

		**Number of YLDs**	**Percentage change in number of YLDs from 1990 to 2020**	**Age-standardised rate of YLDs per 100 000 people in 2020**	**Age-standardised rate of YLDs per 100 000 people in 1990**	**Percentage change in age-standardised rate of YLDs from 1990 to 2020**
**Global**		**42 700 000 (29 400 000 to 60 000 000)**	**122·0 (115·0 to 128·0)**	**512·0 (352·0 to 717·0)**	**409·0 (283·0 to 577·0)**	**25·1 (23·0 to 27·1)**
**Central Europe, eastern Europe, and central Asia**		**911 000 (630 000 to 1 290 000)**	**63·3 (56·0 to 71·8)**	**173·6 (120·1 to 243·8)**	**120·9 (82·1 to 167·1)**	**43·6 (38·0 to 52·4)**
	Central Asia	190 000 (128 000 to 263 000)	114·0 (102·0 to 126·0)	207·0 (141·0 to 281·0)	158·0 (106·0 to 218·0)	30·8 (24·4 to 37·3)
	Central Europe	262 000 (181 000 to 368 000)	56·1 (45·3 to 69·0)	179·0 (123·0 to 248·0)	122·0 (82·7 to 170·0)	47·0 (38·6 to 57·4)
	Eastern Europe	459 000 (319 000 to 661 000)	52·3 (44·7 to 61·0)	161·0 (113·0 to 232·0)	112·0 (75·5 to 159·0)	43·9 (36·6 to 53·5)
**High income**		**9 090 000 (6 190 000 to 12 200 000)**	**76·8 (68·3 to 86·6)**	**670·6 (458·2 to 900·9)**	**497·3 (341·2 to 692·4)**	**34·9 (28·7 to 42·5)**
	Australasia	247 000 (175 000 to 337 000)	116·0 (99·4 to 132·0)	649·0 (457·0 to 871·0)	513·0 (352·0 to 705·0)	26·5 (17·9 to 36·0)
	High-income Asia Pacific	1 920 000 (1 310 000 to 2 560 000)	65·7 (57·1 to 76·5)	720·0 (495·0 to 980·0)	581·0 (400·0 to 804·0)	24·0 (20·8 to 27·5)
	High-income North America	4 180 000 (2 850 000 to 5 560 000)	103·0 (87·6 to 120·0)	951·0 (651·0 to 1270·0)	666·0 (457·0 to 943·0)	42·8 (31·3 to 55·4)
	Southern Latin America	692 000 (486 000 to 959 000)	70·2 (60·5 to 79·3)	908·0 (639·0 to 1260·0)	853·0 (603·0 to 1150·0)	6·5 (0·4 to 12·2)
	Western Europe	2 060 000 (1 380 000 to 2 860 000)	45·3 (37·5 to 52·6)	376·0 (254·0 to 529·0)	314·0 (212·0 to 445·0)	19·6 (14·7 to 25·3)
**Latin America and Caribbean**		**4 040 000 (2 800 000 to 5 740 000)**	**121·9 (115·4 to 129·9)**	**642·0 (444·3 to 907·0)**	**612·7 (427·7 to 868·9)**	**4·8 (3·4 to 6·6)**
	Andean Latin America	354 000 (245 000 to 504 000)	160·0 (151·0 to 172·0)	561·0 (387·0 to 795·0)	497·0 (348·0 to 695·0)	12·9 (8·8 to 16·7)
	Caribbean	221 000 (154 000 to 307 000)	101·0 (91·5 to 109·0)	431·0 (302·0 to 597·0)	364·0 (250·0 to 494·0)	18·4 (13·0 to 22·8)
	Central Latin America	1 870 000 (1 290 000 to 2 660 000)	151·0 (141·0 to 162·0)	723·0 (497·0 to 1020·0)	636·0 (440·0 to 905·0)	13·6 (11·4 to 16·4)
	Tropical Latin America	1 590 000 (1 110 000 to 2 270 000)	91·5 (85·3 to 99·8)	622·0 (433·0 to 881·0)	679·0 (478·0 to 966·0)	−8·3 (−10·5 to −5·7)
**North Africa and Middle East**		**2 410 000 (1 660 000 to 3 540 000)**	**246·0 (231·0 to 260·0)**	**402·0 (280·0 to 579·0)**	**291·0 (199·0 to 421·0)**	**38·1 (33·0 to 42·9)**
**South Asia**		**12 800 000 (8 880 000 to 18 100 000)**	**158·0 (153·0 to 165·0)**	**733·0 (507·0 to 1030·0)**	**605·0 (422·0 to 849·0)**	**21·2 (19·3 to 23·4)**
**Southeast Asia, east Asia, and Oceania**		**11 100 000 (7 570 000 to 16 000 000)**	**115·0 (104·6 to 125·0)**	**416·4 (286·3 to 594·3)**	**348·4 (239·6 to 492·6)**	**19·5 (17·9 to 21·4)**
	East Asia	7 630 000 (5 170 000 to 10 900 000)	107·0 (96·3 to 119·0)	394·0 (271·0 to 563·0)	329·0 (225·0 to 464·0)	20·0 (17·7 to 21·8)
	Oceania	38 000 (26 000 to 53 400)	150·0 (136·0 to 162·0)	347·0 (237·0 to 484·0)	331·0 (226·0 to 456·0)	4·9 (−0·3 to 9·3)
	Southeast Asia	3 440 000 (2 370 000 to 4 960 000)	131·0 (122·0 to 140·0)	474·0 (327·0 to 674·0)	410·0 (285·0 to 582·0)	15·4 (12·8 to 18·2)
**Sub-Saharan Africa**		**2 360 000 (1 620 000 to 3 310 000)**	**169·6 (162·9 to 174·0)**	**323·0 (221·4 to 450·1)**	**285·6 (196·1 to 397·6)**	**13·1 (11·1 to 14·8)**
	Central sub-Saharan Africa	264 000 (182 000 to 375 000)	172·0 (154·0 to 189·0)	303·0 (210·0 to 422·0)	286·0 (194·0 to 390·0)	6·2 (−0·3 to 12·2)
	Eastern sub-Saharan Africa	822 000 (562 000 to 1 170 000)	173·0 (162·0 to 181·0)	305·0 (208·0 to 428·0)	271·0 (186·0 to 380·0)	12·7 (8·5 to 15·5)
	Southern sub-Saharan Africa	257 000 (177 000 to 363 000)	136·0 (128·0 to 147·0)	355·0 (245·0 to 495·0)	294·0 (202·0 to 420·0)	21·1 (17·3 to 26·2)
	Western sub-Saharan Africa	1 010 000 (695 000 to 1 420 000)	182·0 (175·0 to 188·0)	335·0 (230·0 to 466·0)	296·0 (204·0 to 408·0)	13·4 (11·7 to 15·2)

Data in parentheses are 95% uncertainty intervals. North Africa and Middle East and South Asia are both super-regions and regions. The sum of regional YLDs might not exactly match the total for the super-regions because of rounding. YLDs=years lived with disability.

In insurance claims data from the USA, which were the largest source of input data, other joint disorders (32·2% of all ICD-9 and ICD-10 codes), biomechanical lesions (28·8%), other soft tissue disorders (16·2%), and disorders of the synovium and tendons (10·7%) were the most common diseases and syndromes within the other musculoskeletal disorders category, accounting for more than 85% of all ICD codes assigned to this category.

Globally there were 83 100 deaths (95% UI 73 600 to 91 600) from other musculoskeletal disorders in 2020 (table 2, appendix pp 15–24), with south Asia having the highest number of deaths (21 700 [16 500 to 26 300]). The number of deaths was higher for females (58 900 [50 000 to 64 100]) than for males (24 200 [17 400 to 27 600]). Among all vital registration data for other musculoskeletal disorders, systemic lupus erythematosus and unspecified osteoporosis were the most common ICD-coded causes of death. Other musculoskeletal disorders caused 2·21 million (1·92 to 2·42) YLLs in 2020. Compared with 1990, the global age-standardised rate of YLLs decreased by 5·9% (−15·4 to 1·9), from 28·9 per 100 000 people (26·5 to 31·2) in 1990 to 27·2 per 100 000 people (23·6 to 29·7) in 2020 (table 2). The global YLDs-to-YLLs ratio was 19·3 (15·3 to 24·8). Finally, the global age-standardised rate of DALYs was 539·0 (380·0 to 744·0) per 100 000 people, with the highest rate in high-income North America (984 [684 to 1300]).

**Table 2:** Number and age-standardised rate of deaths and YLLs in 2020 for other musculoskeletal disorders, and percentage change from 1990, by GBD region and super-region

		**Number of deaths**	**Percentage change in number of deaths from 1990 to 2020**	**Age-standardised rate of deaths per 100 000 people**	**Percentage change in age-standardised rate of deaths per 100 000 people from 1990 to 2020**	**Number of YLLs**	**Percentage change in number of YLLs from 1990 to 2020**	**Age-standardised rate of YLLs per 100 000 people**	**Percentage change in age-standardised rate of YLLs per 100 000 people from 1990 to 2020**
**Global**		**83 100 (73 600 to 91 600)**	**119·0 (102·0 to 136·0)**	**1·0 (0·9 to 1·1)**	**5·5 (−1·9 to 13·6)**	**2 210 000 (1 920 000 to 2 420 000)**	**61·0 (44·4 to 75·1)**	**27·2 (23·6 to 29·7)**	**−5·9 (−15·4 to 1·9)**
**Central Europe, eastern Europe, and central Asia**		**4820 (4 420 to 5 170)**	**145·6 (127·5 to 165·4)**	**0·8 (0·7 to 0·9)**	**79·1 (67·3 to 93·1)**	**109 000 (102 000 to 120 000)**	**52·9 (41·7 to 65·6)**	**20·4 (19·1 to 22·5)**	**25·7 (16·3 to 36·8)**
	Central Asia	320 (284 to 406)	1060·0 (758·0 to 1310·0)	0·6 (0·5 to 0·6)	846·0 (605·0 to 1030·0)	10 200 (8890 to 14 300)	886·0 (667·0 to 1230·0)	12·6 (11·2 to 16·5)	648·0 (473·0 to 867·0)
	Central Europe	929 (833 to 1030)	7·3 (−3·9 to 17·6)	0·5 (0·4 to 0·5)	−26·7 (−34·5 to −19·2)	22 700 (20 200 to 26 700)	−22·3 (−31·2 to −12·4)	13·4 (11·9 to 16·2)	−38·6 (−46·1 to −29·0)
	Eastern Europe	3570 (3230 to 3850)	234·0 (203·0 to 266·0)	1·1 (1·0 to 1·2)	154·0 (132·0 to 177·0)	76 500 (70 200 to 82 800)	85·2 (70·7 to 101·0)	27·2 (25·0 to 29·3)	58·1 (45·8 to 71·2)
**High income**		**25 100 (21 800 to 26 900)**	**75·1 (63·7 to 82·9)**	**1·1 (1·0 to 1·2)**	**−12·4 (−15·9 to −8·7)**	**454 000 (416 000 to 476 000)**	**28·1 (22·2 to 33·5)**	**26·2 (24·6 to 27·4)**	**−21·6 (−23·8– to 17·1)**
	Australasia	871 (745 to 947)	178·0 (148·0 to 203·0)	1·6 (1·4 to 1·7)	12·6 (1·9 to 21·6)	14 300 (12 900 to 15 500)	90·0 (71·4 to 105·0)	31·5 (29·0 to 34·1)	−7·1 (−15·7 to −0·1)
	High-income Asia Pacific	4210 (3460 to 4750)	124·0 (92·8 to 148·0)	0·9 (0·7 to 1·0)	−12·5 (−20·7 to −4·2)	73 500 (63 100 to 80 800)	19·5 (6·6 to 31·1)	21·0 (18·9 to 22·9)	−35·9 (−40·8 to −30·3)
	High-income North America	7160 (6350 to 7650)	43·9 (37·1 to 49·0)	1·2 (1·1 to 1·3)	−19·1 (−22·0 to −16·6)	166 000 (156 000 to 174 000)	15·6 (11·8 to 19·4)	33·1 (31·6 to 34·5)	−26·6 (−28·8 to −24·5)
	Southern Latin America	784 (718 to 843)	69·0 (56·4 to 85·1)	1·0 (0·9 to 1·0)	−8·4 (−15·2 to 0·4)	22 200 (20 700 to 23 600)	31·2 (21·6 to 43·6)	29·1 (27·3 to 31·0)	−18·1 (−24·1 to −10·3)
	Western Europe	12 100 (10 400 to 13 100)	80·3 (67·5 to 89·7)	1·1 (1·0 to 1·2)	−6·6 (−11·6 to −0·3)	178 000 (162 000 to 190 000)	42·4 (33·9 to 53·5)	21·5 (20·0 to 23·8)	−12·5 (−16·4 to −1·6)
**Latin America and Caribbean**		**8040 (7 400 to 8 690)**	**172·7 (153·7 to 192·8)**	**1·3 (1·2 to 1·4)**	**26·6 (17·4 to 36·1)**	**298 000 (277 000 to 324 000)**	**112·7 (95·5 to 130·2)**	**48·4 (45·0 to 52·6)**	**21·7 (12·1 to 31·7)**
	Andean Latin America	456 (383 to 560)	250·0 (183·0 to 343·0)	0·8 (0·6 to 0·9)	52·2 (19·4 to 91·3)	15 500 (12 800 to 20 400)	155·0 (104·0 to 233·0)	24·4 (20·1 to 31·8)	37·3 (10·9 to 81·4)
	Caribbean	802 (678 to 974)	118·0 (89·9 to 153·0)	1·6 (1·3 to 1·9)	14·4 (0·2 to 30·4)	27 600 (22 200 to 36 400)	86·7 (57·6 to 121·0)	55·2 (44·4 to 73·4)	20·8 (2·8 to 43·5)
	Central Latin America	3840 (3250 to 4270)	193·0 (140·0 to 228·0)	1·5 (1·3 to 1·7)	38·0 (13·9 to 53·6)	149 000 (126 000 to 167 000)	128·0 (86·2 to 155·0)	57·4 (48·8 to 64·3)	28·8 (5·5 to 44·2)
	Tropical Latin America	2940 (2780 to 3190)	158·0 (143·0 to 175·0)	1·2 (1·1 to 1·3)	18·5 (13·1 to 27·0)	106 000 (102 000 to 117 000)	96·6 (86·7 to 111·0)	43·8 (41·9 to 47·8)	13·6 (8·1 to 21·9)
**North Africa and Middle East**		**3320 (2680 to 4000)**	**153·0 (107·0 to 201·0)**	**0·7 (0·6 to 0·9)**	**12·8 (−6·3 to 39·0)**	**127 000 (104 000 to 155 000)**	**106·0 (62·9 to 146·0)**	**22·0 (17·8 to 26·6)**	**3·1 (−16·0 to 23·4)**
**South Asia**		**21 700 (16 500 to 26 300)**	**232·0 (194·0 to 277·0)**	**1·8 (1·4 to 2·2)**	**14·5 (−0·9 to 28·3)**	**471 000 (364 000 to 558 000)**	**139·0 (80·5 to 221·0)**	**33·4 (25·8 to 40·1)**	**8·3 (−5·1 to 21·6)**
**Southeast Asia, east Asia, and Oceania**		**15 300 (12 800 to 18 400)**	**70·5 (33·7 to 110·0)**	**0·6 (0·5 to 0·8)**	**8·5 (−15·0 to 33·4)**	**540 000 (451 000 to 640 000)**	**12·7 (−9·4 to 40·9)**	**23·6 (19·5 to 27·9)**	**−14·0 (−31·4 to 6·9)**
	East Asia	10 300 (8430 to 13 100)	63·1 (23·8 to 116·0)	0·6 (0·5 to 0·7)	2·5 (−22·0 to 35·2)	331 000 (276 000 to 411 000)	−1·6 (−25·2 to 32·1)	20·5 (17·1 to 25·2)	−23·0 (−41·4 to 3·4)
	Oceania	38·5 (16·1 to 58)	139·0 (80·5 to 221·0)	0·4 (0·2 to 0·5)	6·8 (−15·3 to 33·5)	2140 (805 to 3340)	137·0 (76·8 to 232·0)	16·1 (6·5 to 24·4)	9·7 (−17·3 to 47·2)
	Southeast Asia	4950 (3970 to 5640)	88·0 (51·6 to 119·0)	16·1 (6·5 to 24·4)	11·1 (−6·6 to 25·6)	206 000 (167 000 to 236 000)	46·3 (17·1 to 75·1)	29·7 (24·0 to 34·0)	−3·7 (−23·2 to 13·5)
**Sub-Saharan Africa**		**4810 (3 490 to 6 010)**	**149·0 (112·9 to 195·4)**	**0·8 (0·6 to 1·1)**	**13·8 (−1·9 to 34·5)**	**211 000 (150 000 to 262 000)**	**141·7 (98·6 to 192·8)**	**25·2 (18·3 to 31·5)**	**6·9 (−9·1 to 26·3)**
	Central sub-Saharan Africa	619 (311 to 971)	124·0 (68·0 to 193·0)	1·0 (0·5 to 1·6)	−7·7 (−32·7 to 21·5)	26 100 (13 100 to 40 600)	114·0 (59·2 to 189·0)	28·4 (14·3 to 44·6)	−11·6 (−33·8 to 15·3)
	Eastern sub-Saharan Africa	1080 (638 to 1760)	90·4 (43·4 to 142·0)	0·5 (0·3 to 0·9)	−11·5 (−28·9 to 3·8)	46 200 (27 500 to 73 300)	77·7 (27·5 to 144·0)	15·6 (9·2 to 25·3)	−19·2 (−39·2 to 1·3)
	Southern sub-Saharan Africa	822 (679 to 953)	137·0 (93·0 to 194·0)	1·4 (1·1 to 1·6)	36·1 (13·6 to 68·4)	29 700 (24 400 to 35 100)	90·4 (52·5 to 133·0)	41·4 (34·1 to 48·2)	14·5 (−7·0 to 41·1)
	Western sub-Saharan Africa	2290 (1640 to 2810)	209·0 (145·0 to 300·0)	0·9 (0·7 to 1·1)	26·6 (3·1 to 63·8)	109 000 (74 100 to 138 000)	225·1 (152·0 to 318·9)	28·3 (20·5 to 34·8)	29·2 (1·7 to 67·4)

Data in parentheses are 95% uncertainty intervals. North Africa and Middle East and South Asia are both super-regions and regions. The sum of regional deaths and regional YLLs might not exactly match the totals for the super-regions because of rounding. YLLs=years of life lost.

**Figure 3:**
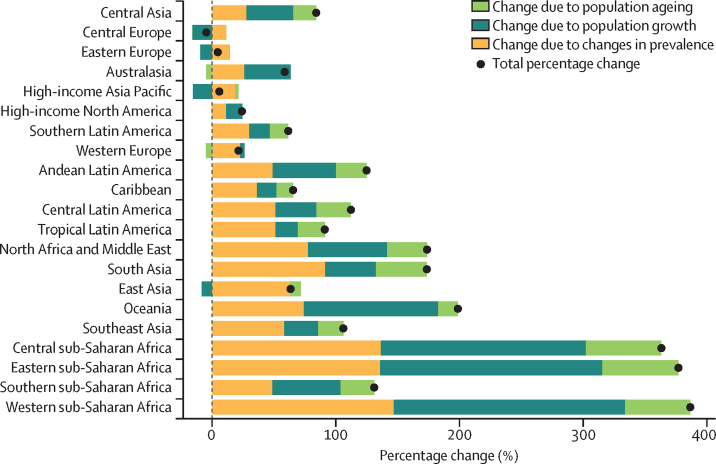
Decomposition of forecasted change in the number of prevalent cases of other musculoskeletal disorders by region from 2020 to 2050

Based on forecasted changes in population, in 2050 an estimated 1060 million (95% UI 964–1170) people globally will have other musculoskeletal disorders, an increase from 2020 to 2050 of 115% (107–124; appendix pp 41, 43). A decomposition analysis both globally and by region shows the relative contribution of population growth, population ageing, and changes in prevalence to the forecasted increase in cases (figure 3). Population growth is the largest contributor to the projected prevalence changes in central, eastern, western, and southern regions of sub-Saharan Africa. Change in age-standardised prevalence also contributed to the projected case increase in these areas. A small decline in prevalence is forecast for central Europe owing to declines in population growth. The prevalence in eastern Europe and high-income Asia Pacific is projected to remain stable, predominantly because of declines in population growth.

## Discussion

The burden of the other musculoskeletal disorders category in the GBD was estimated to be large but inconclusive. In 2020, other musculoskeletal disorders ranked as the sixth-largest cause of YLDs and the 19th-largest cause of DALYs, even after subtracting the long-term disability from fractures and dislocations estimated under injury causes in the GBD. The global prevalence of other musculoskeletal disorders was higher in females than in males and overall increased with age, peaking at 60–69 years. Owing to population growth, ageing, and change in prevalence, we forecast an increase in cases of other musculoskeletal disorders from 494 million in 2020 to 1060 million in 2050.

Few other attempts have been made to quantify some of the conditions that form part of the other musculoskeletal disorders category. Meta-analyses of the prevalence of conditions such as systemic lupus erythematosus,^12^, ^13^ psoriatic arthritis,^14^ spondyloarthritis,^15^ and ankylosing spondylitis have been conducted.^16^ These studies show wide variations in prevalence, study design, and case definition^12^, ^13^, ^14^, ^15^, ^16^ and generally focus on specific regions rather than global estimates.^14^ However, these individual studies and meta-analyses show the possibility of explicitly modelling conditions that are currently grouped under other musculoskeletal disorders, and the ability to and utility in doing so.

Recognition of the burden of muscle and joint infections is increasing,^17^ and this burden needs to be included in estimates to capture the true burden of other musculoskeletal disorders. Collaborating on study design and case definition would be the first step in attempting to better quantify these conditions. Explicit modelling of individual conditions would also provide opportunities to assess risk factors, which is not practical for a heterogeneous residual category. More generally, improving health care for ageing populations—in terms of non-communicable disease prevention, rehabilitation, and drug efficacy and safety—would help to reduce the burden of musculoskeletal conditions along with other conditions such as cardiovascular disease.

Owing to the heterogeneous mix of conditions included in the other musculoskeletal disorders category, discussing the implications for health policy and service provision is difficult. The large YLD burden, despite all uncertainty, suggests a large demand for therapeutic and rehabilitative services; this demand is supported by insurance claims data, which indicate a large number of health service visits. To better assess the implications on policy, splitting this large residual category into a number of separate causes, each with a sizeable expected burden, is imperative. However, a further challenge is that some of the ICD codes (142 of the 5940 ICD codes included in other musculoskeletal disorders) are themselves classified as other, undefined, or unspecified. This lack of specificity creates the resource-intensive tasks of identifying data sources through systematic reviews of published and grey literature, developing models to estimate prevalence and severity distributions, and possibly creating new health states and disability weights if the current set of GBD health states are deemed inadequate to describe the ensuing disabling consequences of these conditions. Improvement in ICD codes related to these diseases to reduce the number of conditions classified as other, undefined, or unspecified could be of benefit. Another future consideration is the emergence of post-COVID-19 condition—effects of COVID-19 that remain months after patients initially become ill. Many of the symptom profiles of post-COVID-19 condition are characterised by musculoskeletal symptoms coupled with fatigue and loss of mobility. For more specific other musculoskeletal disorders, identifying potentially modifiable risk factors and treating these disorders as symptoms (in a similar manner to low back pain or headache) might be easier than retaining an amorphous residual category for them. Furthermore, as many musculoskeletal disorders are accompanied by chronic pain, forecasts could help policy makers to allocate sufficient resources to closely aligned conditions such as opioid dependence, which disproportionately affect people with chronic pain.

The global musculoskeletal disorders community has specifically highlighted the need to capture more country-level data for musculoskeletal disorders, as evidenced by the development of a standardised global survey module to specifically measure the population prevalence of musculoskeletal pain.^18^ This need parallels that for better disaggregation and more complete global data on the individual conditions comprising the other musculoskeletal disorders category, to provide more useful information to clinicians and policy makers.^3^, ^19^, ^20^ Until progress is made to address this need, the highly heterogenous nature of the other musculoskeletal disorders category makes policy recommendations difficult, other than highlighting the effect of these conditions on the population.

Data sources currently available to estimate the prevalence of other musculoskeletal disorders are sparse, which can distort the results and could also be an indication of the interest in these disorders on a global level. Given the limitations in these data, we are also unable to clearly define the most common other musculoskeletal disorders or to easily assess geographical variation. A considerable burden that also remains unaccounted for in the GBD estimates is that among the paediatric population, as it was assumed that other musculoskeletal disorders are not present in children younger than 10 years. Children can have a range of musculoskeletal disorders that do not appear in the current estimates.^21^ Hoy and colleagues^20^ also noted this lack of data, and it has yet to be adequately addressed.

Selecting appropriate predictive covariates is challenging for a heterogeneous category such as other musculoskeletal disorders and makes estimates of disease burden more difficult. In this case we have followed standard GBD forecasting methods, which include only SDI—even though SDI itself changes with time and its temporal trends could introduce corresponding trends to the model when it is used as a covariate. Methods could be improved in future by including other relevant predictive covariates, such as BMI. If more specific conditions are separated from the large residual category of other musculoskeletal disorders in the future, targeted searches for covariates and risk factors will be easier. A further limitation of the forecasting model is that the forecasts do not account for the effect of the COVID-19 pandemic on the prevalence and burden of other musculoskeletal disorders and the potential for decreased diagnosis and treatment.

A limited number of surveys, largely those following the COPCORD study design, provide prevalence estimates for the overall amount of musculoskeletal symptoms (largely pain, stiffness, and loss of mobility) and the specific musculoskeletal conditions included in the GBD, allowing an estimate of the prevalence of other musculoskeletal disorders by subtraction. This approach assumes no overlap between other musculoskeletal disorders and any of the specific musculoskeletal disorders in the GBD (osteoarthritis, rheumatoid arthritis, gout, low back pain, and neck pain). As these conditions are highly prevalent, considerable overlap and, possibly, dependent comorbidity between musculoskeletal disorders are likely. Such overlap means that our current estimates of disease burden are potentially too low rather than too high. An exhaustive set of ICD codes could be more comprehensive than surveys. The use of insurance claims data from the USA and Poland is another source of underestimation, because not all people with a condition classified under other musculoskeletal disorders will be seeking health care, particularly those with chronic symptoms over many years. A further limitation is that, owing to timeline conflicts, the estimates reported here are based on 100 model draws, whereas 1000 draws would be preferable.

Other musculoskeletal disorders are a heterogeneous group of musculoskeletal conditions that are not captured elsewhere in disease-specific categories. As these conditions are such a large source of disability, they should not be overlooked when determining policy for musculoskeletal health. Further population-based collection and delineation of disease subtypes within the category of other musculoskeletal disorders is warranted to better inform a health policy response to these highly prevalent conditions. However, recommendations do exist (eg, from the Global Alliance for Musculoskeletal Health) to strengthen the ability of health systems to manage and improve musculoskeletal health and are applicable to all musculoskeletal disorders, regardless of the specific condition. The large disease burden from this diverse category of musculoskeletal disorders emphasises the need to address musculoskeletal conditions in general.

## Data sharing

The findings of this study are supported by data available in public online repositories, data publicly available upon request to the data provider, and data not publicly available owing to restrictions by the data provider. Non-publicly available data were used under license for the current study but may be available from the authors upon reasonable request and with permission of the data provider. Data sources used in this analysis are listed in the appendix (pp 9–12).

## Declaration of interests

A M Briggs reports grants from the Bone and Joint Decade Foundation, consulting fees from WHO, and support for attending meetings and/or travel from WHO, all outside the submitted work. K Krishan reports non-financial support from the UGC Centre of Advanced Study, CAS II, Department of Anthropology, Panjab University (Chandigarh, India), outside the submitted work. A J Mathew reports research grants from Novartis and payment or honoraria for lectures, presentations, speakers bureaus, manuscript writing, or educational events from Cipla and Novartis, all outside the submitted work. H Slater reports grants from the Australian government (Department of Health), the Medical Research Future Fund (National Health and Medical Research Council), the Western Australian government (Department of Health), the Bone and Joint Decade Foundation (Lund, Sweden), Curtin University (Perth, WA, Australia), and the Canadian Memorial Chiropractic College (Toronto, ON, Canada); and support for attending meetings and travel from the Australian Pain Society; all outside the submitted work.
