# Supplementary material for: Global, regional, and national burden of other musculoskeletal disorders, 1990–2020, and projections to 2050: a systematic analysis of the Global Burden of Disease Study 2021
Source: Lancet Rheumatol. 2023 Oct 23;5(11):e670–82. doi: 10.1016/S2665-9913(23)00232-1 (PMC10620749; doi:10.1016/S2665-9913(23)00232-1)

THE LANCET
Rheumatology

Supplementary appendix

This appendix formed part of the original submission and has been peer reviewed. We post it as supplied by the authors.

Supplement to: GBD 2021 Other Musculoskeletal Disorders Collaborators. Global, regional, and national burden of other musculoskeletal disorders, 1990–2020, and projections to 2050: a systematic analysis of the Global Burden of Disease Study 2021. *Lancet Rheumatol* 2023; **5:** e670–82.

Global, regional, and national burden of other musculoskeletal disorders, 1990–2020, and projections to 2050: a systematic analysis of the Global Burden of Disease Study 2021

CONTENTS

	Page
**Section 1. Statement of GATHER Compliance**	**1**
**Section 2. Disability weights**	**1**
**Section 3. Severity distribution**	**1-2**
**Section 4. Author information**	**2-7**
GBD 2021 Other Musculoskeletal Disorders Collaborators	2
Affiliations	3-6
Authors' contributions	6-7
**Section 5. Methods**	**8-14**
Supplemental Table 1: ICD codes	8
Supplemental Table 2: Data sources	9-12
Supplemental Table 3: Summary of covariates used in the other MSK DisMod-MR meta-regression model	12
Supplemental Table 4: Severity distribution and associated disability weights (DW)	13-14
Supplemental Table 5: Validation experiment for forecasting method	14
**Section 6. Results**	**15-43**
Supplemental Table 6: Counts and age-standardised rates of prevalence and death in 2020 for other MSK disorders, by region and by country	15-24
Supplemental Table 7: Counts and age-standardised rates of YLDs, YLLs, and DALYs in 2020 for other MSK disorders, by region and by country	25-40
Supplemental Table 8: Forecast of other MSK age-standardised prevalence and total cases globally and by region, from 2020 to 2050	41
Supplemental Figure 1: Unique source-years of non-fatal data for other MSK	42
Supplemental Figure 2: Unique source-years of cause of death data for other MSK	42
Supplemental Figure 3: Global forecast of other MSK conditions to 2050, males and females	43
Supplemental Figure 4: Forecast of other MSK by region to 2050	43

Section 1. Statement of GATHER Compliance

This study complies with the Guidelines for Accurate and Transparent Health Estimates Reporting (GATHER) recommendations.[2]

Section 2. Disability weights

The basis of the GBD disability weight survey assessments are lay descriptions of health states highlighting major functional consequences and symptoms. The lay descriptions and disability weights for other MSK severity levels are shown below. They include the three levels of health states that are used for osteoarthritis and rheumatoid arthritis.

Section 3. Severity distribution

The severity distributions are derived from an analysis of the Medical Expenditure Panel Surveys (MEPS) in the USA. MEPS is an overlapping continuous panel survey of the USA non-institutionalised population whose primary purpose is to collect information on the use and cost of health care. Panels are two years long and consist of five rounds, which are conducted every five to six months. A new panel begins annually, while the last panel is in its second year

(http://www.meps.ahrq.gov/survey_comp/hc_data_collection.jsp). Each panel typically contains about 30,000 to 35,000 individual respondents. MEPS was initiated in 1996 but only began collecting health status data in the form of 12-Item Short Form Survey (SF-12) responses in 2000. For GBD 2021 we used data from 2000–2014. Respondents self-administer the SF-12 twice per panel, at rounds two and four, typically about a year apart. Only adults 18 years and older completed the SF-12. MEPS also usually collects information on diagnoses based on self-report of reasons for encounters with health services. In addition, diagnoses are derived through additional questions on “problems that bother you” or conditions that led to “disability days,” ie, days out of role due to illness. Professional coders translate the verbatim text into three-digit ICD-9 codes. The main reason for other MSK being measured in MEPS relates to health care contact. In order to convert SF-12 values into a scale comparable with that used by the GBD disability weights, small studies on convenience samples were conducted asking respondents to fill in SF-12 to reflect 62 lay descriptions of diverse severity that were used to derive the GBD disability weights. From these responses a relationship between SF-12 summary score and the GBD DWs was derived, and from it the contribution from other MSK conditions was computed by adjusting it for comorbid conditions.

Section 4. Author information

GBD 2021 Other Musculoskeletal Disorders Collaborators

Tiffany K Gill*, Manasi Murthy Mittinty*, Lyn M March, Jaimie D Steinmetz, Garland T Culbreth, Marita Cross, Jacek A Kopec, Anthony D Woolf, Lydia M Haile, Hailey Hagins, Kanyin Liane Ong, Deborah R Kopansky-Giles, Karsten E Dreinhoefer, Neil Betteridge, Mohammadreza Abbasian, Mitra Abbasifard, krishna Abedi, Miracle Ayomikun Adesina, Janardhana P Aithala, Mostafa Akbarzadeh-Khiavi, Yazan Al Thaher, Tariq A Alalwan, Hosam Alzahrani, Sohrab Amiri, Benny Antony, Jalal Arabloo, Aleksandr Y Aravkin, Ashokan Arumugam, Krishna K Aryal, Seyyed Shamsadin Athari, Alok Atreya, Soroush Baghdadi, Mainak Bardhan, Lope H Barrero, Lindsay M Bearne, Alehegn Bekele Bekele, Isabela M Bensenor, Pankaj Bhardwaj, Rajbir Bhatti, Ali Bijani, Theresa Bordianu, Souad Bouaoud, Andrew M Briggs, Huzaifa Ahmad Cheema, Steffan Wittrup McPhee Christensen, Isaac Sunday Chukwu, Benjamin Clarsen, Xiaochen Dai, Katie de Luca, Belay Desye, Meghnath Dhimal, Thanh Chi Do, Adeniyi Francis Fagbamigbe, Siamak Farokh Forghani, Nuno Ferreira, Balasankar Ganesan, Mesfin Gebrehiwot, Ahmad Ghashghaee, Simon Matthew Graham, Netanja I Harlianto, Jan Hartvigsen, Ahmed I Hasaballah, Mohammad Hasanian, Mohammed Bheser Hassen, Simon I Hay, Mohammad Heidari, Alexander Kevin Hsiao, Irena M Ilic, Mohammad Jokar, Himanshu Khajuria, Md Jobair Khan, Praval Khanal, Sorour Khateri, Ali Kiadaliri, Min Seo Kim, Adnan Kisa, Ali-Asghar Kolahi, Kewal Krishan, Vijay Krishnamoorthy, Iván Landires, Bagher Larijani, Thao Thi Thu Le, Yo Han Lee, Stephen S Lim, Justin Lo, Seyedeh Panid Madani, Jeadran N Malagón-Rojas, Iram Malik, Hamid Reza Marateb, Ashish J Mathew, Tuomo J Meretoja, Mohamed Kamal Mesregah, Tomislav Mestrovic, Alireza Mirahmadi, Awoke Misganaw, Sadra Mohaghegh, Ali H Mokdad, Kaveh Momenzadeh, Sara Momtazmanesh, Lorenzo Monasta, Mohammad Ali Moni, Yousef Moradi, Ebrahim Mostafavi, Jibran Sualeh Muhammad, Christopher J L Murray, Sathish Muthu, Shumaila Nargus, Hasan Nassereldine, Subas Neupane, Robina Khan Niazi, In- Hwan Oh, Hassan Okati-Aliabad, Abderrahim Oulhaj, Kevin Pacheco-Barrios, Seoyeon Park, Jay Patel, Shrikant Pawar, Paolo Pedersini, Mario F P Peres, Ionela-Roxana Petcu, Fanny Emily Petermann-Rocha, Mohsen Poursadeqiyan, Ibrahim Qattea, Maryam Faiz Qureshi, Quinn Rafferty, Shahram Rahimi- Dehgolan, Mosiur Rahman, Shakthi Kumaran Ramasamy, Vahid Rashedi, Elrashdy Moustafa Mohamed Redwan, Daniel Cury Ribeiro, Leonardo Roever, Azam Safary, Dominic Sagoe, Fatemeh Saheb Sharif- Askari, Amirhossein Sahebkar, Sana Salehi, Amir Shafaat, Saeed Shahabi, Saurab Sharma, Bereket Beyene Shashamo, Rahman Shiri, Ambrish Singh, Helen Slater, Amanda E Smith, Dev Ram Sunuwar, Mohammad Tabish, Samar Tharwat, Irfan Ullah, Sahel Valadan Tahbaz, Tommi Juhani Vasankari, Jorge Hugo Villafañe, Stein Emil Vollset, Taweewat Wiangkham, Naohiro Yonemoto, Yuyi You, Iman Zare, Peng Zheng, Theo Vos[#], and Peter M Brooks[#].
*Joint First Authors
[#]Joint Senior Author

Affiliations

Adelaide Medical School (T K Gill PhD), University of Adelaide, Adelaide, SA, Australia; College of Medicine and Public Health (M M Mittinty PhD), Flinders University, Bedford Park, SA, Australia; Faculty of Medicine and Health (Prof L M March PhD, M Cross PhD) and Save Sight Institute (Y You PhD), University of Sydney, Sydney, NSW, Australia; Department of Rheumatology (Prof L M March PhD), Royal North Shore Hospital, St Leonards, NSW, Australia; Institute for Health Metrics and Evaluation (J D Steinmetz PhD, G T Culbreth PhD, L M Haile BA, H Hagins MSPH, K L Ong PhD, A Y Aravkin PhD, T Bordianu BS, X Dai PhD, M Hassen BSc, Prof S I Hay FMedSci, Prof S S Lim PhD, J Lo BA, T Mestrovic PhD, A H Mokdad PhD, Prof C J L Murray DPhil, H Nassereldine MD, Q Rafferty BA, A E Smith MPA, Prof S Vollset DrPH, P Zheng PhD, Prof T Vos PhD), Department of Applied Mathematics (A Y Aravkin PhD), Department of Health Metrics Sciences, School of Medicine (A Y Aravkin PhD, X Dai PhD, Prof S I Hay FMedSci, Prof S S Lim PhD, A Misganaw PhD, A H Mokdad PhD, Prof C J L Murray DPhil, Prof S Vollset DrPH, P Zheng PhD, Prof T Vos PhD), and Department of Anesthesiology and Pain Medicine (V Krishnamoorthy MD), University of Washington, Seattle, WA, USA; Global Alliance for Musculoskeletal Health, Sydney, NSW, Australia (Prof L March PhD, M Cross PhD, Prof A D Woolf MBBS, Prof K E Dreinhoefer Staatsexamen, Prof D Kopansky-Giles MSc, N Betteridge MPhil, Prof A M Briggs PhD); School of Population and Public Health (J A Kopec PhD), University of British Columbia, Vancouver, BC, Canada; Arthritis Research Canada, Richmond, BC, Canada (J A Kopec PhD); Bone and Joint Research Group (Prof A D Woolf MBBS), Royal Cornwall Hospital, Truro, UK; Department of Family and Community Medicine (Prof D R Kopansky-Giles MSc), University of Toronto, Toronto, ON, Canada; Department of Research and Innovation (Prof D R Kopansky-Giles MSc), Canadian Memorial Chiropractic College, Toronto, ON, Canada; Center of Musculoskeletal Surgery (Prof K E Dreinhoefer Staatsexamen), Charite University Medical Center Berlin, Berlin, Germany; Department of Orthopedic Surgery (M Abbasian MD, K Momenzadeh MD) and Physical Medicine and Rehabilitation (K Pacheco-Barrios MD), Harvard University, Boston, MA, USA; Department of Orthopaedic Surgery (M Abbasian MD), Social Determinants of Health Research Center (A Kolahi MD), Department of Orthopedics (A Mirahmadi MD), and Department of Oral and Maxillofacial Surgery (S Mohaghegh DDS), Shahid Beheshti University of Medical Sciences, Tehran, Iran; Department of Internal Medicine (M Abbasifard MD) and Clinical Research Development Unit (M Abbasifard MD), Rafsanjan University of Medical Sciences, Rafsanjan, Iran; Department of Neurosurgery (K Abedi MD), Keck School of Medicine (K Abedi MD), and Mark and Mary Stevens Neuroimaging and Informatics Institute (S Salehi MD), University of Southern California, Los Angeles, CA, USA; Slum and Rural Health Initiative Research Academy (M A Adesina BPT), Slum and Rural Health Initiative, Ibadan, Nigeria; Department of Physiotherapy (M A Adesina BPT) and Department of Epidemiology and Medical Statistics (A F Fagbamigbe PhD), University of Ibadan, Ibadan, Nigeria; Orthopedic Department (Prof J P Aithala DNB), Yenepoya Medical College, Mangalore, India; Liver and Gastrointestinal Diseases Research Center (M Akbarzadeh-Khiavi PhD) and Connective Tissue Diseases Research Center (A Safary PhD), Tabriz University of Medical Sciences, Tabriz, Iran; Faculty of Pharmacy (Y Al Thaher PhD), Philadelphia University, Amman, Jordan; School of Pharmacy (Y Al Thaher PhD), Cardiff University, Cardiff, UK; Department of Biology (T A Alalwan PhD), University of Bahrain, Sakhir, Bahrain; Department of Physiotherapy (H Alzahrani PhD), Taif University, Taif, Saudi Arabia; Quran and Hadith Research Center (S Amiri PhD), Baqiyatallah University of Medical Sciences, Tehran, Iran; Menzies Institute for Medical Research (B Antony PhD, A Singh MTech), University of Tasmania, Hobart, TAS, Australia; Health Management and Economics Research Center (J Arabloo PhD), Department of Plastic and Reconstructive Surgery (S Farokh Forghani MD), and Burn Research Center (S Farokh Forghani MD), Iran University of Medical Sciences, Tehran, Iran; Department of Physiotherapy (A Arumugam PhD), Department of Basic Medical Sciences (J Muhammad PhD), and Sharjah Institute of Medical Sciences (F Saheb Sharif-Askari PhD), University of Sharjah, Sharjah, United Arab Emirates; Community Medicine and Rehabilitation, Physiotherapy Section (A Arumugam PhD), Umea University, Umea, Sweden; Monitoring Evaluation and Operational Research Project (K K Aryal PhD), Abt Associates Nepal, Lalitpur, Nepal; Department of Immunology (S Athari PhD), Zanjan University of Medical Sciences, Zanjan, Iran; Department of Forensic Medicine (A Atreya MD), Lumbini Medical College, Palpa, Nepal; Division of Orthopaedics (S Baghdadi MD), Children's Hospital of Philadelphia, Philadelphia, PA, USA;

Department of Molecular Microbiology and Bacteriology (M Bardhan MD), National Institute of Cholera and Enteric Diseases, Kolkata, India; Molecular Microbiology (M Bardhan MD), Indian Council of Medical Research, New Delhi, India; Department of Industrial Engineering (Prof L H Barrero DSc), Pontifical Javeriana University, Bogota, Colombia; Population Health Research Institute (Prof L M Bearne PhD), University of London, London, UK; Centre for Engagement and Dissemination (Prof L M Bearne PhD), National Institute for Health Research, Twickenham, UK; Department of Medical Anatomy (A B Bekele MSc) and Department of Nursing (B B Shashamo MSc), Arba Minch University, Arba Minch, Ethiopia; Department of Internal Medicine (I M Bensenor PhD) and Department of Psychiatry (Prof M F P Peres MD), University of Sao Paulo, Sao Paulo, Brazil; Department of Community Medicine and Family Medicine (P Bhardwaj MD) and School of Public Health (P Bhardwaj MD), All India Institute of Medical Sciences, Jodhpur, India; Department of Pharmaceutical Sciences (R Bhatti PhD), Guru Nanak Dev University, Amritsar, India; Social Determinants of Health Research Center (A Bijani PhD), Babol University of Medical Sciences, Babol, Iran; Department of Medicine (Prof S Bouaoud MD), University Ferhat Abbas of Setif, Setif, Algeria; Department of Epidemiology and Preventive Medicine (Prof S Bouaoud MD), University Hospital Saadna Abdenour, Setif, Algeria; School of Physiotherapy and Exercise Science (Prof A M Briggs PhD, Prof H Slater PhD), Curtin University, Perth, WA, Australia; Department of Medicine (H A Cheema MB) and Department of Community Medicine and Public Health (H A Cheema MB), King Edward Medical University, Lahore, Pakistan; Department of Health Science and Technology (S W M Christensen PhD), Aalborg University, Aalborg, Denmark; Department of Physiotherapy (S W M Christensen PhD), University College of Northern Denmark, Aalborg, Denmark; Department of Paediatric Surgery (I S Chukwu BMedSc), Federal Medical Centre, Umuahia, Nigeria; Department of Disease Burden (B Clarsen PhD), Norwegian Institute of Public Health, Bergen, Norway; Department of Sports Medicine (B Clarsen PhD), Norwegian School of Sport Sciences, Oslo, Norway; Discipline of Chiropractic (K de Luca PhD), CQ University, Brisbane, QLD, Australia; Department of Public Health (B Desye MSc), Adigrat University, Adigrat, Ethiopia; Health Research Section (M Dhimal PhD), Nepal Health Research Council, Kathmandu, Nepal; Department of Medicine (T C Do MD), Pham Ngoc Thach University of Medicine, Ho Chi Minh City, Viet Nam; Institute of Applied Health Sciences (A F Fagbamigbe PhD), University of Aberdeen, Aberdeen, UK; Department of Social Sciences (N Ferreira PhD), University of Nicosia, Nicosia, Cyprus; School of Global Health (B Ganesan PhD), Institute of Health and Management, Melbourne, VIC, Australia; Department of Occupational Therapy (B Ganesan PhD), Mahatma Gandhi Occupational Therapy College, Jaipur, India; Department of Environmental Health (M Gebrehiwot DSc), Dessie, Ethiopia; School of Public Health (A Ghashghaee BSc), Qazvin University of Medical Sciences, Qazvin, Iran; Nuffield Department of Orthopaedics (S M Graham PhD), Oxford University, Oxford, UK; Liverpool Orthopaedic and Trauma Service (S M Graham PhD), University of Liverpool, Liverpool, UK; Faculty of Medicine (N I Harlianto BSc), Utrecht University, Utrecht, Netherlands; Department of Radiology (N I Harlianto BSc), University Medical Center Utrecht, Utrecht, Netherlands; Department of Sports Science and Clinical Biomechanics (Prof J Hartvigsen PhD), University of Southern Denmark, Odense, Denmark; Research Department (Prof J Hartvigsen PhD), Nordic Institute of Chiropractic and Clinical Biomechanics, Odense, Denmark; Department of Zoology and Entomology (A I Hasaballah PhD), Al Azhar University, Cairo, Egypt; Department of Radiology (M Hasanian MD), Arak University of Medical Sciences, Arak, Iran; National Data Management Center for Health (M Hassen BSc, A Misganaw PhD), Ethiopian Public Health Institute, Addis Ababa, Ethiopia; Community-Oriented Nursing Midwifery Research Center (M Heidari PhD), Shahrekord University of Medical Sciences, Shahrekord, Iran; Outpatient Rehabilitation (A K Hsiao DPT), Southcoast Health: Tobey Hospital, Wareham, MA, USA; Faculty of Medicine (I M Ilic PhD), University of Belgrade, Belgrade, Serbia; Zoonoses Research Center (M Jokar DVM) and Department of Microbiology (S Valadan Tahbaz PhD), Islamic Azad University, Tehran, Iran; Department of Clinical Sciences (M Jokar DVM), Jahrom University of Medical Sciences, Jahrom, Iran; Amity Institute of Forensic Sciences (H Khajuria PhD), Amity University, Noida, India; Department of Rehabilitation Sciences (M Khan MPH), Hong Kong Polytechnic University, Hong Kong Special Administrative Region, China; Department of Medical Biochemistry (P Khanal PhD), Pokhara University, Kathmandu, Nepal; School of Medicine (S Khateri MD), Kurdistan University of Medical Sciences, Sanandaj, Iran; Clinical Epidemiology Unit (A Kiadaliri PhD), Lund University, Lund, Sweden; Department of Genomics and Digital Health (M Kim MD) and Samsung

Advanced Institute for Health Sciences and Technology, Seoul, South Korea; Public Health Center (M Kim MD), Ministry of Health and Welfare, Wando, South Korea; School of Health Sciences (Prof A Kisa PhD), Kristiania University College, Oslo, Norway; Department of International Health and Sustainable Development (Prof A Kisa PhD), Tulane University, New Orleans, LA, USA; Department of Anthropology (Prof K Krishan PhD), Panjab University, Chandigarh, India; Department of Anesthesiology (V Krishnamoorthy MD), Duke University, Durham, NC, USA; Unit of Genetics and Public Health (Prof I Landires MD), Institute of Medical Sciences, Las Tablas, Panama; Ministry of Health, Herrera, Panama (Prof I Landires MD); Endocrinology and Metabolism Research Institute (Prof B Larijani FACE), School of Medicine (S Momtazmanesh MD), Non-Communicable Diseases Research Center (S Momtazmanesh MD) and Physical Medicine and Rehabilitation (S Rahimi-Dehgolan MD), Tehran University of Medical Sciences, Tehran, Iran; University of Medicine and Pharmacy at Ho Chi Minh City, Ho Chi Minh City, Viet Nam (T T Le MD); Department of Preventive Medicine (Prof Y Lee PhD), Korea University, Seoul, South Korea; Department of Radiology (S Madani MD), Johns Hopkins University, Baltimore, MD, USA; Department of Public Health Research (J N Malagon-Rojas MSc), National Institute of Health, Bogota, Colombia; Faculty of Medicine (J N Malagon-Rojas MSc), El Bosque University, Bogota, Colombia; Material Science Programme (I Malik PhD), Indian Institute of Technology Kanpur, Kanpur, India; Biomedical Engineering Research Center (H Marateb PhD), Universidad Politecnica de Cataluna, Barcelona Tech, Barcelona, Spain; Department of Biomedical Engineering (H Marateb PhD, A Shafaat MS), University of Isfahan, Isfahan, Iran; Department of Clinical Immunology and Rheumatology (A J Mathew DM), Christian Medical College and Hospital, Vellore, India; Breast Surgery Unit (T J Meretoja MD), Helsinki University Hospital, Helsinki, Finland; University of Helsinki, Helsinki, Finland (T J Meretoja MD); Faculty of Medicine (M K Mesregah MD), Menoufia University, Shebin El-Kom, Egypt; University Centre Varazdin (T Mestrovic PhD), University North, Varazdin, Croatia; Clinical Epidemiology and Public Health Research Unit (L Monasta DSc), Burlo Garofolo Institute for Maternal and Child Health, Trieste, Italy; School of Health and Rehabilitation Sciences (M Moni PhD), The University of Queensland, Brisbane, QLD, Australia; Social Determinants of Health Research Center (Y Moradi PhD), Kurdistan University of Medical Sciences, Kurdistan, Iran; Department of Medicine (E Mostafavi PhD) and Stanford Cardiovascular Institute (E Mostafavi PhD), Stanford University, Palo Alto, CA, USA; Department of Orthopaedics (S Muthu MS), Government Medical College, Dindigul, Dindigul, India; Quality Appraisal Committee (S Muthu MS), Orthopaedic Research Group, Coimbatore, India; Department of Psychiatry (S Nargus PhD), Applied Biomedical Research Center (A Sahebkar PhD), and Biotechnology Research Center (A Sahebkar PhD), Mashhad University of Medical Sciences, Mashhad, Iran; University Institute of Public Health (S Nargus PhD), The University of Lahore, Lahore, Pakistan; Department of Health Sciences (S Neupane PhD), University of Tampere, Tampere, Finland; International Islamic University Islamabad, Islamabad, Pakistan (R K Niazi PhD); Department of Preventive Medicine (I Oh PhD), Kyung Hee University, Dongdaemun-gu, South Korea; Health Promotion Research Center (H Okati-Aliabad PhD), Zahedan University of Medical Sciences, Zahedan, Iran; Department of Epidemiology and Population Health (A Oulhaj PhD), Khalifa University, Abu Dhabi, United Arab Emirates; Universidad San Ignacio de Loyola, Lima, Peru (K Pacheco-Barrios MD); Yonsei University College of Medicine (S Park BEng), Yonsei University, Seodaemun-gu, South Korea; Global Health Governance Programme (J Patel MChD), University of Edinburgh, Edinburgh, UK; School of Dentistry (J Patel MChD), University of Leeds, Leeds, UK; Department of Genetics (S Pawar PhD), Yale University, New Haven, CT, USA; Clinical Research Department (P Pedersini MSc, J H Villafane PhD), IRCCS Fondazione Don Carlo Gnocchi, Milan, Italy; International Institute for Educational Planning (Prof M F P Peres MD), Albert Einstein Hospital, Sao Paulo, Brazil; Department of Statistics and Econometrics (I Petcu PhD), Bucharest University of Economic Studies, Bucharest, Romania; Faculty of Medicine (F E Petermann-Rocha PhD), Diego Portales University, Santiago, Chile; BHF Glasgow Cardiovascular Research Centre (F E Petermann-Rocha PhD), University of Glasgow, Glasgow, UK; Department of Occupational Health and Safety Engineering (M Poursadeqiyan PhD), Ardabil University of Medical Science, Ardabil, Iran; Department of Neonatology (I Qattea MD), Case Western Reserve University, Cleveland, OH, USA; Department of Molecular Biology and Biochemistry (M Qureshi MS), University of California Irvine, Irvine, CA, USA; Department of Population Science and Human Resource Development (M Rahman DrPH), University of Rajshahi, Rajshahi, Bangladesh; Department of Radiology (S Ramasamy MD), Loyola University Medical

Center, Maywood, IL, USA; University of Social Welfare and Rehabilitation Sciences, Tehran, Iran (V Rashedi PhD); Department of Biological Sciences (Prof E M M Redwan PhD), King Abdulaziz University, Jeddah, Egypt; Department of Protein Research (Prof E M M Redwan PhD), Research and Academic Institution, Alexandria, Egypt; School of Physiotherapy (D C Ribeiro PhD), University of Otago, Dunedin, New Zealand; Department of Clinical Research (L Roever PhD), Federal University of Uberlandia, Uberlandia, Brazil; Department of Psychosocial Science (D Sagoe PhD), University of Bergen, Bergen, Norway; Health Policy Research Center (S Shahabi PhD), Shiraz University of Medical Sciences, Shiraz, Iran; Faculty of Medicine and Health (S Sharma PhD), University of New South Wales, Sydney, NSW, Australia; Department of Physiotherapy (S Sharma PhD), Kathmandu University, Dhulikhel, Nepal; Finnish Institute of Occupational Health, Helsinki, Finland (R Shiri PhD); Research and Development Department (D R Sunuwar MSc), Armed Police Force Hospital, Kathmandu, Nepal; Department of Public Health (D R Sunuwar MSc), Asian College for Advance Studies, Purbanchal University, Lalitpur, Nepal; Department of Pharmacology (M Tabish MPharm), Shaqra University, Shaqra, Saudi Arabia; Rheumatology and Immunology Unit (S Tharwat MD), Mansoura University, Mansoura, Egypt; Department of Life Sciences (I Ullah PhD), University of Management and Technology, Lahore, Pakistan; Clinical Cancer Research Center (S Valadan Tahbaz PhD), Milad General Hospital, Tehran, Iran; UKK Institute, Tampere, Finland (Prof T J Vasankari MD); Faculty of Medicine and Health Technology (Prof T J Vasankari MD), Tampere University, Tampere, Finland; Department of Physical Therapy (T Wiangkham PhD), Naresuan University, Phitsanulok, Thailand; Department of Neuropsychopharmacology (N Yonemoto PhD), National Center of Neurology and Psychiatry, Kodaira, Japan; Department of Public Health (N Yonemoto PhD), Juntendo University, Tokyo, Japan; Macquarie Medical School (Y You PhD), Macquarie University, Sydney, NSW, Australia; Research and Development Department (I Zare BSc), Sina Medical Biochemistry Technologies, Shiraz, Iran; Centre for Health Policy (Prof P M Brooks MD), University of Melbourne, Melbourne, VIC, Australia.

Author's contributions

Providing data or critical feedback on data sources

Aidin Abedi, Janardhana P Aithala, Mostafa Akbarzadeh-Khiavi, Jalal Arabloo, Krishna K Aryal, Seyyed Shamsadin Athari, Alok Atreya, Mainak Bardhan, Alehegn Bekele Bekele, Rajbir Bhatti, Souad Bouaoud, Peter M Brooks, Marita Cross, Garland T Culbreth, Xiaochen Dai, Meghnath Dhimal, Thanh Chi Do, Karsten E Dreinhoefer, Adeniyi Francis Fagbamigbe, Balasankar Ganesan, Ahmad Ghashghaee, Tiffany K Gill, Simon Matthew Graham, Netanja I Harlianto, Ahmed I Hasaballah, Himanshu Khajuria, Ali Kiadaliri, Adnan Kisa, Kewal Krishan, Vijay Krishnamoorthy, Thao Thi Thu Le, Yo Han Lee, Stephen S Lim, Jeadran N Malagón-Rojas, Iram Malik, Mohamed Kamal Mesregah, Awoke Misganaw, Manasi Murthy Mittinty, Ali H Mokdad, Sara Momtazmanesh, Mohammad Ali Moni, Ebrahim Mostafavi, Jibran Sualeh Muhammad, Christopher J L Murray, Shumaila Nargus, Robina Khan Niazi, Kevin Pacheco-Barrios, Seoyeon Park, Jay Patel, Shrikant Pawar, Mario F P Peres, Maryam Faiz Qureshi, Shahram Rahimi- Dehgolan, Shakthi Kumaran Ramasamy, Vahid Rashedi, Elrashdy Moustafa Mohamed Redwan, Leonardo Roever, Fatemeh Saheb Sharif-Askari, Amir Shafaat, Ambrish Singh, Mohammad Tabish, Irfan Ullah, Sahel Valadan Tahbaz, Tommi Juhani Vasankari, Theo Vos, Taweewat Wiangkham, Naohiro Yonemoto, and Iman Zare.

Developing methods or computational machinery

Aleksandr Y Aravkin, Souad Bouaoud, Peter M Brooks, Garland T Culbreth, Xiaochen Dai, Thanh Chi Do, Karsten E Dreinhoefer, Simon I Hay, Md Jobair Khan, Sorour Khateri, Adnan Kisa, Thao Thi Thu Le, Ali H Mokdad, Mohammad Ali Moni, Yousef Moradi, Christopher J L Murray, Seoyeon Park, Shakthi Kumaran Ramasamy, Amir Shafaat, Jaimie D Steinmetz, Theo Vos, and Peng Zheng.

Providing critical feedback on methods or results

Mohammadreza Abbasian, Miracle Ayomikun Adesina, Janardhana P Aithala, Mostafa Akbarzadeh- Khiavi, Yazan Al Thaher, Tariq A Alalwan, Hosam Alzahrani, Sohrab Amiri, Benny Antony, Jalal Arabloo, Ashokan Arumugam, Krishna K Aryal, Seyyed Shamsadin Athari, Alok Atreya, Soroush Baghdadi, Mainak Bardhan, Lope H Barrero, Lindsay M Bearne, Alehegn Bekele Bekele, Isabela M Bensenor, Pankaj Bhardwaj, Rajbir

Bhatti, Ali Bijani, Souad Bouaoud, Andrew M Briggs, Peter M Brooks, Huzaifa Ahmad Cheema, Isaac Sunday Chukwu, Benjamin Clarsen, Marita Cross, Garland T Culbreth, Xiaochen Dai, Katie de Luca, Meghnath Dhimal, Thanh Chi Do, Karsten E Dreinhoefer, Adeniyi Francis Fagbamigbe, Siamak Farokh Forghani, Nuno Ferreira, Balasankar Ganesan, Mesfin Gebrehiwot, Ahmad Ghashghaee, Tiffany K Gill, Simon Matthew Graham, Netanja I Harlianto, Jan Hartvigsen, Ahmed I Hasaballah, Mohammed Bheser Hassen, Simon I Hay, Mohammad Heidari, Alexander Kevin Hsiao, Irena M Ilic, Mohammad Jokar, Himanshu Khajuria, Praval Khanal, Sorour Khateri, Ali Kiadaliri, Min Seo Kim, Adnan Kisa, Ali-Asghar Kolahi, Kewal Krishan, Vijay Krishnamoorthy, Bagher Larijani, Thao Thi Thu Le, Yo Han Lee, Stephen S Lim, Iram Malik, Hamid Reza Marateb, Ashish J Mathew, Mohamed Kamal Mesregah, Tomislav Mestrovic, Alireza Mirahmadi, Awoke Misganaw, Manasi Murthy Mittinty, Sadra Mohaghegh, Ali H Mokdad, Kaveh Momenzadeh, Sara Momtazmanesh, Mohammad Ali Moni, Yousef Moradi, Ebrahim Mostafavi, Jibran Sualeh Muhammad, Christopher J L Murray, Sathish Muthu, Shumaila Nargus, Hasan Nassereldine, Subas Neupane, Robina Khan Niazi, In-Hwan Oh, Hassan Okati-Aliabad, Abderrahim Oulhaj, Kevin Pacheco-Barrios, Seoyeon Park, Jay Patel, Shrikant Pawar, Paolo Pedersini, Mario F P Peres, Ionela-Roxana Petcu, Fanny Emily Petermann-Rocha, Ibrahim Qattea, Maryam Faiz Qureshi, Shahram Rahimi-Dehgolan, Mosiur Rahman, Shakthi Kumaran Ramasamy, Elrashdy Moustafa Mohamed Redwan, Daniel Cury Ribeiro, Leonardo Roever, Azam Safary, Fatemeh Saheb Sharif-Askari, Sana Salehi, Amir Shafaat, Saeed Shahabi, Saurab Sharma, Bereket Beyene Shashamo, Rahman Shiri, Ambrish Singh, Jaimie D Steinmetz, Dev Ram Sunuwar, Mohammad Tabish, Samar Tharwat, Irfan Ullah, Sahel Valadan Tahbaz, Jorge Hugo Villafañe, Theo Vos, Taweewat Wiangkham, Anthony D Woolf, Naohiro Yonemoto, and Yuyi You.

Drafting the work or revising it critically for important intellectual content

Mohammadreza Abbasian, Aidin Abedi, Janardhana P Aithala, Yazan Al Thaher, Hosam Alzahrani, Sohrab Amiri, Benny Antony, Jalal Arabloo, Ashokan Arumugam, Seyyed Shamsadin Athari, Alok Atreya, Soroush Baghdadi, Mainak Bardhan, Lope H Barrero, Lindsay M Bearne, Alehegn Bekele Bekele, Neil Betteridge, Rajbir Bhatti, Andrew M Briggs, Peter M Brooks, Huzaifa Ahmad Cheema, Steffan Wittrup McPhee Christensen, Benjamin Clarsen, Marita Cross, Garland T Culbreth, Katie de Luca, Belay Desye, Meghnath Dhimal, Thanh Chi Do, Adeniyi Francis Fagbamigbe, Siamak Farokh Forghani, Nuno Ferreira, Balasankar Ganesan, Mesfin Gebrehiwot, Tiffany K Gill, Lydia M Haile, Netanja I Harlianto, Jan Hartvigsen, Ahmed I Hasaballah, Mohammad Hasanian, Simon I Hay, Mohammad Heidari, Alexander Kevin Hsiao, Irena M Ilic, Praval Khanal, Ali Kiadaliri, Min Seo Kim, Adnan Kisa, Jacek A Kopec, Deborah R Kopansky-Giles, Kewal Krishan, Iván Landires, Thao Thi Thu Le, Justin Lo, Seyedeh Panid Madani, Jeadran N Malagón-Rojas, Hamid Reza Marateb, Lyn M March, Ashish J Mathew, Tuomo J Meretoja, Mohamed Kamal Mesregah, Tomislav Mestrovic, Alireza Mirahmadi, Awoke Misganaw, Manasi Murthy Mittinty, Sadra Mohaghegh, Ali H Mokdad, Kaveh Momenzadeh, Sara Momtazmanesh, Lorenzo Monasta, Mohammad Ali Moni, Ebrahim Mostafavi, Jibran Sualeh Muhammad, Christopher J L Murray, Sathish Muthu, Shumaila Nargus, Hasan Nassereldine, Subas Neupane, Robina Khan Niazi, Kevin Pacheco-Barrios, Seoyeon Park, Jay Patel, Shrikant Pawar, Paolo Pedersini, Ionela-Roxana Petcu, Fanny Emily Petermann-Rocha, Mohsen Poursadeqiyan, Maryam Faiz Qureshi, Quinn Rafferty, Shakthi Kumaran Ramasamy, Vahid Rashedi, Elrashdy Moustafa Mohamed Redwan, Daniel Cury Ribeiro, Leonardo Roever, Dominic Sagoe, Fatemeh Saheb Sharif-Askari, Amirhossein Sahebkar, Amir Shafaat, Saeed Shahabi, Saurab Sharma, Bereket Beyene Shashamo, Helen Slater, Amanda E Smith, Jaimie D Steinmetz, Dev Ram Sunuwar, Mohammad Tabish, Samar Tharwat, Irfan Ullah, Sahel Valadan Tahbaz, Tommi Juhani Vasankari, Jorge Hugo Villafañe, Stein Emil Vollset, Theo Vos, Taweewat Wiangkham, Anthony D Woolf, Naohiro Yonemoto, Yuyi You, and Iman Zare.

Managing the estimation or publications process

Marita Cross, Garland T Culbreth, Siamak Farokh Forghani, Tiffany K Gill, Hailey Hagins, Simon I Hay, Thao Thi Thu Le, Manasi Murthy Mittinty, Ali H Mokdad, Christopher J L Murray, Shakthi Kumaran Ramasamy, Amir Shafaat, Jaimie D Steinmetz, Theo Vos, and Anthony D Woolf.

Section 5. Methods

Supplemental Table 1: ICD codes

Condition	ICD-10 codes	ICD-9 codes
**Lupus erythematosus**	L93	710.0
**Infectious arthropathies**	M00-M02	711
**Inflammatory polyarthropathies**	M03, M06-M09, M11-M15	712-713, 446
**Other joint disorders**	M20-M25	716-719
**Systemic connective tissue disorders**	M30-M36	710.1-710.9
**Deforming dorsopathies**	M40-M43, I27.1	737, 416
**Spondylopathies**	M45-M46	720
**Disorders of muscles**	M61-M63	725
**Disorders of synovium and tendon**	M65-M68	726-728
**Other soft tissue disorders**	M70-M73, M75-M79	729
**Disorders of bone density and structure**	M80-85	733.0-2
**Osteomyelitis**	M86	730.1-730.3, 730.7-9
**Other osteopathies**	M87-M90	733.3-9
**Chondropathies**	M91-M94	732
**Other disorders of the MSK system and connective tissue**	M95, M99	734-736, 738-9

Supplemental Table 2: Data sources

Australian Bureau of Statistics. Australia National Health Survey 1995. Canberra, Australia: Australian Bureau of Statistics.
Agency for Healthcare Research and Quality. United States Medical Expenditure Panel Survey 1996. Rockville, United States: Agency for Healthcare Research and Quality.
Agency for Healthcare Research and Quality. United States Medical Expenditure Panel Survey 1997. Rockville, United States: Agency for Healthcare Research and Quality.
Agency for Healthcare Research and Quality. United States Medical Expenditure Panel Survey 1998. Rockville, United States: Agency for Healthcare Research and Quality.
Agency for Healthcare Research and Quality. United States Medical Expenditure Panel Survey 1999. Rockville, United States: Agency for Healthcare Research and Quality.
Agency for Healthcare Research and Quality. United States Medical Expenditure Panel Survey 2000. Rockville, United States: Agency for Healthcare Research and Quality.
Agency for Healthcare Research and Quality. United States Medical Expenditure Panel Survey 2001. Rockville, United States: Agency for Healthcare Research and Quality.
Agency for Healthcare Research and Quality. United States Medical Expenditure Panel Survey 2002. Rockville, United States: Agency for Healthcare Research and Quality.
Agency for Healthcare Research and Quality. United States Medical Expenditure Panel Survey 2003. Rockville, United States: Agency for Healthcare Research and Quality.
Agency for Healthcare Research and Quality. United States Medical Expenditure Panel Survey 2004. Rockville, United States: Agency for Healthcare Research and Quality.
Agency for Healthcare Research and Quality. United States Medical Expenditure Panel Survey 2005. Rockville, United States: Agency for Healthcare Research and Quality.
Agency for Healthcare Research and Quality. United States Medical Expenditure Panel Survey 2006. Rockville, United States: Agency for Healthcare Research and Quality.
Agency for Healthcare Research and Quality. United States Medical Expenditure Panel Survey 2007. Rockville, United States: Agency for Healthcare Research and Quality.
Agency for Healthcare Research and Quality. United States Medical Expenditure Panel Survey 2008. Rockville, United States: Agency for Healthcare Research and Quality.
Agency for Healthcare Research and Quality. United States Medical Expenditure Panel Survey 2009. Rockville, United States: Agency for Healthcare Research and Quality.
Agency for Healthcare Research and Quality. United States Medical Expenditure Panel Survey 2010. Rockville, United States: Agency for Healthcare Research and Quality.

Australian Bureau of Statistics. Australia National Health Survey 2001. Canberra, Australia: Australian Bureau of Statistics.
Australian Bureau of Statistics. Australia National Health Survey 2004-2005.
Australian Bureau of Statistics. Australia National Health Survey 2007-2008. Canberra, Australia: Australian Bureau of Statistics.
Chaiamnuay P, Darmawan J, Muirden KD, Assawatanabodee P. Epidemiology of rheumatic disease in rural Thailand: a WHO-ILAR COPCORD study. Community Oriented Programme for the Control of Rheumatic Disease. J Rheumatol. 1998; 25(7): 1382-7.
Davatchi F, Jamshidi A-R, Banihashemi AT, Gholami J, Forouzanfar MH, Akhlaghi M, Barghamdi M, Noorolahzadeh E, Khabazi A-R, Salesi M, Salari A-H, Karimifar M, Essalat-Manesh K, Hajialiloo M, Soroosh M, Farzad F, Moussavi H-R, Samadi F, Ghaznavi K, Asgharifard H, Zangiabadi A-H, Shahram F, Nadji A, Akbarian M, Gharibdoost F. WHO-ILAR COPCORD Study (Stage 1, Urban Study) in Iran. J Rheumatol. 2008; 35(7): 1384.
Al-Awadhi AM, Olusi SO, Moussa M, Shehab D, Al-Zaid N, Al-Herz A, Al-Jarallah K. Musculoskeletal pain, disability and health-seeking behavior in adult Kuwaitis using a validated Arabic version of the WHO-ILAR COPCORD Core Questionnaire. Clin Exp Rheumatol. 2004; 22(2): 177-83.
Haq SA, Darmawan J, Islam MN, Uddin MZ, Das BB, Rahman F, Chowdhury MAJ, Alam MN, Mahmud TAK, Chowdhury MR, Tahir M. Prevalence of rheumatic diseases and associated outcomes in rural and urban communities in Bangladesh: a COPCORD study. J Rheumatol. 2005; 32(2): 348-53.
Farooqi A, Gibson T. Prevalence of the major rheumatic disorders in the adult population of north Pakistan. Br J Rheumatol. 1998; 37(5): 491-5.
Wigley R, Manahan L, Muirden KD, Caragay R, Pinfold B, Couchman KG, Valkenburg HA. Rheumatic disease in a Philippine village. II: a WHO-ILAR-APLAR COPCORD study, phases II and III. Rheumatol Int. 1991; 11(4-5): 157-61.
Minh Hoa TT, Darmawan J, Chen SL, Van Hung N, Thi Nhi C, Ngoc An T, Damarwan J, Shun Le C. Prevalence of the rheumatic diseases in urban Vietnam: a WHO-ILAR COPCORD study. J Rheumatol. 2003; 30(10): 2252-6.
Salaffi F, De Angelis R, Grassi W, MArche Pain Prevalence, INvestigation Group (MAPPING) study. Prevalence of musculoskeletal conditions in an Italian population sample: results of a regional community-based study. I. The MAPPING study. Clin Exp Rheumatol. 2005; 23(6): 819-28.
Bennett K, Cardiel MH, Ferraz MB, Riedemann P, Goldsmith CH, Tugwell P. Community screening for rheumatic disorder: cross cultural adaptation and screening characteristics of the COPCORD Core Questionnaire in Brazil, Chile, and Mexico. J Rheumatol. 1997; 24(1): 160-8.
Dans LF, Tankeh-Torres S, Amante CM, Penserga EG. The prevalence of rheumatic diseases in a Filipino urban population: a WHO-ILAR COPCORD Study. World Health Organization. International League of Associations for Rheumatology. Community Oriented Programme for the Control of the Rheumatic Diseases. J Rheumatol. 1997; 24(9): 1814-9.
Joshi VL, Chopra A. Is there an urban-rural divide? Population surveys of rheumatic musculoskeletal disorders in the Pune region of India using the COPCORD Bhigwan model. J Rheumatol. 2009; 36(3): 614-22.

Reyes Llerena GA, Guibert Toledano M, Hernández Martínez AA, González Otero ZA, Alcocer Varela J, Cardiel MH, Community Oriented Programme for the Control of Rheumatic Diseases. Prevalence of musculoskeletal complaints and disability in Cuba. A community-based study using the COPCORD core questionnaire. Clin Exp Rheumatol. 2000; 18(6): 739-42.
Ministry of Health (New Zealand), National Research Bureau Ltd (New Zealand). New Zealand Health Survey 2006-2007.
Agency for Healthcare Research and Quality. United States Medical Expenditure Panel Survey 2011. Rockville, United States of America: Agency for Healthcare Research and Quality.
Statistics Norway. Norway Survey of Living Conditions 2008-2009. Oslo, Norway: Statistics Norway.
Statistics Norway. Norway Survey of Living Conditions 2005-2006. Oslo, Norway: Statistics Norway.
Statistics Norway. Norway Survey of Living Conditions 2002. Oslo, Norway: Statistics Norway.
Alvarez-Nemegyei J, Pelaez-Ballestas I, Sanin LH, Cardiel MH, Ramirez-Angulo A, Goycochea-Robles M-V. Prevalence of musculoskeletal pain and rheumatic diseases in the southeastern region of Mexico. A COPCORD-based community survey. J Rheumatol. 2011; 86(Suppl): 21-5.
Cakır N, Pamuk ÖN, Derviş E, Imeryüz N, Uslu H, Benian Ö, Elelçi E, Erdem G, Sarvan FO, Senocak M. The prevalences of some rheumatic diseases in western Turkey: Havsa study. Rheumatol Int. 2012; 32(4): 895-908.
Statistics Norway. Norway Survey of Living Conditions 2012-2013.
Granados Y, Cedeno L, Rosillo C, Berbin S, Azocar M, Molina ME, Lara O, Sanchez G, Pelaez-Ballestas I. Prevalence of musculoskeletal disorders and rheumatic diseases in an urban community in Monagas State, Venezuela: a COPCORD study. Clin Rheumatol. 2015; 34(5): 871-7.
Ministry of Health (Sri Lanka). Sri Lanka Indoor Morbidity and Mortality Return 2014. Colombo, Sri Lanka: Ministry of Health (Sri Lanka).
Ministry of Health (Sri Lanka). Sri Lanka Indoor Morbidity and Mortality Return 2015. Colombo, Sri Lanka: Ministry of Health (Sri Lanka).
Kumar P, Alok R, Das SK, Srivastava R, Agarwal GG. Distribution of rheumatological diseases in rural and urban areas: An adapted COPCORD Stage I Phase III survey of Lucknow district in north India. Int J Rheum Dis. 2018; 21(11): 1894-1899.
Institute for Health Metrics and Evaluation (IHME). IHME Modeled Excess Mortality Rate (EMR).
Ministry of Health and Welfare (Taiwan). Taiwan National Health Insurance Claims Data 2016.
National Health Fund (Poland). Poland National Health Fund Patient Claims 2015.
National Health Fund (Poland). Poland National Health Fund Patient Claims 2016.
National Health Fund (Poland). Poland National Health Fund Patient Claims 2017.

Ministry of Health (Russian Federation). Russia Statistical Yearbook 2010. 2011.
National Health Fund (Poland). Poland National Health Fund Patient Claims 2018.

Supplemental Table 3: Summary of covariates used in the other MSK DisMod-MR meta-regression model

Covariate	Type	Parameter	Exponentiated beta (95% uncertainty interval)
Healthcare Access and Quality Index	Country-level	Excess mortality rate	0.95 (0.94–0.96)
Socio-demographic index	Country-level	Prevalence	2.71 (2.69–2.72)

Supplemental Table 4: Severity distribution and associated disability weights (DW)

Severity level	Lay description	DW (95% CI)	Proportions
Asymptomatic			0.28 (0.27–0.29)
Musculoskeletal problems, upper limbs			
Mild	This person has mild pain and stiffness in the arms and hands. The person has some difficulty lifting, carrying, and holding things.	0.028 (0.017–0.046)	0.20 (0.15–0.29)
Moderate	This person has moderate pain and stiffness in the arms and hands, which causes difficulty lifting, carrying, and holding things, and trouble sleeping because of the pain.	0.115 (0.079–0.163)	0.10 (0.06–0.15)
Musculoskeletal problems, lower limbs			
Mild	This person has pain in the leg, which causes some difficulty running, walking long distances, and getting up and down.	0.023 (0.013–0.040)	0.22 (0.15–0.30)
Severe	This person has severe pain in the leg, which makes the person limp and causes a lot of difficulty walking, standing, lifting and carrying heavy things, getting up and down, and sleeping.	0.163 (0.109–0.224)	0.06 (0.04–0.07)

Musculoskeletal problems, generalised			
Moderate	This person has pain and deformity in most joints, causing difficulty moving around, getting up and down, and using the hands for lifting and carrying. The person often feels fatigue.	0.312 (0.201–0.438)	0.07 (0.06–0.08)
Severe	This person has severe, constant pain and deformity in most joints, causing difficulty moving around, getting up and down, eating, dressing, lifting, carrying, and using the hands. The person often feels sadness, anxiety, and extreme fatigue.	0.572 (0.370–0.758)	0.07 (0.07–0.08)

Supplemental Table 5: Validation experiment for forecasting method

Site of osteoarthritis	RMSE	Bias
Hand	0.0075	0.00007
Hip	0.0005	0.00003
Knee	0.0022	0.00004
Other	0.0004	0.00005

Section 6. Results

Supplemental Table 6: Counts and age-standardised rates of prevalence and death in 2020 for other MSK disorders, by region and by country

Location	Number of prevalent cases (95% UI)	Age-standardised prevalence rate per 100 000 (95% UI)	Number of deaths (95% UI)	Age-standardised rate of deaths per 100 000 (95% UI)
Global	494 000 000 (431 000 000–564 000 000)	5910 (5180–6750)	83 100 (73 600–91 600)	1.0 (0.9–1.1)
Central Europe, eastern Europe, and central Asia	10 700 000 (8 660 000–13 100 000)	2020 (1660–2430)	4820 (4420–5170)	0.8 (0.7–0.9)
Central Asia	2 180 000 (1 820 000–2 630 000)	2390 (2010–2870)	320 (284–406)	0.6 (0.5–0.6)
Armenia	95 700 (79 300–116 000)	2540 (2110–3080)	13.3 (11.9–14.8)	0.3 (0.3–0.4)
Azerbaijan	299 000 (250 000–355 000)	2630 (2210–3130)	1.82 (0.395–2.58)	0.0 (0.0–0.0)
Georgia	92 700 (73 700–117 000)	1970 (1570–2430)	28 (24.7–31.2)	0.4 (0.4–0.4)
Kazakhstan	430 000 (346 000–532 000)	2250 (1810–2750)	88.3 (75.9–179)	0.6 (0.5–1.0)
Kyrgyzstan	144 000 (120 000–170 000)	2460 (2080–2920)	63.7 (53.4–72.6)	1.5 (1.3–1.7)
Mongolia	60 300 (49 400–74 400)	1930 (1560–2380)	23.3 (16.5–33)	1.4 (1.0–2.0)
Tajikistan	172 000 (140 000–206 000)	2230 (1840–2670)	5.71 (2.51–8.53)	0.2 (0.1–0.2)
Turkmenistan	125 000 (106 000–149 000)	2620 (2210–3150)	7.68 (6.21–9.25)	0.2 (0.2–0.3)
Uzbekistan	763 000 (638 000–914 000)	2530 (2130–3020)	87.8 (72.5–106)	0.8 (0.6–0.9)
Central Europe	3 060 000 (2 540 000–3 710 000)	2060 (1750–2420)	929 (833–1 030)	0.5 (0.4–0.5)
Albania	64 300 (52 700–79 600)	1810 (1480–2220)	25.2 (16.5–41.9)	0.7 (0.4–1.1)
Bosnia and Herzegovina	89 800 (71 800–117 000)	1960 (1570–2470)	26.6 (18–35.4)	0.5 (0.3–0.7)
Bulgaria	183 000 (143 000–226 000)	1870 (1500–2250)	24.9 (21.8–28.9)	0.2 (0.2–0.2)
Croatia	109 000 (83 500–142 000)	1990 (1580–2450)	55 (47.3–62.3)	0.6 (0.6–0.7)
Czechia	268 000 (209 000–346 000)	1960 (1550–2390)	79.1 (66.9–91.9)	0.4 (0.3–0.4)
Hungary	259 000 (196 000–328 000)	2050 (1620–2540)	164 (137–195)	0.9 (0.7–1.1)
Montenegro	16 200 (13 100–20 700)	2040 (1640–2540)	1.85 (1.33–2.36)	0.2 (0.2–0.3)
North Macedonia	58 200 (45 600–72 300)	2070 (1650–2490)	10.9 (8.07–14)	0.4 (0.3–0.5)

Poland	1 100 000<br>(981 000–1 260 000)	2270<br>(2030–2530)	277<br>(249–337)	0.4<br>(0.4–0.6)
Romania	490 000<br>(397 000–609 000)	1910<br>(1590–2360)	101<br>(82.9–116)	0.3<br>(0.2–0.3)
Serbia	246 000<br>(192 000–300 000)	2130<br>(1710–2580)	103<br>(83–131)	0.8<br>(0.6–1.0)
Slovakia	128 000<br>(99 900–160 000)	1840<br>(1490–2250)	26.9<br>(20–34.3)	0.3<br>(0.2–0.4)
Slovenia	45 400<br>(34 600–59 000)	1740<br>(1350–2140)	33<br>(27.1–39.3)	0.7<br>(0.6–0.9)
Eastern Europe	5 440 000<br>(4 280 000–6 770 000)	1880<br>(1510–2320)	3570<br>(3230–3850)	1.1<br>(1.0–1.2)
Belarus	241 000<br>(188 000–299 000)	1920<br>(1490–2370)	5.18<br>(4.53–5.92)	0.0<br>(0.0–0.0)
Estonia	41 200<br>(32 100–52 200)	2310<br>(1880–2890)	34.3<br>(29.3–39.9)	1.3<br>(1.1–1.6)
Latvia	56 600<br>(45 200–71 800)	2160<br>(1730–2720)	36.3<br>(31–40.9)	1.0<br>(0.8–1.1)
Lithuania	78 800<br>(60 500–98 100)	2110<br>(1700–2620)	63.4<br>(53–71.8)	1.2<br>(1.0–1.4)
Republic of Moldova	112 000<br>(89 000–138 000)	2250<br>(1830–2760)	38.1<br>(33–44.2)	0.7<br>(0.6–0.8)
Russia	3 740 000<br>(2 900 000–4 640 000)	1870<br>(1490–2300)	3130<br>(2830–3430)	1.4<br>(1.3–1.5)
Ukraine	1 180 000<br>(942 000–1 450 000)	1850<br>(1510–2290)	263<br>(203–327)	0.4<br>(0.3–0.5)
High income	106 000 000<br>(94 100 000–119 000 000)	7730<br>(6980–8560)	25 100<br>(21 800–26 900)	1.1<br>(1.0–1.2)
Australasia	2 880 000<br>(2 510 000–3 310 000)	7500<br>(6680–8520)	871<br>(745–947)	1.6<br>(1.4–1.7)
Australia	2 660 000<br>(2 320 000–3 030 000)	8290<br>(7380–9420)	739<br>(626–810)	1.6<br>(1.4–1.7)
New Zealand	225 000<br>(179 000–283 000)	3560<br>(2840–4470)	132<br>(116–148)	1.6<br>(1.4–1.8)
High income Asia Pacific	22 200 000<br>(19 200 000–25 400 000)	8180<br>(7130–9350)	4210<br>(3460–4750)	0.9<br>(0.7–1.0)
Brunei	35 400<br>(30 200–40 800)	7520<br>(6480–8560)	4.24<br>(3.26–5.42)	1.4<br>(1.1–1.9)
Japan	15 900 000<br>(13 800 000–18 100 000)	8340<br>(7300–9560)	3180<br>(2610–3480)	0.8<br>(0.7–0.9)
South Korea	5 630 000<br>(4 710 000–6 560 000)	7650<br>(6500–8840)	980<br>(715–1330)	1.2<br>(0.8–1.6)
Singapore	632 000<br>(554 000–727 000)	8240<br>(7250–9390)	47.9<br>(42.7–52.6)	0.6<br>(0.6–0.7)
High income North America	49 200 000<br>(46 000 000–53 000 000)	11 100<br>(10 500–11 800)	7160<br>(6350–7650)	1.2<br>(1.1–1.3)
Canada	5 140 000<br>(4 460 000–5 800 000)	11 100<br>(9820–12 500)	919<br>(801–1000)	1.3<br>(1.2–1.4)
Greenland	6680<br>(5670–7590)	10 200<br>(8840–11 500)	1.04<br>(0.689–1.37)	1.7<br>(1.1–2.2)
USA	44 400 000<br>(41 800 000–47 500 000)	11 100<br>(10 500–11 700)	6240<br>(5560–6650)	1.2<br>(1.1–1.2)

Southern Latin America	8 020 000<br>(7 030 000–9 030 000)	10 500<br>(9190–11 800)	784<br>(718–843)	1.0<br>(0.9–1.0)
Argentina	5 320 000<br>(4 610 000–6 000 000)	10 600<br>(9140–11 900)	492<br>(454–532)	0.9<br>(0.9–1.0)
Chile	2 290 000<br>(1 990 000–2 620 000)	10 400<br>(9060–11 900)	239<br>(214–264)	1.0<br>(0.9–1.1)
Uruguay	413 000<br>(360 000–466 000)	10 000<br>(8750–11 400)	52.8<br>(47.1–57.2)	1.0<br>(0.9–1.1)
Western Europe	23 800 000<br>(19 700 000–28 600 000)	4310<br>(3620–5060)	12 100<br>(10 400–13 100)	1.1<br>(1.0–1.2)
Andorra	4050<br>(3160–5090)	3650<br>(2940–4560)	0.174<br>(0.119–0.215)	0.1<br>(0.1–0.1)
Austria	451 000<br>(370 000–545 000)	4010<br>(3350–4790)	171<br>(146–187)	0.8<br>(0.7–0.9)
Belgium	484 000<br>(382 000–602 000)	3650<br>(2990–4440)	260<br>(214–290)	1.0<br>(0.8–1.1)
Cyprus	68 900<br>(56 600–83 400)	4 110<br>(3 380–4 920)	25.4<br>(19.1–33)	1.6<br>(1.3–2.0)
Denmark	345 000<br>(286 000–411 000)	4 640<br>(3 940–5 440)	175<br>(147–196)	1.4<br>(1.1–1.5)
Finland	237 000<br>(185 000–302 000)	3660<br>(3010–4460)	98.1<br>(79.9–109)	0.7<br>(0.6–0.8)
France	2 930 000<br>(2 310 000–3 630 000)	3820<br>(3100–4610)	3020<br>(2570–3380)	1.7<br>(1.5–1.9)
Germany	4 850 000<br>(3 990 000–5 890 000)	4440<br>(3730–5210)	1980<br>(1660–2210)	0.9<br>(0.8–1.0)
Greece	598 000<br>(492 000–713 000)	4260<br>(3620–5000)	147<br>(127–163)	0.5<br>(0.5–0.6)
Iceland	18 300<br>(15 400–21 700)	4380<br>(3750–5160)	5.85<br>(4.76–6.7)	0.9<br>(0.7–1.0)
Ireland	264 000<br>(216 000–318 000)	4460<br>(3730–5320)	107<br>(94.8–118)	1.4<br>(1.2–1.5)
Israel	443 000<br>(375 000–526 000)	4480<br>(3830–5250)	110<br>(95–120)	0.9<br>(0.8–0.9)
Italy	3 740 000<br>(3 040 000–4 420 000)	4570<br>(3830–5360)	1250<br>(1020–1360)	0.8<br>(0.7–0.9)
Luxembourg	32 600<br>(27 000–38 600)	4120<br>(3430–4820)	8.77<br>(6.69–9.88)	0.8<br>(0.6–0.8)
Malta	23 900<br>(19 800–28 700)	4100<br>(3450–4810)	8.01<br>(6.75–8.85)	0.8<br>(0.7–0.9)
Monaco	2500<br>(2050–2930)	4720<br>(4000–5530)	0.497<br>(0.369–0.643)	0.5<br>(0.3–0.6)
Netherlands	995 000<br>(795 000–1 220 000)	4610<br>(3850–5430)	494<br>(426–546)	1.3<br>(1.2–1.5)
Norway	227 000<br>(184 000–276 000)	3440<br>(2840–4160)	146<br>(124–160)	1.3<br>(1.1–1.4)
Portugal	617 000<br>(502 000–734 000)	4330<br>(3660–5090)	177<br>(151–196)	0.7<br>(0.6–0.7)
San Marino	1880<br>(1520–2270)	4480<br>(3710–5250)	0.371<br>(0.268–0.51)	0.5<br>(0.3–0.6)
Spain	2 520 000<br>(2 090 000–3 030 000)	4180<br>(3530–4980)	1300<br>(1090–1450)	1.1<br>(1.0–1.2)

Sweden	579 000 (478 000–684 000)	4540 (3850–5380)	227 (189–249)	1.0 (0.8–1.0)
Switzerland	460 000 (364 000–564 000)	4160 (3370–5050)	237 (186–267)	1.1 (0.9–1.2)
UK	3 900 000 (3 240 000–4 610 000)	4580 (3880–5400)	2110 (1840–2240)	1.5 (1.3–1.6)
Latin America and Caribbean	47 000 000 (41 500 000–53 200 000)	7480 (6610–8440)	8040 (7400–8690)	1.3 (1.2–1.4)
Andean Latin America	4 080 000 (3 550 000–4 650 000)	6480 (5670–7390)	456 (383–560)	0.8 (0.6–0.9)
Bolivia	682 000 (588 000–774 000)	6310 (5450–7130)	85.7 (58.4–113)	1.0 (0.7–1.3)
Ecuador	1 090 000 (954 000–1 240 000)	6360 (5560–7250)	168 (136–302)	1.1 (0.9–1.9)
Peru	2 300 000 (2 000 000–2 670 000)	6590 (5720–7640)	202 (149–266)	0.6 (0.4–0.8)
Caribbean	2 560 000 (2 260 000–2 940 000)	4980 (4390–5700)	802 (678–974)	1.6 (1.3–1.9)
Antigua and Barbuda	5820 (5100–6690)	5450 (4780–6240)	1.86 (1.66–2.06)	2.0 (1.8–2.2)
Bahamas	24 700 (21 400–28 600)	5700 (4970–6570)	12.4 (10.3–15.1)	3.3 (2.7–3.9)
Barbados	22 800 (19 900–26 300)	5680 (4930–6510)	12.6 (10.5–14.8)	2.9 (2.4–3.4)
Belize	18 300 (15 900–21 700)	4910 (4250–5760)	5.77 (4.95–7.68)	1.7 (1.4–2.1)
Bermuda	5570 (4810–6350)	5910 (5150–6800)	2.05 (1.74–2.38)	1.6 (1.3–1.9)
Cuba	812 000 (712 000–913 000)	5390 (4760–5990)	213 (182–244)	1.2 (1.1–1.4)
Dominica	4510 (3940–5200)	5580 (4880–6410)	0.921 (0.742–1.12)	1.1 (0.9–1.4)
Dominican Republic	536 000 (466 000–621 000)	5010 (4370–5790)	119 (84.3–158)	1.3 (0.9–1.7)
Grenada	6080 (5270–7000)	5240 (4540–5980)	2.21 (1.99–2.47)	2.4 (2.2–2.6)
Guyana	37 300 (32 100–43 300)	4940 (4260–5730)	6.75 (5.2–8.47)	1.0 (0.8–1.3)
Haiti	408 000 (344 000–487 000)	3940 (3340–4750)	196 (121–336)	2.1 (1.2–3.2)
Jamaica	165 000 (143 000–193 000)	5400 (4690–6270)	91.8 (74.6–115)	2.8 (2.2–3.5)
Puerto Rico	267 000 (229 000–310 000)	5610 (4860–6520)	58 (48.9–66.9)	0.9 (0.8–1.1)
Saint Kitts and Nevis	3940 (3410–4560)	5410 (4710–6270)	1.53 (1.32–1.77)	2.8 (2.5–3.2)
Saint Lucia	11 400 (9900–13 000)	5320 (4640–6070)	3.76 (3.18–4.46)	1.9 (1.6–2.2)
Saint Vincent and the Grenadines	6730 (5840–7830)	5130 (4470–5910)	2.56 (2.27–2.9)	2.2 (1.9–2.5)
Suriname	32 400 (28 000–38 100)	5190 (4490–6090)	11.4 (9.73–13.9)	2.0 (1.7–2.4)

Trinidad and Tobago	99 100<br>(87 100–115 000)	5680<br>(4990–6620)	30.4<br>(24.1–37.9)	1.8<br>(1.5–2.3)
Virgin Islands	8050<br>(6880–9270)	5790<br>(5020–6670)	2.49<br>(2.03–3.3)	1.9<br>(1.5–2.5)
Central Latin America	21 700 000<br>(19 200 000–24 600 000)	8390<br>(7440–9460)	3840<br>(3250–4270)	1.5<br>(1.3–1.7)
Colombia	4 420 000<br>(3 890 000–5 100 000)	8330<br>(7350–9610)	977<br>(843–1140)	1.8<br>(1.6–2.1)
Costa Rica	454 000<br>(393 000–519 000)	8530<br>(7390–9720)	84.3<br>(73.4–97.2)	1.6<br>(1.4–1.8)
El Salvador	461 000<br>(396 000–533 000)	7490<br>(6430–8700)	65.2<br>(54.5–75.4)	1.0<br>(0.9–1.2)
Guatemala	1 090 000<br>(929 000–1 270 000)	7280<br>(6300–8370)	105<br>(90.3–118)	0.8<br>(0.7–0.9)
Honduras	626 000<br>(542 000–722 000)	7690<br>(6730–8860)	129<br>(87.6–181)	1.8<br>(1.2–2.4)
Mexico	11 400 000<br>(10 100 000–12 800 000)	8730<br>(7750–9800)	1830<br>(1330–2090)	1.5<br>(1.1–1.7)
Nicaragua	442 000<br>(380 000–516 000)	7490<br>(6530–8670)	80<br>(64.3–92.6)	1.6<br>(1.3–1.8)
Panama	376 000<br>(329 000–427 000)	8720<br>(7650–9910)	48<br>(41.2–55.8)	1.1<br>(0.9–1.3)
Venezuela	2 430 000<br>(2 120 000–2 780 000)	7970<br>(6940–9030)	524<br>(408–673)	1.8<br>(1.4–2.3)
Tropical Latin America	18 700 000<br>(16 300 000–21 300 000)	7290<br>(6350–8280)	2940<br>(2780–3190)	1.2<br>(1.1–1.3)
Brazil	18 200 000<br>(15 800 000–20 800 000)	7280<br>(6350–8270)	2850<br>(2670–3100)	1.2<br>(1.1–1.3)
Paraguay	488 000<br>(423 000–563 000)	7320<br>(6380–8350)	97.7<br>(67.9–120)	1.5<br>(1.1–1.9)
North Africa and Middle East	27 900 000<br>(24 000 000–32 700 000)	4680<br>(3990–5470)	3320<br>(2680–4000)	0.7<br>(0.6–0.9)
North Africa and Middle East	27 900 000<br>(24 000 000–32 700 000)	4680<br>(3990–5470)	3320<br>(2680–4000)	0.7<br>(0.6–0.9)
Afghanistan	565 000<br>(223 000–863 000)	2350<br>(845–3500)	279<br>(169–425)	1.2<br>(0.8–1.8)
Algeria	2 180 000<br>(1 900 000–2 540 000)	5060<br>(4370–5860)	184<br>(134–222)	0.5<br>(0.4–0.7)
Bahrain	101 000<br>(86 400–120 000)	5420<br>(4750–6250)	12.3<br>(8.08–14.5)	1.7<br>(1.1–2.1)
Egypt	4 510 000<br>(3 910 000–5 140 000)	5120<br>(4450–5830)	221<br>(169–256)	0.3<br>(0.2–0.4)
Iran	4 270 000<br>(3 660 000–5 050 000)	4550<br>(3920–5300)	549<br>(363–625)	0.7<br>(0.5–0.9)
Iraq	1 330 000<br>(1 000 000–1 720 000)	3680<br>(2780–4770)	158<br>(118–210)	0.6<br>(0.4–0.7)
Jordan	575 000<br>(506 000–665 000)	5490<br>(4880–6260)	70.4<br>(57.7–86.5)	1.0<br>(0.8–1.2)
Kuwait	317 000<br>(267 000–379 000)	5910<br>(5110–6760)	15.7<br>(13.5–20.9)	0.5<br>(0.5–0.6)
Lebanon	273 000<br>(209 000–330 000)	4930<br>(3780–5960)	26.2<br>(20.6–31.9)	0.5<br>(0.4–0.6)

Libya	358 000 (306 000–431 000)	4780 (4090–5670)	43.4 (26.9–62.9)	0.7 (0.5–1.0)
Morocco	1 790 000 (1 520 000–2 090 000)	4740 (4040–5540)	274 (130–516)	0.9 (0.4–1.7)
Oman	230 000 (188 000–276 000)	4800 (4050–5550)	24.2 (13–49.4)	1.2 (0.8–1.6)
Palestine	149 000 (113 000–186 000)	3860 (2980–4840)	9.57 (5.7–11.2)	0.4 (0.2–0.4)
Qatar	170 000 (142 000–205 000)	4980 (4250–5750)	2.36 (1.43–3.95)	0.2 (0.1–0.3)
Saudi Arabia	1 700 000 (1 360 000–2 110 000)	3860 (3120–4700)	249 (180–345)	0.9 (0.7–1.2)
Sudan	1 360 000 (1 150 000–1 620 000)	4550 (3890–5380)	185 (122–284)	0.7 (0.5–1.0)
Syria	540 000 (404 000–687 000)	3770 (2830–4800)	17.9 (13.1–24)	0.2 (0.1–0.2)
Tunisia	698 000 (598 000–810 000)	5180 (4450–6000)	68.6 (39.8–123)	0.6 (0.3–1.0)
Türkiye	5 190 000 (4 510 000–5 980 000)	5380 (4690–6200)	808 (621–1060)	1.0 (0.7–1.2)
United Arab Emirates	623 000 (502 000–754 000)	4950 (4270–5780)	22.5 (17.4–29.2)	0.6 (0.4–0.7)
Yemen	917 000 (758 000–1 110 000)	4010 (3330–4830)	95.3 (52.4–153)	0.5 (0.3–0.8)
South Asia	149 000 000 (132 000 000–168 000 000)	8540 (7570–9640)	21 700 (16 500–26 300)	1.8 (1.4–2.2)
South Asia	149 000 000 (132 000 000–168 000 000)	8540 (7570–9640)	21 700 (16 500–26 300)	1.8 (1.4–2.2)
Bangladesh	16 600 000 (14 800 000–18 700 000)	10 600 (9480–11 900)	1490 (1050–2180)	1.3 (0.9–1.9)
Bhutan	61 200 (53 600–70 100)	8280 (7290–9460)	9.1 (5.29–13.1)	1.9 (1.1–2.8)
India	118 000 000 (105 000 000–134 000 000)	8520 (7520–9630)	18 000 (13 500–21 900)	1.9 (1.4–2.3)
Nepal	2 290 000 (2 010 000–2 580 000)	8250 (7190–9270)	318 (224–428)	1.8 (1.2–2.5)
Pakistan	11 200 000 (9 910 000–12 800 000)	6840 (6020–7770)	1910 (1240–2670)	2.2 (1.4–3.1)
Southeast Asia, east Asia, and Oceania	127 000 000 (108 000 000–146 000 000)	4740 (4100–5460)	15 300 (12 800–18 400)	0.6 (0.5–0.8)
East Asia	87 200 000 (74 200 000–100 000 000)	4480 (3860–5170)	10 300 (8430–13 100)	0.6 (0.5–0.7)
China	84 000 000 (71 300 000–96 700 000)	4470 (3840–5150)	9640 (7780–12 400)	0.6 (0.5–0.7)
North Korea	1 450 000 (1 260 000–1 640 000)	4510 (3930–5090)	228 (163–310)	0.8 (0.5–1.0)
Taiwan (province of China)	1 810 000 (1 590 000–2 090 000)	5400 (4710–6200)	409 (341–475)	1.1 (0.9–1.3)
Oceania	434 000 (376 000–508 000)	4010 (3470–4710)	38.5 (16.1–58)	0.4 (0.2–0.5)
American Samoa	2560 (2200–2960)	4880 (4230–5650)	0.512 (0.349–0.827)	1.1 (0.7–1.7)

Cook Islands	1080<br>(935–1250)	5070<br>(4380–5860)	0.00119<br>(0.001–0.00158)	0.0<br>(0.0–0.0)
Fiji	43 800<br>(38 200–50 400)	4930<br>(4350–5630)	1.21<br>(0.894–1.64)	0.2<br>(0.1–0.2)
Guam	9570<br>(8260–11 100)	5230<br>(4550–6000)	1.77<br>(1.53–2.04)	1.0<br>(0.8–1.1)
Kiribati	4480<br>(3930–5190)	4630<br>(4110–5330)	0.0599<br>(0.0371–0.0756)	0.1<br>(0.0–0.1)
Marshall Islands	2200<br>(1890–2570)	4390<br>(3790–5100)	0.234<br>(0.105–0.339)	0.5<br>(0.2–0.7)
Micronesia (Federated States of)	4020<br>(3410–4730)	4390<br>(3720–5160)	0.468<br>(0.208–0.699)	0.6<br>(0.2–0.8)
Nauru	350<br>(291–411)	4460<br>(3830–5130)	0.0502<br>(0.0206–0.076)	0.6<br>(0.3–0.9)
Niue	94.7<br>(82.4–108)	4920<br>(4300–5640)	0.0073<br>(0.00305–0.0101)	0.4<br>(0.2–0.6)
Northern Mariana Islands	2620<br>(2170–3070)	4810<br>(4100–5500)	0.148<br>(0.108–0.194)	0.4<br>(0.3–0.5)
Palau	1030<br>(871–1220)	4420<br>(3770–5140)	0.0222<br>(0.0168–0.0278)	0.1<br>(0.1–0.2)
Papua New Guinea	301 000<br>(260 000–354 000)	3790<br>(3250–4490)	26.8<br>(8.58–43.9)	0.3<br>(0.1–0.5)
Samoa	8110<br>(7050–9510)	4570<br>(3960–5330)	0.79<br>(0.348–1.25)	0.4<br>(0.2–0.7)
Solomon Islands	18 100<br>(15 500–20 900)	3540<br>(3040–4120)	2<br>(0.8–3.08)	0.4<br>(0.2–0.6)
Tokelau	65.8<br>(58–75.9)	4740<br>(4170–5480)	0.0051<br>(0.00209–0.00718)	0.4<br>(0.2–0.5)
Tonga	4250<br>(3690–4840)	4920<br>(4270–5590)	1.54<br>(1.21–1.91)	1.8<br>(1.4–2.2)
Tuvalu	510<br>(444–586)	4470<br>(3910–5120)	0.0456<br>(0.0204–0.0662)	0.4<br>(0.2–0.6)
Vanuatu	9970<br>(8590–11 600)	4270<br>(3700–4980)	0.998<br>(0.331–1.64)	0.4<br>(0.1–0.7)
Southeast Asia	39 100 000<br>(34 200 000–45 200 000)	5420<br>(4740–6230)	4950<br>(3970–5640)	0.8<br>(0.6–0.9)
Cambodia	737 000<br>(623 000–869 000)	4650<br>(3960–5500)	151<br>(73.7–231)	1.1<br>(0.5–1.6)
Indonesia	15 200 000<br>(13 300 000–17 600 000)	5520<br>(4850–6340)	663<br>(339–830)	0.3<br>(0.1–0.4)
Laos	330 000<br>(290 000–377 000)	5190<br>(4560–5840)	65.9<br>(34.1–96)	1.1<br>(0.5–1.5)
Malaysia	1 920 000<br>(1 690 000–2 200 000)	5840<br>(5200–6680)	339<br>(287–399)	1.2<br>(1.0–1.4)
Maldives	30 000<br>(25 700–34 400)	5400<br>(4690–6180)	1.84<br>(1.25–2.8)	0.5<br>(0.3–0.6)
Mauritius	98 700<br>(86 700–113 000)	6030<br>(5370–6850)	25.1<br>(21.1–28.8)	1.6<br>(1.4–1.9)
Myanmar	2 790 000<br>(2 410 000–3 300 000)	5000<br>(4320–5890)	514<br>(294–626)	1.0<br>(0.6–1.2)
Philippines	4 600 000<br>(4 000 000–5 320 000)	4550<br>(3970–5250)	1010<br>(857–1200)	1.1<br>(0.9–1.2)

Seychelles	7170<br>(6210–8160)	5950<br>(5200–6720)	0.159<br>(0.116–0.19)	0.1<br>(0.1–0.2)
Sri Lanka	1 330 000<br>(1 120 000–1 550 000)	5270<br>(4460–6090)	36.7<br>(26.3–48.2)	0.2<br>(0.1–0.2)
Thailand	6 610 000<br>(5 750 000–7 590 000)	7030<br>(6100–8070)	1490<br>(1190–1860)	1.7<br>(1.3–2.1)
Timor-Leste	47 200<br>(38 700–56 600)	4630<br>(3790–5540)	9.3<br>(3.96–17.7)	0.9<br>(0.4–1.6)
Viet Nam	5 410 000<br>(4 760 000–6 350 000)	4840<br>(4270–5650)	638<br>(319–893)	0.7<br>(0.3–1.0)
Sub-Saharan Africa	27 100 000<br>(23 300 000–31 600 000)	3770<br>(3250–4380)	4810<br>(3490–6010)	0.8<br>(0.6–1.1)
Central sub-Saharan Africa	3 050 000<br>(2 590 000–3 670 000)	3570<br>(3050–4220)	619<br>(311–971)	1.0<br>(0.5–1.6)
Angola	637 000<br>(501 000–776 000)	3450<br>(2850–4200)	132<br>(63.4–223)	0.9<br>(0.4–1.6)
Central African Republic	109 000<br>(91 300–131 000)	3240<br>(2790–3850)	29.3<br>(14.7–47.5)	1.1<br>(0.6–1.8)
Congo	159 000<br>(135 000–189 000)	3900<br>(3320–4520)	40<br>(21.5–61.8)	1.3<br>(0.7–2.0)
Democratic Republic of the Congo	2 040 000<br>(1 730 000–2 440 000)	3570<br>(3090–4180)	395<br>(181–661)	0.9<br>(0.4–1.7)
Equatorial Guinea	41 800<br>(36 300–48 700)	4520<br>(4010–5130)	7.73<br>(3.91–13.1)	1.2<br>(0.6–2.0)
Gabon	64 400<br>(55 900–74 300)	4360<br>(3800–5040)	15.3<br>(8.63–22.6)	1.4<br>(0.8–2.1)
Eastern sub-Saharan Africa	9 420 000<br>(7 890 000–11 200 000)	3560<br>(2990–4160)	1080<br>(638–1760)	0.5<br>(0.3–0.9)
Burundi	162 000<br>(116 000–219 000)	2230<br>(1550–2980)	30.1<br>(15.8–53)	0.5<br>(0.3–1.0)
Comoros	24 800<br>(21 700–29 000)	4020<br>(3500–4670)	3.08<br>(1.79–5.4)	0.6<br>(0.3–1.1)
Djibouti	38 700<br>(33 600–45 700)	3860<br>(3380–4480)	3.11<br>(1.44–6.18)	0.5<br>(0.2–1.0)
Eritrea	113 000<br>(59 200–161 000)	2410<br>(1280–3360)	18.4<br>(8.16–31.8)	0.6<br>(0.3–1.1)
Ethiopia	2 320 000<br>(1 900 000–2 770 000)	3450<br>(2860–4070)	232<br>(132–481)	0.5<br>(0.3–0.9)
Kenya	1 430 000<br>(1 240 000–1 660 000)	4020<br>(3490–4630)	134<br>(86.3–215)	0.6<br>(0.3–0.9)
Madagascar	717 000<br>(613 000–832 000)	3940<br>(3380–4490)	62.9<br>(35.9–109)	0.4<br>(0.2–0.8)
Malawi	464 000<br>(404 000–545 000)	3910<br>(3420–4510)	63.1<br>(34.8–105)	0.7<br>(0.4–1.2)
Mozambique	593 000<br>(489 000–720 000)	3350<br>(2770–4030)	100<br>(51.7–195)	0.7<br>(0.4–1.4)
Rwanda	207 000<br>(114 000–305 000)	2220<br>(1280–3200)	38<br>(21.5–70.6)	0.6<br>(0.3–1.1)
Somalia	317 000<br>(252 000–396 000)	2770<br>(2300–3340)	57.6<br>(31.3–85.2)	0.7<br>(0.4–1.1)
South Sudan	182 000<br>(148 000–218 000)	3210<br>(2700–3780)	32.3<br>(18.2–49.4)	0.7<br>(0.4–1.1)

Uganda	877 000<br>(736 000–1 040 000)	3630<br>(3100–4300)	97.5<br>(56–176)	0.5<br>(0.3–1.0)
Tanzania	1 480 000<br>(1 280 000–1 720 000)	3920<br>(3400–4510)	157<br>(92.6–267)	0.5<br>(0.3–0.9)
Zambia	484 000<br>(412 000–554 000)	4030<br>(3520–4590)	53.9<br>(33.6–78.9)	0.6<br>(0.4–0.9)
Southern sub-Saharan Africa	3 010 000<br>(2 610 000–3 500 000)	4210<br>(3650–4860)	822<br>(679–953)	1.4<br>(1.1–1.6)
Botswana	88 800<br>(76 600–104 000)	4270<br>(3720–4910)	12.3<br>(7.45–17.5)	0.8<br>(0.5–1.2)
Eswatini	35 400<br>(30 400–41 400)	4100<br>(3540–4650)	10.9<br>(6.34–17.9)	1.7<br>(1.0–2.6)
Lesotho	65 300<br>(56 000–75 800)	3750<br>(3170–4340)	19.8<br>(13.4–28.6)	1.5<br>(1.0–2.1)
Namibia	80 100<br>(68 900–94 200)	4150<br>(3620–4810)	13.7<br>(7.82–22.2)	0.9<br>(0.5–1.5)
South Africa	2 320 000<br>(2 010 000–2 690 000)	4240<br>(3660–4900)	690<br>(565–806)	1.5<br>(1.2–1.7)
Zimbabwe	425 000<br>(369 000–496 000)	3990<br>(3470–4620)	75.3<br>(31.5–105)	0.9<br>(0.4–1.3)
Western sub-Saharan Africa	11 600 000<br>(10 100 000–13 500 000)	3910<br>(3390–4500)	2290<br>(1640–2810)	0.9<br>(0.7–1.1)
Benin	298 000<br>(256 000–342 000)	3730<br>(3210–4260)	64.9<br>(45.2–88.3)	0.9<br>(0.7–1.2)
Burkina Faso	508 000<br>(430 000–586 000)	3490<br>(3010–4100)	138<br>(91.7–182)	1.1<br>(0.7–1.3)
Cabo Verde	21 800<br>(19 200–25 200)	4050<br>(3510–4680)	2.52<br>(1.92–3.26)	0.6<br>(0.4–0.7)
Cameroon	792 000<br>(680 000–936 000)	3970<br>(3450–4620)	184<br>(119–260)	1.1<br>(0.8–1.5)
Chad	293 000<br>(247 000–347 000)	3210<br>(2720–3760)	63.5<br>(43.9–99.2)	0.7<br>(0.5–1.1)
Côte d’Ivoire	695 000<br>(596 000–812 000)	3780<br>(3220–4450)	122<br>(82.5–166)	0.8<br>(0.6–1.1)
Gambia	58 700<br>(50 700–67 500)	3850<br>(3360–4370)	11.3<br>(7.79–15.4)	0.9<br>(0.6–1.2)
Ghana	1 060 000<br>(923 000–1 230 000)	4280<br>(3760–4930)	184<br>(135–244)	1.1<br>(0.8–1.4)
Guinea	294 000<br>(257 000–342 000)	3620<br>(3140–4160)	59.7<br>(38.7–89.6)	0.8<br>(0.5–1.1)
Guinea-Bissau	45 500<br>(39 000–53 300)	3600<br>(3140–4200)	11.7<br>(7.81–16.3)	1.2<br>(0.8–1.6)
Liberia	117 000<br>(96 800–141 000)	3440<br>(2970–4110)	30.4<br>(19–41)	1.0<br>(0.7–1.3)
Mali	435 000<br>(371 000–514 000)	3300<br>(2820–3870)	122<br>(83.4–169)	1.0<br>(0.7–1.3)
Mauritania	117 000<br>(101 000–135 000)	4030<br>(3460–4660)	19.3<br>(13.1–26.2)	0.8<br>(0.6–1.1)
Niger	403 000<br>(345 000–468 000)	3180<br>(2730–3740)	91.9<br>(63.4–130)	0.8<br>(0.6–1.1)
Nigeria	5 650 000<br>(4 920 000–6 560 000)	4110<br>(3570–4730)	1 020<br>(650–1340)	0.9<br>(0.6–1.1)

São Tomé and Príncipe	6260<br>(5460–7430)	3860<br>(3350–4510)	1.07<br>(0.736–1.43)	0.9<br>(0.6–1.1)
Senegal	419 000<br>(367 000–485 000)	3880<br>(3400–4520)	79.9<br>(58.1–106)	0.9<br>(0.6–1.2)
Sierra Leone	197 000<br>(165 000–237 000)	3500<br>(2950–4100)	42.4<br>(28.5–60.2)	0.8<br>(0.6–1.1)
Togo	223 000<br>(194 000–264 000)	3840<br>(3310–4460)	41<br>(29.4–54.8)	0.9<br>(0.7–1.2)

Supplemental Table 7: Counts and age-standardised rates of YLDs, YLLs, and DALYs in 2020 for other MSK disorders, by region and by country

Location	Number of YLDs (95% UI)	Age-standardised rate of YLDs per 100 000 in 2020 (95% UI)	Number of YLLs (95% UI)	Age-standardised rate of YLLs per 100 000 (95% UI)	Number of DALYs (95% UI)	Age-standardised rate of DALYs per 100 000 (95% UI)
Global	42 700 000 (29 400 000–60 000 000)	512·0 (352·0–717·0)	2 210 000 (1 920 000–2 420 000)	27.2 (23.6–29.7)	44 900 000 (31 600 000–62 200 000)	539.0 (380.0–744.0)
Central Europe, eastern Europe, and central Asia	911 000 (630 000–1 290 000)	174·0 (120·0–244·0)	109 000 (102 000–120 000)	20.4 (19.1–22.5)	1 020 000 (739 000–1 400 000)	194.0 (141.0–265.0)
Central Asia	190 000 (128 000–263 000)	207·0 (141·0–281·0)	10 200 (8890–14 300)	12.6 (11.2–16.5)	200 000 (139 000–276 000)	219.0 (154.0–294.0)
Armenia	8210 (5460–11 400)	219·0 (147·0–304·0)	229 (207–279)	6.2 (5.6–7.7)	8440 (5690–11 700)	226.0 (153.0–310.0)
Azerbaijan	26 000 (17 400–37 000)	228·0 (155·0–320·0)	54.5 (13.1–82.7)	0.6 (0.1–0.9)	26 000 (17 400–37 100)	229.0 (155.0–321.0)
Georgia	7920 (5310–11 500)	170·0 (116·0–236·0)	398 (360–437)	7.1 (6.4–7.7)	8320 (5730–11 900)	177.0 (124.0–243.0)
Kazakhstan	37 000 (25 200–52 200)	194·0 (133·0–265·0)	2780 (2320–6870)	15.8 (13.5–35.5)	39 800 (27 900–57 800)	209.0 (148.0–294.0)
Kyrgyzstan	12 600 (8370–17 500)	213·0 (144·0–293·0)	1920 (1620–2210)	34.9 (29.4–40.0)	14 500 (10 200–19 300)	248.0 (178.0–326.0)
Mongolia	5250 (3530–7400)	166·0 (113·0–224·0)	774 (562–1090)	28.4 (20.3–40.6)	6030 (4330–8330)	195.0 (144.0–257.0)
Tajikistan	15 100 (9830–21 400)	193·0 (130·0–267·0)	222 (93.2–327)	3.3 (1.5–4.9)	15 300 (10 000–21 700)	197.0 (133.0–271.0)
Turkmenistan	10 900 (7410–15 200)	227·0 (154·0–310·0)	253 (207–301)	5.5 (4.5–6.6)	11 200 (7670–15 500)	233.0 (160.0–316.0)
Uzbekistan	66 600 (44 800–91 700)	218·0 (148·0–296·0)	3580 (3040–4270)	15.1 (12.6–18.2)	70 200 (48 300–95 500)	234.0 (163.0–311.0)
Central Europe	262 000 (181 000–368 000)	179·0 (123·0–248·0)	22 700 (20 200–26 700)	13.4 (11.9–16.2)	285 000 (203 000–391 000)	193.0 (137.0–262.0)

Albania	5540 (3700–7650)	157·0 (108·0–214·0)	660 (382–1290)	19.5 (11.1–38.7)	6200 (4200–8580)	177.0 (121.0–242.0)
Bosnia and Herzegovina	7650 (5180–11 100)	170·0 (114·0–238·0)	679 (445–964)	14.3 (9.4–20.7)	8330 (5680–12 000)	184.0 (125.0–254.0)
Bulgaria	15 500 (10 600–22 800)	162·0 (110·0–224·0)	614 (537–720)	6.0 (5.3–7.3)	16 200 (11 200–23 300)	168.0 (116.0–229.0)
Croatia	9350 (6440–13 400)	174·0 (117·0–247·0)	1090 (955–1290)	15.3 (13.4–18.8)	10 400 (7480–14 500)	190.0 (133.0–263.0)
Czechia	22 800 (15 400–34 100)	170·0 (114·0–241·0)	1660 (1390–1950)	9.6 (8.0–11.3)	24 500 (17 200–35 800)	179.0 (124.0–251.0)
Hungary	22 200 (15 100–31 700)	179·0 (123·0–254·0)	3560 (2940–4270)	22.9 (18.9–27.5)	25 800 (18 600–35 500)	202.0 (145.0–277.0)
Montenegro	1390 (927–2000)	177·0 (119·0–256·0)	51.8 (37.5–67.7)	6.5 (4.7–8.5)	1440 (975–2050)	183.0 (125.0–263.0)
North Macedonia	4980 (3470–6960)	179·0 (127·0–256·0)	277 (197–380)	9.6 (6.8–13.3)	5250 (3720–7310)	189.0 (136.0–268.0)
Poland	94 700 (64 900–130 000)	198·0 (136·0–272·0)	7820 (7020–10 200)	14.3 (12.8–19.0)	103 000 (73 400–138 000)	212.0 (151.0–287.0)
Romania	42 000 (28 700–62 700)	167·0 (113·0–243·0)	2500 (2060–2900)	8.8 (7.3–10.2)	44 500 (31 300–65 000)	176.0 (123.0–253.0)
Serbia	21 000 (14 800–30 600)	186·0 (129·0–260·0)	2420 (1950–3090)	19.0 (15.4–25.1)	23 400 (17 200–33 100)	205.0 (147.0–281.0)
Slovakia	11 000 (7590–15 600)	161·0 (113·0–227·0)	699 (526–901)	9.2 (6.9–11.9)	11 700 (8270–16 400)	170.0 (122.0–236.0)
Slovenia	3910 (2610–5770)	152·0 (105·0–215·0)	625 (514–831)	17.3 (14.2–23.8)	4540 (3290–6500)	169.0 (123.0–235.0)
Eastern Europe	459 000 (319 000–661 000)	161·0 (113·0–232·0)	76 500 (70 200–82 800)	27.2 (25.0–29.3)	536 000 (397 000–739 000)	188.0 (140.0–260.0)
Belarus	20 500 (13 900–30 300)	165·0 (111·0–238·0)	133 (115–152)	1.1 (0.9–1.2)	20 600 (14 100–30 400)	166.0 (112.0–239.0)
Estonia	3500 (2410–4990)	199·0 (137·0–280·0)	679 (570–798)	35.8 (30.1–43.0)	4180 (3060–5660)	235.0 (172.0–317.0)
Latvia	4800 (3160–6800)	186·0 (125·0–264·0)	776 (669–928)	28.7 (24.5–34.7)	5570 (3920–7590)	215.0 (154.0–292.0)

Lithuania	6710 (4490–9490)	182·0 (125·0–257·0)	1360 (1130–1600)	34.3 (28.5–41.2)	8070 (5910–10 800)	217.0 (161.0–292.0)
Republic of Moldova	9510 (6330–13 700)	194·0 (129·0–277·0)	1030 (893–1190)	21.2 (18.3–24.5)	10 500 (7400–14 700)	215.0 (150.0–298.0)
Russia	315 000 (217 000–463 000)	160·0 (111·0–235·0)	64 000 (58 500–69 900)	32.5 (29.7–35.4)	379 000 (281 000–529 000)	192.0 (143.0–269.0)
Ukraine	98 800 (68 400–138 000)	157·0 (110·0–212·0)	8560 (6560–10 700)	15.7 (12.1–19.6)	107 000 (77 000–147 000)	173.0 (125.0–228.0)
High income	9 090 000 (6 190 000–12 200 000)	671·0 (458·0–901·0)	454 000 (416 000–476 000)	26.2 (24.6–27.4)	9 550 000 (6 640 000–12 600 000)	697.0 (484.0–927.0)
Australasia	247 000 (175 000–337 000)	649·0 (457·0–871·0)	14 300 (12 900–15 500)	31.5 (29.0–34.1)	262 000 (189 000–352 000)	680.0 (488.0–904.0)
Australia	228 000 (162 000–308 000)	717·0 (503·0–959·0)	11 800 (10 600–12 900)	30.7 (28.1–33.5)	240 000 (174 000–319 000)	748.0 (534.0–991.0)
New Zealand	19 300 (12 600–28 200)	307·0 (200·0–445·0)	2470 (2260–2740)	35.0 (32.5–38.5)	21 800 (15 000–30 700)	342.0 (234.0–483.0)
High income Asia Pacific	1 920 000 (1 310 000–2 560 000)	720·0 (495·0–980·0)	73 500 (63 100–80 800)	21.0 (18.9–22.9)	1 990 000 (1 380 000–2 640 000)	741.0 (515.0–1000.0)
Brunei	3130 (2160–4430)	658·0 (454·0–905·0)	153 (121–193)	38.4 (30.5–49.2)	3290 (2310–4610)	696.0 (492.0–948.0)
Japan	1 370 000 (941 000–1 820 000)	735·0 (506·0–1010·0)	53 700 (46 500–57 500)	20.6 (18.9–21.6)	1 420 000 (996 000–1 870 000)	755.0 (527.0–1030.0)
South Korea	492 000 (333 000–678 000)	675·0 (459·0–921·0)	18 600 (14 600–23 600)	23.9 (18.8–30.4)	510 000 (349 000–695 000)	699.0 (481.0–943.0)
Singapore	55 600 (38 700–75 800)	727·0 (503·0–979·0)	1100 (1000–1190)	14.6 (13.4–15.7)	56 700 (39 800–77 000)	743.0 (518.0–995.0)
High income North America	4 180 000 (2 850 000–5 560 000)	951·0 (651·0–1270·0)	166 000 (156 000–174 000)	33.1 (31.6–34.5)	4 340 000 (3 020 000–5 730 000)	984.0 (684.0–1300.0)
Canada	445 000 (304 000–603 000)	975·0 (664·0–1340·0)	17 500 (16 100–18 900)	30.7 (28.8–32.9)	462 000 (321 000–620 000)	1010.0 (695.0–1370.0)

Greenland	579 (395–801)	883·0 (612·0–1210·0)	27.9 (18.8–39.8)	43.2 (29.5–62.0)	607 (423–837)	927.0 (656.0–1260.0)
USA	3 760 000 (2 570 000–5 000 000)	948·0 (649·0–1260·0)	148 000 (140 000–155 000)	33.2 (31.7–34.5)	3 910 000 (2 720 000–5 150 000)	981.0 (682.0–1300.0)
Southern Latin America	692 000 (486 000–959 000)	908·0 (639·0–1260·0)	22 200 (20 700–23 600)	29.1 (27.3–31.0)	714 000 (509 000–981 000)	937.0 (668.0–1290.0)
Argentina	460 000 (325 000–641 000)	915·0 (645·0–1280·0)	14 800 (13 800–15 900)	29.6 (27.6–31.8)	474 000 (340 000–656 000)	944.0 (675.0–1310.0)
Chile	197 000 (137 000–272 000)	901·0 (630·0–1250·0)	6090 (5620–6800)	27.7 (25.7–30.8)	203 000 (143 000–279 000)	929.0 (659.0–1280.0)
Uruguay	35 500 (24 500–48 000)	870·0 (599·0–1190·0)	1290 (1200–1390)	30.8 (28.7–32.9)	36 800 (25 800–49 300)	901.0 (631.0–1220.0)
Western Europe	2 060 000 (1 380 000–2 860 000)	376·0 (254·0–529·0)	178 000 (162 000–190 000)	21.5 (20.0–23.8)	2 240 000 (1 560 000–3 060 000)	397.0 (276.0–553.0)
Andorra	353 (229–522)	320·0 (212·0–449·0)	2.88 (2.1–3.63)	2.2 (1.6–2.7)	356 (232–525)	322.0 (214.0–452.0)
Austria	39 200 (26 000–57 200)	351·0 (238·0–507·0)	2500 (2200–2750)	15.0 (13.5–16.6)	41 700 (28 600–59 800)	366.0 (254.0–523.0)
Belgium	42 100 (28 800–59 700)	320·0 (214·0–451·0)	3980 (3450–4530)	19.0 (17.0–23.2)	46 000 (32 900–64 200)	339.0 (233.0–474.0)
Cyprus	6010 (4010–8590)	360·0 (239·0–510·0)	426 (314–546)	25.2 (19.1–33.2)	6430 (4440–9090)	385.0 (266.0–538.0)
Denmark	29 800 (20 200–41 800)	405·0 (279·0–568·0)	2430 (2110–2670)	22.0 (19.5–24.0)	32 200 (22 800–44 400)	427.0 (301.0–591.0)
Finland	20 500 (13 100–29 800)	320·0 (211·0–461·0)	1550 (1340–1690)	15.8 (14.3–17.1)	22 000 (14 700–31 300)	336.0 (228.0–477.0)
France	254 000 (170 000–366 000)	334·0 (222·0–472·0)	39 800 (35 300–43 800)	29.9 (27.3–32.5)	294 000 (211 000–407 000)	364.0 (253.0–505.0)

Germany	419 000 (276 000–593 000)	387·0 (258·0–551·0)	30 400 (26 600–33 700)	17.7 (15.8–19.2)	449 000 (307 000–626 000)	405.0 (276.0–570.0)
Greece	51 200 (33 900–72 400)	371·0 (246·0–534·0)	2290 (2070–2520)	10.6 (9.8–11.6)	53 500 (36 300–74 800)	381.0 (257.0–545.0)
Iceland	1590 (1070–2280)	384·0 (259·0–544·0)	87 (75.4–98.6)	15.9 (14.2–17.9)	1680 (1160–2370)	400.0 (276.0–561.0)
Ireland	22 800 (15 500–31 400)	388·0 (261·0–534·0)	1710 (1560–1930)	24.3 (22.3–27.6)	24 500 (17 200–33 400)	412.0 (285.0–561.0)
Israel	38 500 (26 500–53 200)	391·0 (266·0–544·0)	1870 (1710–2030)	16.3 (15.1–17.7)	40 400 (28 500–55 200)	407.0 (283.0–562.0)
Italy	323 000 (217 000–456 000)	399·0 (273·0–566·0)	21 000 (18 300–22 500)	18.2 (16.7–19.3)	344 000 (237 000–478 000)	418.0 (291.0–585.0)
Luxembourg	2840 (1920–4100)	361·0 (247·0–511·0)	131 (118–145)	13.2 (11.9–14.5)	2970 (2050–4230)	374.0 (260.0–524.0)
Malta	2060 (1380–2910)	359·0 (240·0–501·0)	131 (116–145)	16.4 (14.6–19.3)	2190 (1520–3040)	375.0 (258.0–518.0)
Monaco	214 (147–303)	410·0 (283·0–582·0)	7.31 (5.56–9.43)	8.8 (6.7–11.3)	222 (153–311)	419.0 (293.0–592.0)
Netherlands	86 400 (56 900–123 000)	404·0 (271·0–572·0)	7460 (6640–8110)	24.0 (21.6–26.3)	93 900 (64 600–131 000)	428.0 (296.0–598.0)
Norway	19 700 (13 300–28 000)	301·0 (205·0–431·0)	2130 (1890–2290)	22.9 (20.6–24.4)	21 900 (15 400–30 200)	324.0 (229.0–455.0)
Portugal	52 800 (36 600–72 900)	376·0 (260·0–515·0)	2790 (2460–3120)	13.6 (12.3–16.2)	55 600 (39 600–76 000)	389.0 (273.0–531.0)
San Marino	163 (109–230)	390·0 (264·0–545·0)	5.39 (3.99–7.28)	8.8 (6.5–11.8)	168 (114–236)	399.0 (272.0–554.0)
Spain	218 000 (151 000–302 000)	365·0 (251·0–505·0)	19 100 (16 800–21 900)	21.7 (19.7–27.0)	237 000 (171 000–321 000)	387.0 (273.0–531.0)
Sweden	50 200 (33 900–70 400)	397·0 (270·0–554·0)	3540 (3170–3790)	19.0 (17.7–20.2)	53 700 (37 400–73 800)	416.0 (289.0–573.0)
Switzerland	39 900 (26 600–59 000)	364·0 (244·0–532·0)	3040 (2490–3390)	17.0 (14.4–19.7)	42 900 (29 800–62 300)	381.0 (261.0–551.0)
UK	336 000 (228 000–467 000)	398·0 (272·0–558·0)	31 800 (28 800–36 100)	27.7 (25.6–34.7)	368 000 (259 000–500 000)	426.0 (299.0–589.0)

Latin America and Caribbean	4 040 000 (2 800 000–5 740 000)	642·0 (444·0–907·0)	298 000 (277 000–324 000)	48.4 (45.0–52.6)	4 340 000 (3 100 000–6 040 000)	690.0 (494.0–955.0)
Andean Latin America	354 000 (245 000–504 000)	561·0 (387·0–795·0)	15 500 (12 800–20 400)	24.4 (20.1–31.8)	369 000 (260 000–519 000)	586.0 (411.0–819.0)
Bolivia	59 100 (40 500–82 500)	543·0 (372·0–756·0)	3130 (2110–4290)	28.8 (19.5–38.6)	62 200 (43 800–85 600)	572.0 (402.0–786.0)
Ecuador	94 500 (65 600–133 000)	549·0 (381·0–774·0)	5850 (4680–11 400)	33.9 (27.2–66.0)	100 000 (71 100–140 000)	583.0 (413.0–809.0)
Peru	200 000 (138 000–284 000)	572·0 (395·0–808·0)	6570 (4920–8510)	18.7 (14.0–24.2)	207 000 (145 000–290 000)	591.0 (413.0–825.0)
Caribbean	221 000 (154 000–307 000)	431·0 (302·0–597·0)	27 600 (22 200–36 400)	55.2 (44.4–73.4)	249 000 (186 000–333 000)	486.0 (362.0–650.0)
Antigua and Barbuda	502 (344–704)	471·0 (325·0–653·0)	56 (49–63)	56.3 (49.6–63.1)	558 (404–760)	527.0 (385.0–709.0)
Bahamas	2140 (1470–3040)	493·0 (342·0–691·0)	446 (358–557)	108.0 (87.1–134.0)	2590 (1920–3500)	601.0 (452.0–800.0)
Barbados	1960 (1360–2680)	492·0 (341·0–682·0)	315 (257–381)	86.0 (69.3–105.0)	2270 (1700–3020)	578.0 (434.0–767.0)
Belize	1600 (1070–2240)	425·0 (288·0–591·0)	244 (206–355)	59.7 (50.5–84.1)	1840 (1320–2550)	485.0 (348.0–665.0)
Bermuda	480 (325–668)	516·0 (355·0–723·0)	33.8 (28–43.6)	35.5 (28.0–64.8)	513 (359–700)	551.0 (389.0–759.0)
Cuba	70 600 (49 100–98 000)	471·0 (331·0–646·0)	5840 (4980–6730)	41.1 (34.9–47.4)	76 400 (55 000–104 000)	512.0 (371.0–690.0)
Dominica	388 (266–546)	482·0 (331·0–682·0)	25.5 (20.5–31.3)	33.5 (26.8–41.3)	413 (293–573)	516.0 (367.0–717.0)
Dominican Republic	46 400 (32 400–64 600)	433·0 (301·0–601·0)	3880 (2910–5200)	35.8 (26.8–47.9)	50 300 (35 300–69 200)	468.0 (328.0–643.0)
Grenada	525 (355–733)	452·0 (310·0–629·0)	66.9 (58.4–76.5)	63.2 (55.9–72.0)	592 (424–807)	516.0 (375.0–699.0)

Guyana	3190 (2210–4650)	421·0 (292·0–610·0)	278 (208–356)	36.0 (27.3–45.7)	3470 (2460–4900)	457.0 (324.0–643.0)
Haiti	35 200 (23 800–52 100)	337·0 (232·0–490·0)	9570 (5960–17 400)	80.5 (49.9–141.0)	44 800 (34 100–60 400)	417.0 (318.0–559.0)
Jamaica	14 400 (10 200–20 100)	469·0 (334·0–655·0)	2 790 (2 200–3 660)	89.4 (70.3–118.0)	17 200 (12 800–22 800)	558.0 (418.0–742.0)
Puerto Rico	22 800 (15 500–31 200)	486·0 (334·0–676·0)	1340 (1140–1550)	29.7 (25.2–34.9)	24 200 (16 900–32 500)	516.0 (365.0–706.0)
Saint Kitts and Nevis	340 (237–484)	466·0 (328·0–655·0)	47.6 (39.5–55.8)	73.6 (61.7–85.3)	387 (283–530)	540.0 (397.0–727.0)
Saint Lucia	981 (664–1370)	458·0 (311·0–637·0)	113 (94.4–137)	56.2 (47.1–67.8)	1090 (778–1490)	514.0 (370.0–690.0)
Saint Vincent and the Grenadines	578 (403–816)	442·0 (311·0–620·0)	78.2 (69.6–88.4)	64.5 (57.7–72.7)	657 (483–892)	506.0 (376.0–683.0)
Suriname	2790 (1920–3920)	445·0 (309·0–620·0)	386 (326–451)	64.5 (54.6–75.7)	3170 (2310–4290)	510.0 (374.0–682.0)
Trinidad and Tobago	8490 (5940–11 800)	489·0 (342·0–678·0)	1070 (825–1350)	68.3 (52.8–85.9)	9560 (7140–12 800)	557.0 (417.0–746.0)
Virgin Islands	684 (483–946)	499·0 (356·0–707·0)	57.1 (44.4–79)	44.8 (34.3–64.8)	741 (529–1000)	544.0 (392.0–750.0)
Central Latin America	1 870 000 (1 290 000–2 660 000)	723·0 (497·0–1020·0)	149 000 (126 000–167 000)	57.4 (48.8–64.3)	2 020 000 (1 440 000–2 810 000)	780.0 (555.0–1080.0)
Colombia	383 000 (264 000–552 000)	722·0 (498·0–1040·0)	36 700 (31 600–43 000)	71.1 (61.2–83.1)	420 000 (302 000–586 000)	793.0 (573.0–1110.0)
Costa Rica	39 200 (27 100–55 000)	736·0 (510·0–1030·0)	2690 (2320–3120)	51.9 (44.8–60.0)	41 900 (30 000–57 700)	788.0 (566.0–1080.0)
El Salvador	39 800 (27 600–58 100)	646·0 (447·0–947·0)	2310 (1950–2670)	36.4 (30.7–42.4)	42 100 (30 000–60 500)	683.0 (484.0–982.0)

Guatemala	93 900 (63 400–132 000)	621·0 (417·0–875·0)	4670 (3950–5260)	27.8 (23.5–31.3)	98 500 (68 200–137 000)	649.0 (446.0–902.0)
Honduras	54 200 (37 100–75 100)	659·0 (454·0–916·0)	5280 (3310–7460)	61.0 (40.8–86.0)	59 500 (43 000–80 100)	720.0 (525.0–973.0)
Mexico	981 000 (672 000–1 390 000)	750·0 (514·0–1050·0)	70 300 (52 800–80 500)	54.1 (40.7–61.8)	1 050 000 (740 000–1 460 000)	804.0 (566.0–1110.0)
Nicaragua	38 300 (26 800–54 600)	645·0 (453·0–902·0)	3440 (2780–4020)	54.7 (44.1–63.4)	41 700 (30 500–57 900)	699.0 (512.0–952.0)
Panama	32 400 (22 200–45 700)	752·0 (517·0–1060·0)	1670 (1430–1950)	38.7 (33.2–45.3)	34 100 (24 000–47 400)	791.0 (558.0–1100.0)
Venezuela	210 000 (146 000–297 000)	689·0 (478·0–963·0)	21 900 (17 000–28 700)	76.1 (59.0–99.4)	232 000 (170 000–319 000)	765.0 (561.0–1040.0)
Tropical Latin America	1 590 000 (1 110 000–2 270 000)	622·0 (433·0–881·0)	106 000 (102 000–117 000)	43.8 (41.9–47.8)	1 700 000 (1 220 000–2 380 000)	666.0 (478.0–924.0)
Brazil	1 550 000 (1 080 000–2 210 000)	622·0 (433·0–881·0)	102 000 (97 900–113 000)	43.4 (41.6–47.7)	1 650 000 (1 190 000–2 310 000)	665.0 (477.0–923.0)
Paraguay	42 200 (29 700–60 400)	630·0 (442·0–899·0)	3960 (2740–4930)	56.9 (39.5–70.7)	46 200 (33 700–64 200)	687.0 (500.0–955.0)
North Africa and Middle East	2 410 000 (1 660 000–3 540 000)	402·0 (280·0–579·0)	127 000 (104 000–155 000)	22.0 (17.8–26.6)	2 540 000 (1 790 000–3 660 000)	424.0 (303.0–600.0)
North Africa and Middle East	2 410 000 (1 660 000–3 540 000)	402·0 (280·0–579·0)	127 000 (104 000–155 000)	22.0 (17.8–26.6)	2 540 000 (1 790 000–3 660 000)	424.0 (303.0–600.0)
Afghanistan	48 700 (17 900–89 700)	199·0 (60·0–355·0)	15 100 (9 380–23 100)	46.2 (27.3–71.1)	63 800 (31 000–106 000)	245.0 (111.0–406.0)
Algeria	188 000 (131 000–275 000)	435·0 (301·0–625·0)	7100 (5160–8890)	17.3 (12.5–21.3)	195 000 (138 000–282 000)	452.0 (319.0–642.0)
Bahrain	8770 (6070–13 000)	465·0 (328·0–658·0)	486 (325–566)	42.0 (28.2–49.5)	9260 (6560–13 600)	508.0 (373.0–707.0)

Egypt	391 000 (275 000–563 000)	441·0 (312·0–626·0)	9890 (7130–11 600)	10.8 (8.0–12.5)	401 000 (286 000–572 000)	452.0 (324.0–636.0)
Iran	367 000 (256 000–539 000)	389·0 (271·0–557·0)	18 300 (12 900–20 800)	21.9 (15.0–24.8)	386 000 (275 000–558 000)	411.0 (293.0–580.0)
Iraq	114 000 (73 800–178 000)	313·0 (203·0–485·0)	6890 (5050–9510)	18.0 (13.5–24.3)	121 000 (80 700–185 000)	331.0 (221.0–503.0)
Jordan	50 000 (34 500–72 000)	472·0 (325·0–666·0)	2790 (2270–3490)	27.3 (22.5–33.9)	52 800 (37 300–75 300)	499.0 (353.0–699.0)
Kuwait	27 500 (18 800–41 200)	507·0 (354·0–727·0)	579 (502–879)	14.1 (12.4–18.5)	28 100 (19 300–41 800)	522.0 (369.0–740.0)
Lebanon	23 400 (15 600–35 000)	421·0 (280·0–630·0)	747 (577–901)	14.2 (11.0–17.0)	24 100 (16 300–35 800)	436.0 (293.0–644.0)
Libya	31 000 (20 500–47 900)	411·0 (277·0–614·0)	1710 (968–2560)	24.5 (14.5–35.8)	32 800 (22 900–49 100)	436.0 (305.0–638.0)
Morocco	153 000 (105 000–211 000)	405·0 (279·0–553·0)	10 400 (4680–19 700)	29.1 (13.3–54.4)	164 000 (116 000–221 000)	434.0 (309.0–579.0)
Oman	20 300 (13 700–29 700)	415·0 (293·0–600·0)	1050 (536–2300)	28.9 (17.7–50.6)	21 400 (14 300–31 000)	444.0 (315.0–631.0)
Palestine	12 900 (8 190–20 000)	332·0 (213·0–505·0)	388 (241–463)	10.0 (6.0–11.7)	13 300 (8570–20 300)	342.0 (224.0–515.0)
Qatar	14 900 (10 000–21 600)	428·0 (300·0–618·0)	106 (65.1–183)	5.2 (3.3–8.1)	15 000 (10 100–21 800)	433.0 (305.0–624.0)
Saudi Arabia	148 000 (102 000–232 000)	333·0 (234·0–508·0)	11 700 (8240–16 500)	29.0 (21.1–39.7)	160 000 (114 000–244 000)	362.0 (261.0–541.0)
Sudan	118 000 (79 300–173 000)	390·0 (265·0–560·0)	9070 (5700–13 900)	24.5 (16.0–37.9)	127 000 (89 000–182 000)	415.0 (293.0–580.0)
Syria	46 600 (30 000–73 200)	326·0 (210·0–501·0)	755 (540–1030)	5.6 (4.1–7.6)	47 300 (30 800–74 000)	331.0 (217.0–506.0)
Tunisia	60 100 (40 600–86 400)	446·0 (300·0–636·0)	2130 (1280–3850)	17.3 (10.5–31.1)	62 300 (42 300–88 700)	464.0 (316.0–654.0)
Türkiye	448 000 (319 000–633 000)	465·0 (330·0–651·0)	21 800 (17 300–27 900)	25.0 (19.8–31.7)	470 000 (341 000–653 000)	490.0 (356.0–675.0)
United Arab Emirates	54 600 (35 300–86 700)	427·0 (292·0–643·0)	1060 (827–1380)	17.0 (13.6–22.1)	55 700 (36 300–87 700)	444.0 (309.0–662.0)

Yemen	79 100 (51 600–117 000)	342·0 (228·0–489·0)	4540 (2510–7360)	17.4 (9.6–27.8)	83 600 (58 200–121 000)	360.0 (253.0–506.0)
South Asia	12 800 000 (8 880 000–18 100 000)	733·0 (507·0–1030·0)	471 000 (364 000–558 000)	33.4 (25.8–40.1)	13 300 000 (9 360 000–18 500 000)	767.0 (540.0–1060.0)
South Asia	12 800 000 (8 880 000–18 100 000)	733·0 (507·0–1030·0)	471 000 (364 000–558 000)	33.4 (25.8–40.1)	13 300 000 (9 360 000–18 500 000)	767.0 (540.0–1060.0)
Bangladesh	1 430 000 (993 000–1 990 000)	912·0 (631·0–1250·0)	30 900 (21 100–44 200)	23.2 (16.0–33.3)	1 460 000 (1 020 000–2 030 000)	935.0 (653.0–1290.0)
Bhutan	5340 (3780–7570)	717·0 (506·0–1010·0)	178 (107–259)	32.1 (19.0–46.4)	5510 (3960–7790)	749.0 (540.0–1050.0)
India	10 200 000 (7 080 000–14 500 000)	732·0 (506·0–1030·0)	382 000 (291 000–456 000)	33.7 (25.6–40.7)	10 600 000 (7 470 000–14 800 000)	766.0 (540.0–1060.0)
Nepal	197 000 (138 000–275 000)	707·0 (494·0–989·0)	6960 (5190–9300)	31.5 (22.9–42.3)	204 000 (146 000–283 000)	738.0 (528.0–1020.0)
Pakistan	966 000 (670 000–1 350 000)	582·0 (402·0–807·0)	50 500 (35 000–69 500)	41.6 (27.3–58.1)	1 020 000 (718 000–1 400 000)	624.0 (440.0–846.0)
Southeast Asia, east Asia, and Oceania	11 100 000 (7 570 000–16 000 000)	416·0 (286·0–594·0)	540 000 (451 000–640 000)	23.6 (19.5–27.9)	11 600 000 (8 110 000–16 500 000)	440.0 (310.0–617.0)
East Asia	7 630 000 (5 170 000–10 900 000)	394·0 (271·0–563·0)	331 000 (276 000–411 000)	20.5 (17.1–25.2)	7 960 000 (5 500 000–11 300 000)	415.0 (291.0–583.0)
China	7 340 000 (4 970 000–10 500 000)	393·0 (269·0–562·0)	311 000 (255 000–390 000)	19.9 (16.5–24.7)	7 650 000 (5 280 000–10 800 000)	413.0 (289.0–581.0)
North Korea	127 000 (86 200–176 000)	396·0 (272·0–540·0)	9520 (6820–12 800)	32.7 (23.4–43.6)	136 000 (96 000–187 000)	428.0 (305.0–583.0)

Taiwan (province of China)	158 000 (110 000–224 000)	474·0 (330·0–673·0)	11 000 (9330–12 900)	35.6 (30.5–41.4)	169 000 (121 000–234 000)	510.0 (368.0–707.0)
Oceania	38 000 (26 000–53 400)	347·0 (237·0–484·0)	2140 (805–3340)	16.1 (6.5–24.4)	40 200 (28 300–55 700)	363.0 (255.0–501.0)
American Samoa	221 (154–311)	420·0 (296·0–587·0)	22.1 (13.6–40)	40.5 (24.0–76.7)	243 (170–340)	460.0 (324.0–639.0)
Cook Islands	92.8 (63.8–135)	438·0 (303·0–643·0)	0.0337 (0.0272–0.0435)	0.2 (0.1–0.2)	92.8 (63.9–135)	438.0 (304.0–643.0)
Fiji	3800 (2600–5310)	425·0 (291·0–584·0)	58.6 (42.7–81.7)	6.6 (4.8–9.2)	3860 (2660–5360)	431.0 (298.0–591.0)
Guam	834 (546–1190)	456·0 (303·0–650·0)	60 (51.5–69.1)	34.4 (29.5–39.8)	894 (605–1250)	491.0 (337.0–685.0)
Kiribati	390 (273–547)	398·0 (277·0–544·0)	3.07 (1.96–3.91)	2.7 (1.7–3.5)	393 (276–550)	401.0 (280.0–546.0)
Marshall Islands	191 (132–273)	376·0 (259·0–525·0)	12.9 (5.56–19.2)	22.3 (9.8–32.7)	204 (146–288)	399.0 (285.0–550.0)
Micronesia (Federated States of)	351 (240–504)	380·0 (262·0–538·0)	24.4 (10.6–37.1)	23.6 (10.3–35.5)	375 (267–532)	404.0 (287.0–565.0)
Nauru	30.7 (21.2–44.9)	384·0 (269·0–544·0)	2.94 (1.19–4.4)	27.8 (11.4–41.6)	33.6 (24.3–48.1)	413.0 (303.0–573.0)
Niue	8.13 (5.68–11.3)	425·0 (301·0–588·0)	0.274 (0.114–0.402)	16.3 (6.6–24.2)	8.4 (5.92–11.6)	442.0 (315.0–604.0)
Northern Mariana Islands	229 (148–334)	421·0 (284·0–602·0)	5.49 (3.87–7.29)	11.9 (8.7–16.3)	234 (154–338)	438.0 (299.0–626.0)
Palau	88.8 (60.4–129)	382·0 (263·0–551·0)	1 (0.756–1.29)	6.2 (4.6–8.1)	89.8 (61.6–130)	389.0 (271.0–558.0)
Papua New Guinea	26 400 (18 100–37 100)	328·0 (225·0–461·0)	1580 (480–2670)	15.4 (4.9–25.5)	28 000 (19 600–39 100)	344.0 (243.0–479.0)
Samoa	707 (476–954)	395·0 (267·0–532·0)	39.3 (17.1–64.3)	18.4 (8.0–30.4)	747 (519–995)	414.0 (287.0–551.0)
Solomon Islands	1600 (1080–2310)	309·0 (208·0–440·0)	110 (41.6–170)	17.6 (7.0–27.0)	1710 (1190–2430)	327.0 (227.0–460.0)

Tokelau	5.68<br>(3.89–7.98)	410·0<br>(282·0–577·0)	0.219<br>(0.0875–0.324)	15.7<br>(6.2–23.1)	5.9<br>(4.14–8.18)	429.0<br>(302.0–596.0)
Tonga	370<br>(257–516)	427·0<br>(296·0–592·0)	60.1<br>(45.3–79.1)	62.4<br>(47.6–80.9)	430<br>(315–577)	489.0<br>(356.0–652.0)
Tuvalu	44.5<br>(30.3–61.8)	388·0<br>(265·0–540·0)	2.18<br>(0.96–3.23)	17.9<br>(7.9–26.3)	46.6<br>(33.1–63.9)	406.0<br>(289.0–558.0)
Vanuatu	873<br>(595–1230)	370·0<br>(251·0–520·0)	54.5<br>(18–90.4)	18.4<br>(6.1–30.4)	928<br>(649–1300)	388.0<br>(270.0–541.0)
Southeast Asia	3 440 000<br>(2 370 000–4 960 000)	474·0<br>(327·0–674·0)	206 000<br>(167 000–236 000)	29.7<br>(24.0–34.0)	3 640 000<br>(2 580 000–5 170 000)	503.0<br>(357.0–705.0)
Cambodia	64 700<br>(43 500–93 300)	406·0<br>(274·0–583·0)	7160<br>(3500–11 300)	42.7<br>(20.9–66.1)	71 900<br>(51 400–100 000)	449.0<br>(320.0–625.0)
Indonesia	1 340 000<br>(924 000–1 930 000)	483·0<br>(333·0–681·0)	28 600<br>(17 500–37 400)	11.1<br>(6.5–14.4)	1 370 000<br>(955 000–1 950 000)	494.0<br>(345.0–692.0)
Laos	29 200<br>(19 700–41 100)	454·0<br>(309·0–631·0)	3450<br>(1780–5150)	46.2<br>(23.9–67.6)	32 700<br>(23 400–46 200)	500.0<br>(358.0–697.0)
Malaysia	168 000<br>(116 000–245 000)	510·0<br>(351·0–740·0)	13 100<br>(10 800–15 800)	7620<br>(5390–<br>10 600)	181 000<br>(128 000–258 000)	550.0<br>(389.0–779.0)
Maldives	2660<br>(1800–3770)	473·0<br>(324·0–656·0)	83.5<br>(56–131)	16.7<br>(11.9–23.9)	2750<br>(1860–3880)	490.0<br>(342.0–678.0)
Mauritius	8470<br>(5880–12 000)	521·0<br>(362·0–742·0)	968<br>(815–1120)	67.8<br>(57.1–78.9)	9440<br>(6920–13 000)	589.0<br>(434.0–807.0)
Myanmar	244 000<br>(164 000–350 000)	436·0<br>(295·0–618·0)	24 400<br>(14 400–30 600)	43.5<br>(25.5–54.4)	268 000<br>(191 000–381 000)	480.0<br>(342.0–673.0)
Philippines	403 000<br>(277 000–576 000)	395·0<br>(272·0–559·0)	48 500<br>(41 000–57 900)	43.4<br>(36.8–51.8)	452 000<br>(326 000–622 000)	439.0<br>(315.0–600.0)
Seychelles	625<br>(432–863)	518·0<br>(358·0–703·0)	6.06<br>(4.5–7.32)	5.5<br>(4.1–6.8)	631<br>(438–869)	523.0<br>(363.0–708.0)
Sri Lanka	115 000<br>(78 200–165 000)	459·0<br>(312·0–657·0)	1360<br>(975–1860)	5.9<br>(4.3–8.2)	116 000<br>(79 800–166 000)	465.0<br>(319.0–663.0)
Thailand	574 000<br>(401 000–822 000)	613·0<br>(434·0–883·0)	53 800<br>(42 600–68 300)	67.2<br>(52.7–85.1)	627 000<br>(449 000–872 000)	681.0<br>(498.0–947.0)

Timor-Leste	4140 (2850–6190)	403·0 (278·0–605·0)	484 (204–934)	36.8 (15.3–74.6)	4630 (3280–6860)	440.0 (307.0–657.0)
Viet Nam	480 000 (326 000–698 000)	428·0 (290·0–611·0)	23 800 (12 300–35 100)	23.7 (11.9–34.5)	503 000 (351 000–723 000)	452.0 (315.0–635.0)
Sub-Saharan Africa	2 360 000 (1 620 000–3 310 000)	323·0 (221·0–450·0)	211 000 (150 000–262 000)	25.2 (18.3–31.5)	2 570 000 (1 840 000–3 540 000)	348.0 (248.0–477.0)
Central sub-Saharan Africa	264 000 (182 000–375 000)	303·0 (210·0–422·0)	26 100 (13 100–40 600)	28.4 (14.3–44.6)	290 000 (205 000–407 000)	332.0 (239.0–458.0)
Angola	55 400 (36 300–82 000)	294·0 (199·0–419·0)	5870 (2780–9870)	28.2 (13.5–48.0)	61 200 (41 200–90 500)	322.0 (222.0–459.0)
Central African Republic	9320 (6100–13 400)	273·0 (186·0–382·0)	1310 (656–2140)	34.3 (17.3–55.4)	10 600 (7410–14 700)	307.0 (226.0–414.0)
Congo	13 800 (9150–20 200)	332·0 (223·0–470·0)	1650 (882–2600)	39.7 (21.4–61.0)	15 400 (11 200–21 700)	372.0 (270.0–508.0)
Democratic Republic of the Congo	176 000 (121 000–250 000)	303·0 (210·0–421·0)	16 400 (7610–26 800)	27.0 (12.4–45.1)	193 000 (138 000–270 000)	330.0 (240.0–458.0)
Equatorial Guinea	3630 (2560–4980)	385·0 (273·0–522·0)	328 (157–577)	34.6 (17.6–58.2)	3950 (2880–5310)	419.0 (311.0–561.0)
Gabon	5550 (3790–7840)	372·0 (254·0–516·0)	550 (303–836)	38.5 (21.6–57.4)	6100 (4400–8430)	411.0 (299.0–558.0)
Eastern sub-Saharan Africa	822 000 (562 000–1 170 000)	305·0 (208·0–428·0)	46 200 (27 500–73 300)	15.6 (9.2–25.3)	868 000 (605 000–1 230 000)	321.0 (223.0–446.0)
Burundi	14 000 (7 880–22 700)	191·0 (113·0–309·0)	1380 (713–2430)	15.6 (8.2–27.8)	15 400 (9470–24 300)	207.0 (126.0–329.0)
Comoros	2160 (1510–2990)	347·0 (242·0–476·0)	111 (62–196)	17.5 (9.9–30.8)	2280 (1620–3130)	365.0 (260.0–498.0)
Djibouti	3400 (2390–4820)	333·0 (234·0–456·0)	120 (51.5–244)	13.3 (6.1–26.5)	3520 (2490–4940)	347.0 (248.0–470.0)
Eritrea	9900 (4360–17 000)	208·0 (95·4–350·0)	798 (348–1350)	16.7 (7.4–29.2)	10 700 (5070–17 800)	224.0 (112.0–367.0)
Ethiopia	202 000 (132 000–292 000)	297·0 (199·0–418·0)	9610 (5480–20 200)	12.7 (7.2–26.4)	212 000 (140 000–302 000)	309.0 (208.0–431.0)

Kenya	125 000 (86 300–177 000)	345·0 (237·0–482·0)	5210 (3390–8230)	14.7 (9.5–23.3)	130 000 (90 700–183 000)	360.0 (249.0–498.0)
Madagascar	62 800 (44 000–87 900)	339·0 (237·0–469·0)	2930 (1720–5100)	14.0 (7.9–24.1)	65 800 (47 000–91 200)	7 420 (5 370–10 400)
Malawi	40 700 (28 700–55 500)	337·0 (238·0–460·0)	2710 (1520–4340)	20.1 (11.0–33.9)	43 400 (31 100–58 500)	357.0 (257.0–483.0)
Mozambique	51 100 (35 100–74 400)	284·0 (197·0–410·0)	4380 (2250–8510)	21.7 (11.3–42.4)	55 500 (38 700–81 300)	305.0 (218.0–443.0)
Rwanda	18 200 (8800–31 300)	192·0 (96·8–329·0)	1530 (855–2850)	15.8 (8.9–29.2)	19 700 (10 100–32 700)	208.0 (109.0–344.0)
Somalia	27 500 (17 700–40 600)	236·0 (155·0–342·0)	2600 (1370–3890)	19.8 (10.6–29.8)	30 100 (20 100–43 600)	256.0 (172.0–364.0)
South Sudan	15 600 (10 400–22 600)	271·0 (188·0–382·0)	1460 (803–2160)	20.8 (11.8–31.5)	17 100 (12 000–24 300)	292.0 (209.0–406.0)
Uganda	76 600 (51 400–109 000)	312·0 (210·0–441·0)	4350 (2390–7870)	7620 (5390–10 600)	80 900 (55 100–115 000)	327.0 (223.0–462.0)
Tanzania	129 000 (89 900–178 000)	337·0 (232·0–461·0)	6520 (3800–11 100)	15.4 (9.2–26.2)	136 000 (97 000–187 000)	353.0 (249.0–482.0)
Zambia	42 200 (29 000–59 100)	346·0 (236·0–474·0)	2410 (1500–3650)	18.2 (11.3–26.4)	44 600 (31 600–61 800)	364.0 (256.0–494.0)
Southern sub-Saharan Africa	257 000 (177 000–363 000)	355·0 (245·0–495·0)	29 700 (24 400–35 100)	41.4 (34.1–48.2)	287 000 (207 000–392 000)	397.0 (286.0–536.0)
Botswana	7620 (5390–10 600)	361·0 (254·0–491·0)	477 (275–690)	23.7 (14.4–33.6)	8090 (5780–11 200)	385.0 (273.0–523.0)
Eswatini	3020 (2080–4330)	344·0 (236·0–487·0)	438 (233–714)	50.0 (28.6–83.2)	3460 (2540–4740)	394.0 (288.0–533.0)
Lesotho	5570 (3760–7710)	315·0 (211·0–431·0)	759 (498–1090)	44.1 (29.3–63.9)	6330 (4560–8870)	359.0 (262.0–495.0)
Namibia	6940 (4750–9870)	355·0 (245·0–499·0)	480 (264–813)	25.3 (14.2–41.1)	7420 (5370–10 400)	380.0 (276.0–527.0)

South Africa	197 000 (136 000–281 000)	358·0 (247·0–504·0)	24 500 (19 900–29 800)	45.0 (36.6–53.9)	222 000 (160 000–306 000)	403.0 (290.0–548.0)
Zimbabwe	36 700 (25 300–49 200)	340·0 (236·0–451·0)	3080 (1350–4300)	28.1 (11.8–39.2)	39 800 (29 500–52 300)	368.0 (275.0–479.0)
Western sub-Saharan Africa	1 010 000 (695 000–1 420 000)	335·0 (230·0–466·0)	109 000 (74 100–138 000)	28.3 (20.5–34.8)	1 120 000 (814 000–1 530 000)	364.0 (260.0–495.0)
Benin	26 000 (17 500–36 500)	320·0 (217·0–448·0)	3220 (2190–4510)	29.5 (20.6–40.7)	29 200 (21 300–40 300)	350.0 (254.0–483.0)
Burkina Faso	44 500 (30 400–61 600)	302·0 (205·0–414·0)	6860 (4340–9400)	35.0 (23.6–47.0)	51 300 (37 100–70 300)	337.0 (242.0–454.0)
Cabo Verde	1900 (1290–2670)	350·0 (237·0–487·0)	72 (53–94.8)	14.8 (10.9–19.3)	1970 (1360–2750)	364.0 (251.0–502.0)
Cameroon	69 000 (47 400–97 900)	340·0 (235·0–476·0)	8930 (5490–12 600)	36.2 (23.6–51.0)	77 900 (56 000–108 000)	377.0 (273.0–517.0)
Chad	25 400 (17 500–35 900)	274·0 (191·0–383·0)	3350 (2250–5410)	23.9 (16.4–37.3)	28 800 (21 300–39 300)	298.0 (220.0–408.0)
Côte d'Ivoire	60 600 (42 000–85 700)	324·0 (222·0–441·0)	364.0 (251.0–502.0)	26.9 (18.3–36.7)	66 600 (48 200–92 000)	351.0 (253.0–469.0)
Gambia	13 100 (10 800–15 800)	329·0 (224·0–449·0)	492 (328–686)	27.0 (18.7–36.9)	5580 (4030–7570)	356.0 (257.0–480.0)
Ghana	92 200 (65 100–130 000)	368·0 (258·0–514·0)	7210 (5080–9890)	28.6 (21.0–38.3)	99 400 (72 400–139 000)	397.0 (287.0–547.0)
Guinea	25 600 (17 800–34 900)	311·0 (215·0–424·0)	2890 (1840–4530)	26.5 (17.3–39.9)	28 400 (20 500–38 200)	337.0 (244.0–454.0)
Guinea-Bissau	3960 (2700–5790)	308·0 (210·0–439·0)	560 (377–824)	37.8 (25.3–53.0)	4520 (3290–6360)	346.0 (251.0–479.0)
Liberia	10 000 (6700–14 400)	290·0 (202·0–401·0)	1490 (885–2070)	34.3 (21.4–46.3)	11 500 (8450–16 000)	324.0 (240.0–437.0)
Mali	37 800 (25 300–54 200)	283·0 (188·0–400·0)	6240 (4000–8920)	32.5 (22.3–44.5)	44 100 (30 900–61 000)	315.0 (222.0–437.0)
Mauritania	10 300 (7090–14 200)	350·0 (242·0–478·0)	763 (499–1050)	23.4 (16.1–31.9)	11 000 (7750–15 000)	373.0 (263.0–502.0)
Niger	35 300 (23 700–49 700)	275·0 (185·0–383·0)	4770 (3190–6740)	25.4 (17.7–35.8)	40 100 (29 300–54 700)	300.0 (214.0–409.0)

Nigeria	491 000 (336 000–694 000)	353·0 (242·0–492·0)	48 600 (29 600–64 600)	26.7 (17.3–35.4)	540 000 (386 000–744 000)	380.0 (268.0–520.0)
São Tomé and Príncipe	550 (369–759)	334·0 (224·0–454·0)	44.6 (28.3–65.7)	25.8 (17.2–35.4)	594 (414–803)	360.0 (251.0–480.0)
Senegal	36 400 (24 900–50 100)	333·0 (229·0–459·0)	3370 (2280–4780)	27.2 (19.5–36.6)	39 800 (28 500–53 300)	360.0 (258.0–485.0)
Sierra Leone	17 100 (11 800–24 200)	299·0 (208·0–413·0)	2120 (1310–3190)	28.0 (18.8–39.9)	19 300 (14 200–26 800)	327.0 (238.0–448.0)
Togo	19 600 (13 400–27 600)	332·0 (229·0–461·0)	1810 (1290–2490)	28.1 (20.1–37.8)	21 400 (15 600–29 300)	360.0 (264.0–488.0)

Supplemental Table 8: Forecast of other MSK age-standardised prevalence and total cases globally and by region, from 2020 to 2050

	Age-standardised prevalence rate (%, 95% UI)			Cases (millions, 95% UI)		
**Region**	**2030**	**2040**	**2050**	**2030**	**2040**	**2050**
Global	6·96 (6·24–7·85)	8·12 (7·42–9·05)	9·20 (8·43–10·1)	670 (606–750)	869 (790–965)	1060 (964–1170)
Andean Latin America	7·22 (6·43–8·15)	8·07 (7·27–9·03)	8·89 (8·09–9·88)	5·58 (5·05–6·28)	7·35 (6·67–8·12)	9·16 (8·27–10·1)
Australasia	8·00 (7·15–9·06)	8·58 (7·64–9·63)	9·16 (8·18–10·2)	3·42 (3·00–3·88)	4·00 (3·54–4·53)	4·62 (4·09–5·20)
Caribbean	5·45 (4·83–6·17)	6·00 (5·34–6·75)	6·58 (5·90–7·41)	3·12 (2·76–3·50)	3·67 (3·26–4·12)	4·17 (3·65–4·75)
Central Asia	2·50 (2·13–2·99)	2·68 (2·30–3·18)	2·86 (2·47–3·38)	2·68 (2·29–3·20)	3·27 (2·85–3·82)	3·80 (3·28–4·44)
Central Europe	2·16 (1·84–2·52)	2·25 (1·91–2·61)	2·32 (1·98–2·68)	3·10 (2·64–3·75)	3·03 (2·57–3·67)	2·81 (2·40–3·39)
Central Latin America	9·44 (8·46–10·6)	10·7 (9·70–11·9)	11·8 (10·8–13·0)	29·4 (26·6–32·8)	38·3 (35·0–42·5)	46·1 (42·2–51·3)
Central sub-Saharan Africa	4·43 (3·91–5·11)	5·34 (4·77–6·09)	6·22 (5·43–7·15)	5·36 (4·71–6·28)	9·01 (7·79–10·5)	13·8 (11·6–16·1)
East Asia	5·35 (4·75–6·05)	6·23 (5·60–6·94)	7·28 (6·61–8·08)	111 (97·8–126)	128 (114–144)	140 (124–156)
Eastern Europe	1·96 (1·58–2·40)	2·05 (1·68–2·50)	2·13 (1·75–2·58)	5·49 (4·42–6·86)	5·55 (4·58–6·85)	5·41 (4·45–6·60)
Eastern sub-Saharan Africa	4·32 (3·79–4·98)	5·19 (4·59–5·86)	6·08 (5·36–6·81)	16·4 (14·5–18·8)	27·3 (23·9–31·1)	42·5 (35·9–50·4)
High income Asia Pacific	8·77 (7·73–9·97)	9·35 (8·32–10·6)	9·88 (8·85–11·1)	23·5 (20·5–26·4)	23·8 (20·8–26·5)	23·1 (20·4–26·2)
High income North America	11·4 (10·8–12·2)	11·8 (11·2–12·5)	12·2 (11·6–12·9)	54·5 (51·1–58·2)	57·9 (54·4–61·5)	61·3 (57·2–65·0)
North Africa and Middle East	5·54 (4·89–6·37)	6·51 (5·81–7·42)	7·38 (6·59–8·39)	41·6 (36·8–47·7)	59·1 (52·7–66·6)	76·3 (68·5–86·0)
Oceania	4·48 (3·92–5·21)	5·15 (4·57–5·91)	5·97 (5·37–6·73)	0·618 (0·542–0·706)	0·894 (0·793–0·990)	1·28 (1·14–1·45)
South Asia	10·6 (9·55–11·7)	12·7 (11·7–14·0)	14·6 (13·5–15·9)	226 (206–250)	320 (296–350)	406 (374–446)
Southeast Asia	6·24 (5·58–7·08)	7·13 (6·46–8·00)	8·06 (7·35–8·96)	53·0 (47·9–59·8)	67·5 (61·2–75·9)	80·7 (73·4–89·7)
Southern Latin America	11·4 (10·1–12·7)	12·3 (11·0–13·8)	13·2 (11·9–14·7)	9·77 (8·72–10·9)	11·5 (10·5–12·6)	13·0 (11·8–14·1)
Southern sub-Saharan Africa	4·65 (4·08–5·32)	5·18 (4·59–5·88)	5·69 (5·09–6·42)	4·06 (3·61–4·63)	5·40 (4·80–6·10)	6·80 (5·96–7·73)
Tropical Latin America	8·31 (7·39–9·34)	9·43 (8·49–10·5)	10·5 (9·52–11·6)	24·7 (22·2–27·7)	30·8 (27·9–34·5)	35·7 (32·5–40·3)
Western Europe	4·60 (3·90–5·36)	4·91 (4·21–5·67)	5·22 (4·53–6·00)	26·2 (21·9–31·6)	28·2 (23·9–33·7)	29·6 (25·2–35·0)
Western sub-Saharan Africa	4·80 (4·30–5·44)	5·87 (5·33–6·55)	6·99 (6·34–7·74)	20·3 (18·1–22·8)	34·6 (30·6–39·5)	55·2 (48·0–64·3)

Supplemental Figure 1: Unique source-years of non-fatal data for other MSK

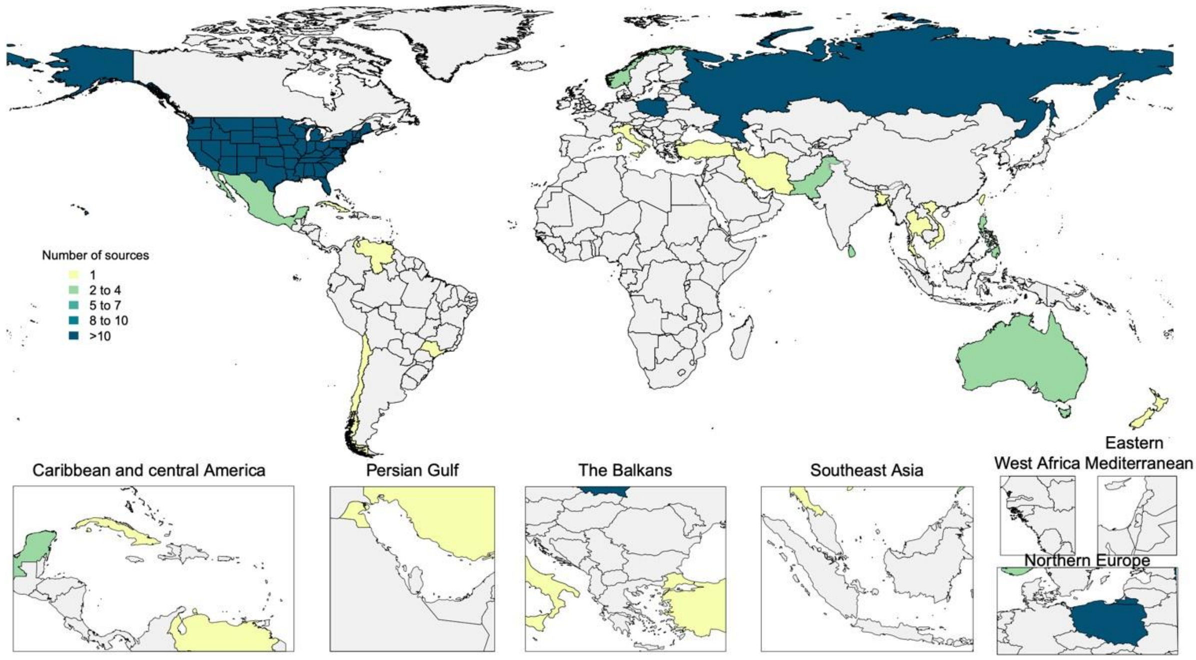

Note: There are >10 sources for both all states in the USA and the USA at a national level

Supplemental Figure 2: Unique source-years of cause of death data for other MSK

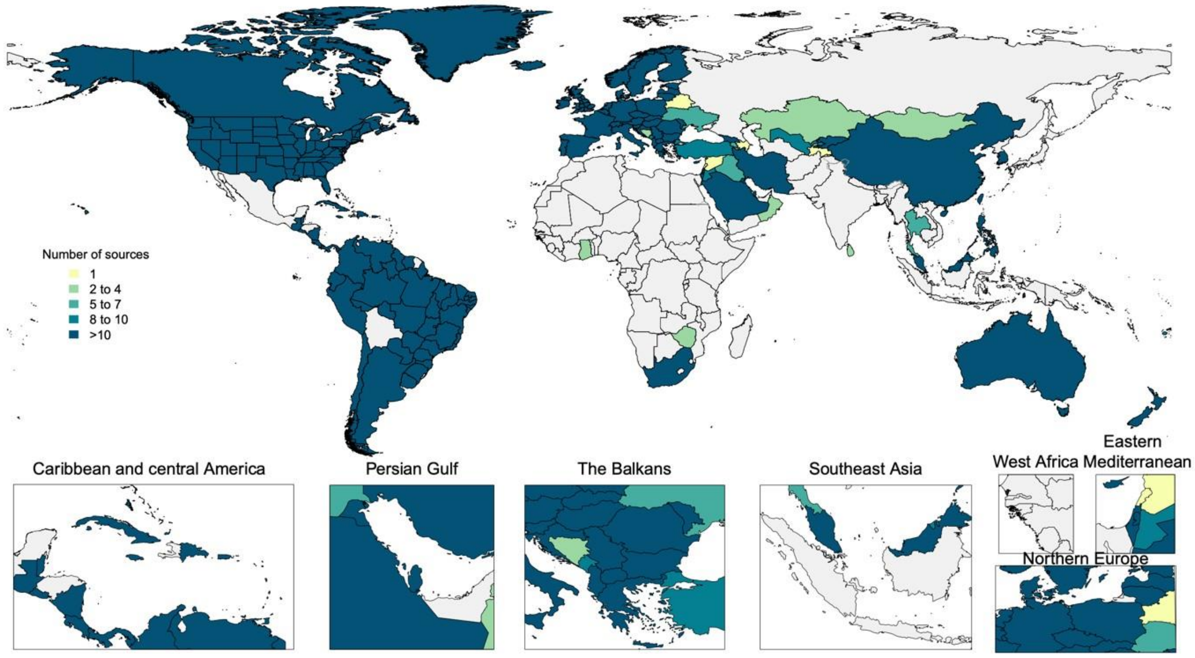

Note: There are >10 sources for both all states in the USA and the USA at a national level

Supplemental Figure 3: Global forecast of other MSK conditions to 2050, males and females

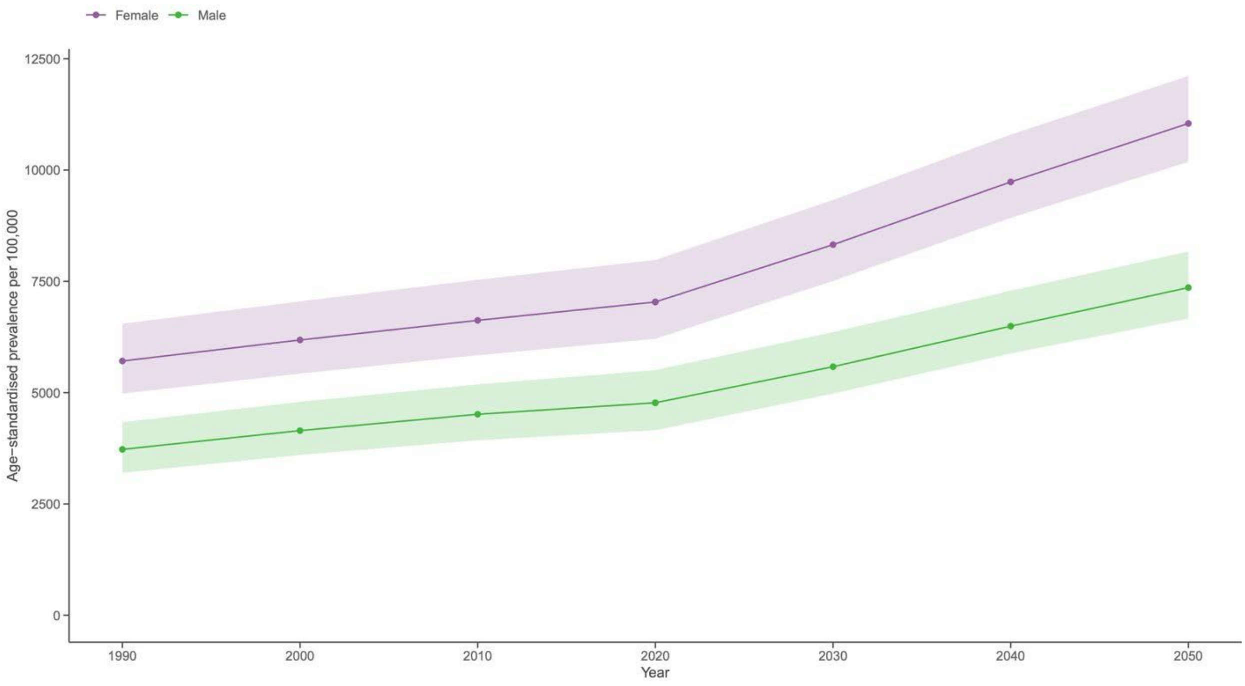

Supplemental Figure 4: Forecast of other MSK by region to 2050

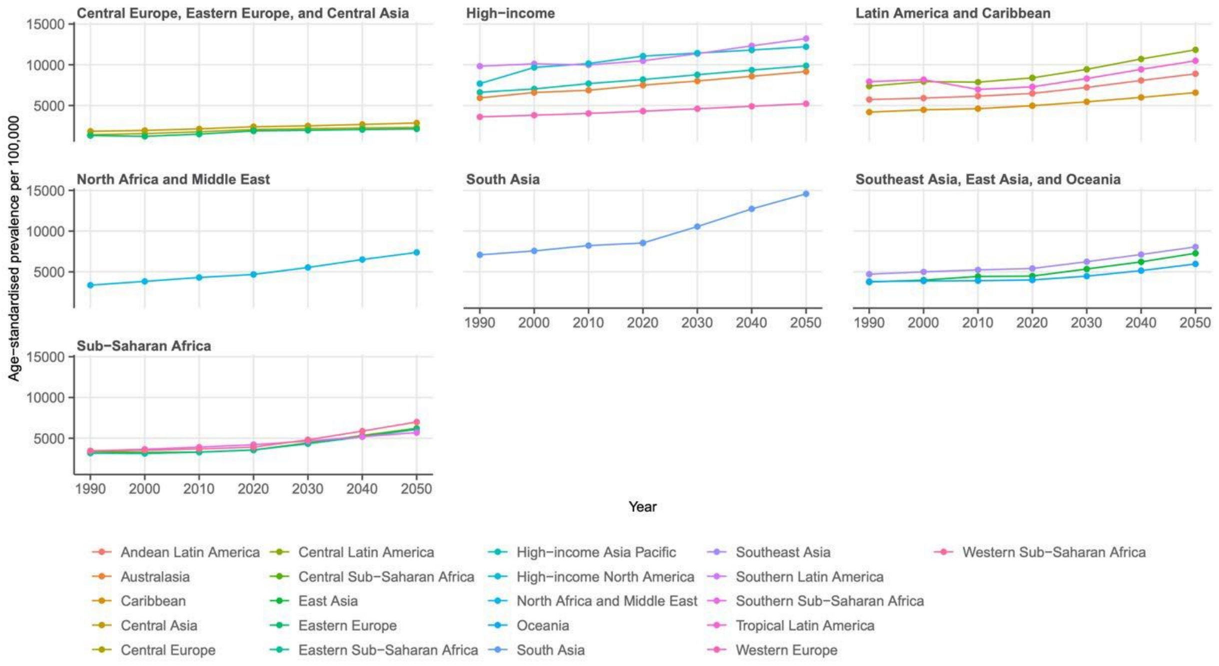

Supplement: Supplementary appendix [file mmc1.pdf]
